# Supplementary material for: Discovery of 1,2,4-Oxadiazole Derivatives Containing Haloalkyl as Potential Acetylcholine Receptor Nematicides
Source: Int J Mol Sci. 2023 Mar 17;24(6):5773. doi: 10.3390/ijms24065773 (PMC10058719; doi:10.3390/ijms24065773)
Supplement: Supplementary file 1 [file ijms-24-05773-s001.zip › Supporting Information File S1.pdf]

Supplementary Materials

Discovery of 1,2,4-Oxadiazole Derivatives Containing Haloalkyl as Potential Acetylcholine Receptor Nematicides

Ling Luo, Yuqin Ou, Qi Zhang and Xiuhai Gan *

National Key Laboratory of Green Pesticide, Key Laboratory of Green Pesticide and Agricultural Bioengineering, Ministry of Education, Guizhou University, Guiyang 550025, China

* Correspondence: xhgan3@gzu.edu.cn

Contents

S1. General Information 2

S2. General procedure for preparation intermediates **1** 2

S3. General synthetic procedure for title compounds **A1-A29** and **B1-B19** 2

S4. Binding Free Energies Calculations 2

S5. Spectral date of target compounds 3

S1. General Information

The progress of the reaction was monitored by thin-layer chromatography (TLC) on silica GF_{254} and the melting points of the compounds were determined by X-4B microscope melting point apparatus (Shanghai Electrophysics Optical Instrument Co., Ltd., Shanghai, China). ^1H NMR and ^{13}C NMR were conducted on a Bruker DPX-400 or DPX-500 spectrometer (Bruker, Billerica, MA, United States) with $CDCl_3$ as the solvent, tetramethyl silane was used an internal standard. The high-resolution mass spectrometer (HRMS) data were tested on Thermo Scientific Q Exactive(Thermo Scientific, Missouri, MO, USA).

S2. General procedure for preparation intermediates 1

50% aqueous solution of sodium hydroxide (1.5 mmol) was added to an ethanolic solution of hydroxylamine hydrochloride (1.5 mmol), stirred at room temperature for 10 min. Differently substituted benzonitrile (1.0 mmol) or heterocyclic nitrile was dissolved in ethanol, added to the above mixture, and then warmed to 80 °C and stirred for 7 h. The reaction was monitored with thin-layer chromatography (TLC). After the reaction was completed, the generated solid was removed by filtration, the filtrate was concentrated under vacuum, and then the mixture was washed with saturated NaCl solution (30 mL), extracted with ethyl acetate (30 mL) for three times, dried with anhydrous Na_2SO_4, filtered and concentrated under vacuum to give the intermediates **1** in yields of above 80.0–95.0%.

S3. General synthetic procedure for title compounds A1-A29 and B1-B19

To a stirred solution of intermediates **1** (1.0 mmol) and triethylamine (1.5 mmol) in toluene was added various acyl chloride or acyl bromide (1.5 mmol) under ice bath and the reaction mixture was stirred above 10 h at 110 °C. Then, the reaction was monitored by TLC. Upon completion of reaction, the reaction mixture was washed with saturated sodium chloride solution and then extracted with 30 mL of ethyl acetate (EA) for three times. The organic layer was concentrated in a vacuum to obtain crude product. Residue was purified by silica-gel column chromatography using petroleum ether/ethyl acetate (5:1/v:v) to obtain target compounds **A1-A29** and **B1-B19**.

S4. Binding Free Energies Calculations

The MM-PBSA method was used to calculate the binding free energy (ΔG_{bind}) between receptor and ligand. It is gotten by calculating the difference of free energies between ligand-receptor complex (G_{cpx}) and the unbound receptor (G_{rec}) and ligand (G_{lig}) as the following:

$$\Delta G_{bind} = G_{cpx} - (G_{cpx} + G_{lig})$$

The ΔG_{bind} consists of the molecular mechanical (MM) gas-phase binding energy (ΔE_{MM}), solvation free energy (ΔG_{sol}) and entropic contribution ($-T\Delta S$):

$$\Delta G_{bind} = \Delta E_{MM} + \Delta G_{sol} - T\Delta S$$

The ΔE_{MM} includes two parts, the electrostatic energies (ΔE_{ele}) and van der Waals interaction (ΔE_{VdW}):

$$\Delta E_{MM} = \Delta E_{ele} + \Delta E_{VdW}$$

The ΔG_{sol} is made up of electrostatic contribution (ΔG_{PB}) and nonelectrostatic contribution (ΔG_{np}) to the solvation free energy. ΔG_{PB} is calculated by the Poisson-Boltzman (PB) method using MM-PBSA module in amber16 program. ΔG_{np} is determined by the solvent accessible surface area.

$$\Delta G_{sol} = \Delta G_{PB} + \Delta G_{np}$$

For the entropic contribution, an empirical method was used, it consists two subitems, the solvation entropy change (ΔS_{sol}) and conformational entropy change (ΔS_{conf}):

$$\Delta S = \Delta S_{sol} + \Delta S_{conf}$$

The ΔS_{sol} is obtained by the tendency of water molecules to minimize their contacts with hydrophobic groups in protein, ΔS_{conf} is related to the change of the number of rotatable bonds during the binding process. The entropic contribution is evaluated and the conformational entropy change is proportional to the number (ΔN_{rot}) of the lost rotatable bonds during the binding:

$$-T\Delta S_{conf} = w(\Delta N_{rot})$$

in which w is a scaling factor which was set to be 1 Kcal/mol for the binding energy calculation. Thus, the equation (2) can be written as:

$$\Delta G_{bind} = \Delta E_{MM} + \Delta G_{PB} + \Delta G_{np} - T\Delta S_{sol} + w(\Delta N_{rot})$$

all other parameters in the energy calculation are the standard parameters or the default values of the Amber18 program.

S5. Spectral date of target compounds

Data for 5-(chloromethyl)-3-(4-fluorophenyl)-1,2,4-oxadiazole (**A1**), white solid, m.p. 26.6–27.6 °C, yield, 76.1%; ^{1}H NMR (400 MHz, $CDCl_3$) δ 8.08 (ddd, *J* = 8.1, 5.2, 2.5 Hz, 2H, Ar-H), 7.18 (ddd, *J* = 9.7, 5.9, 2.5 Hz, 2H, Ar-H), 4.74 (s, 2H, -CH_2Cl). ^{13}C NMR (101 MHz, $CDCl_3$) δ 174.44, 168.08, 164.78 (d, *J* = 252.5 Hz), 129.72 (d, *J* = 9.1 Hz), 129.72 (d, *J* = 9.1 Hz), 122.40 (d, *J* = 4.0 Hz), 116.21 (d, *J* = 21.2 Hz), 33.31. HRMS (ESI) m/z for $C_9H_6ClFN_2O$ $[M+H]^+$ calcd: 213.02255, found: 213.02232.

Data for 5-(chloromethyl)-3-(4-chlorophenyl)-1,2,4-oxadiazole (**A2**), white solid, m.p. 56.2–58.1 °C, yield, 71.0%; ^{1}H NMR (400 MHz, $CDCl_3$) δ 8.02 (d, *J* = 8.4 Hz, 2H, Ar-H), 7.47 (d, *J* = 8.8 Hz, 2H, Ar-H), 4.74 (s, 2H, -CH_2Cl). ^{13}C NMR (101 MHz, $CDCl_3$) δ 174.54, 168.09, 137.76, 129.30, 129.30, 128.80, 128.80, 124.64, 33.32. HRMS (ESI) m/z for $C_9H_6Cl_2N_2O$ $[M+H]^+$ calcd: 228.99299, found: 228.99292.

Data for 3-(4-bromophenyl)-5-(chloromethyl)-1,2,4-oxadiazole (**A3**), white solid, m.p. 56.0–56.9 °C, yield, 68.0%; ^{1}H NMR (400 MHz, $CDCl_3$) δ 7.98–7.94 (m, 2H, Ar-H), 7.66–7.62 (m, 2H, Ar-H), 4.75 (s, 2H, -CH_2Cl). ^{13}C NMR (101 MHz, $CDCl_3$) δ 174.56, 168.21, 132.29, 132.29, 128.99, 128.99, 126.21, 125.10, 33.30. HRMS (ESI) m/z for $C_9H_6BrClN_2O$ $[M+H]^+$ calcd: 270.92683, found: 270.92792.

Data for 5-(bromomethyl)-3-(4-fluorophenyl)-1,2,4-oxadiazole (**A4**), yellow solid, m.p. 34.3–34.5 °C, yield, 60.3%; ^{1}H NMR (400 MHz, CDCl3) δ 8.09 (dd, *J* = 8.8, 5.2 Hz, 2H, Ar-H), 7.18 (t, *J* = 8.8 Hz, 2H, Ar-H), 4.55 (s, 2H, -CH_2Br). ^{13}C NMR (101 MHz, $CDCl_3$) δ 174.71, 168.16, 164.76 (d, *J* = 252.5 Hz), 129.70 (d, *J* = 4.0 Hz), 129.70 (d, *J* = 4.0 Hz), 122.46, 116.19 (d, *J* = 22.2 Hz), 116.08, 16.36. HRMS (ESI) m/z for $C_9H_6BrFN_2O$ $[M+H]^+$ calcd: 256.97203, found: 256.97192.

Data for 5-(chloromethyl)-3-(2-fluorophenyl)-1,2,4-oxadiazole (**A5**), yellow oil, yield 75.4 %; ^{1}H NMR (500 MHz, $CDCl_3$) δ 8.03 (td, J = 7.5, 1.5 Hz, 1H, Ar-H), 7.52-7.48 (m, 1H, Ar-H), 7.27 (td, J = 6.5, 1.0 Hz, 1H, Ar-H), 7.23 (ddd, J = 10.5, 8.5, 1.0 Hz, 1H, Ar-H), 4.77 (s, 2H, -CH_2Cl). ^{13}C NMR (101 MHz, $CDCl_3$) δ 173.99, 168.45, 134.32 (d, *J* = 3.0 Hz), 133.90, 133.21, 132.07 (d, *J* = 15.6 Hz), 127.58 (d, *J* = 11.1 Hz), 123.73 (d, *J* = 325.22 Hz), 33.36. HRMS (ESI) m/z for $C_9H_6ClFN_2O$ $[M+H]^+$ calcd: 213.02255, found: 213.02234.

Data for 5-(chloromethyl)-3-(6-chloropyridin-3-yl)-1,2,4-oxadiazole (**A6**), white solid, m.p. 107.7–108.1 °C, yield, 85.0%;^{1}H NMR (500 MHz, $CDCl_3$) δ 9.09 (dd, *J* = 2.0, 1.0 Hz, 1H, Ar-H), 8.32 (dd, *J* = 8.5, 2.5 Hz, 1H, Ar-H), 7.48 (dd, *J* = 8.5, 1.0 Hz, 1H, Ar-H), 4.77 (s, 2H, -CH_2Cl). ^{13}C NMR (101 MHz, $CDCl_3$) δ 175.10, 166.22, 154.40, 148.72, 137.26, 124.69, 121.48 , 33.19. HRMS (ESI) m/z for $C_8H_5Cl_2N_3O$ $[M+H]^+$ calcd: 229.98824, found: 229.98814.

Data for 3-(6-bromopyridin-3-yl)-5-(chloromethyl)-1,2,4-oxadiazole (**A7**), yellow solid; m.p. 108.9–109.8 °C, yield, 60.1%; ^{1}H NMR (400 MHz, $CDCl_3$) δ 9.07 (d, *J* = 2.0 Hz, 1H, pyridine-H), 8.30 (dd, *J* = 8.4, 2.4 Hz, 1H, pyridine-H), 7.46 (d, *J* = 8.4 Hz, 1H, pyridine-H), 4.76 (s, 2H, -CH_2Cl). ^{13}C NMR (101 MHz, $CDCl_3$) δ 175.11, 166.21, 154.39, 148.71, 137.26, 124.69, 121.47, 33.20. HRMS (ESI) m/z for $C_8H_5BrClN_3O$ $[M+H]^+$ calcd: 273.93773, found: 273.93689.

Data for 5-(chloromethyl)-3-(3-chlorophenyl)-1,2,4-oxadiazole (**A8**), white solid, m.p. 42.2–42.9 °C, yield, 71.6%; ^{1}H NMR (500 MHz, $CDCl_3$) δ 8.08 (t, *J* = 1.5 Hz, 1H, Ar-H), 7.97 (dt, *J* = 7.5, 1.5 Hz, 1H, Ar-H), 7.50 (ddd, *J* = 8.0, 2.0, 1.5 Hz, 1H, Ar-H), 7.43 (t, *J* = 8.0 Hz, 1H, Ar-H), 4.75 (s, 2H, -CH_2Cl). ^{13}C NMR (101 MHz, $CDCl_3$) δ 174.64, 167.90, 135.07, 131.61, 130.29, 127.84, 127.60, 125.56, 33.31. HRMS (ESI) m/z for $C_9H_6Cl_2N_2O$ $[M+H]^+$ calcd: 228.99299, found: 228.99292.

Data for 5-(bromomethyl)-3-(4-chlorophenyl)-1,2,4-oxadiazole (**A9**), white solid, m.p. 65.1–66.4 °C, yield, 71.9%; ^{1}H NMR (400 MHz, $CDCl_3$) δ 8.02 (d, *J* = 8.4 Hz, 2H, Ar-H), 7.46 (d, *J* = 8.8 Hz, 2H, Ar-H), 4.55 (s,

2H, -CH_2Br). ^{13}C NMR (101 MHz, $CDCl_3$) δ 174.81, 168.19, 137.75, 129.31, 129.31, 128.80, 128.80, 124.69, 16.34. HRMS (ESI) m/z for $C_9H_6BrClN_2O$ $[M+H]^+$ calcd: 272.94248, found: 272.94186.

Data for 5-(bromomethyl)-3-(6-bromopyridin-3-yl)-1,2,4-oxadiazole (**A10**), yellow solid, m.p. 138.5–138.9 °C, yield, 62.2%; ^1H NMR (400 MHz, $CDCl_3$) δ 9.06 (d, *J* = 2.0 Hz, 1H, pyridine-H), 8.20 (dd, *J* = 8.4, 2.4 Hz, 1H, pyridine-H), 7.64 (d, *J* = 8.0 Hz, 1H, pyridine-H), 4.57 (s, 2H, -CH_2Br). ^{13}C NMR (101 MHz, $CDCl_3$) δ 175.42, 166.38, 148.99, 145.24, 136.87, 128.52, 121.88, 16.08. HRMS (ESI) m/z for $C_8H_5Br_2N_3O$ $[M+H]^+$ calcd: 315.87156, found: 315.87219.

Data for 5-(bromomethyl)-3-(4-bromophenyl)-1,2,4-oxadiazole (**A11**), white solid, m.p. 71.0–72.0 °C, yield, 65.6%; ^1H NMR (400 MHz, $CDCl_3$) δ 8.02 (d, *J* = 8.8 Hz, 2H, Ar-H), 7.46 (d, *J* = 8.8 Hz, 2H, Ar-H), 4.55 (s, 2H, -CH_2Br). ^{13}C NMR (101 MHz, $CDCl_3$) δ 174.83, 168.28, 132.28, 132.28, 128.97, 128.97,126.18, 125.14, 16.33. HRMS (ESI) m/z for $C_9H_6Br_2N_2O$ $[M+H]^+$ calcd: 316.89196, found: 316.89197.

Data for 5-(chloromethyl)-3-(2,4-difluorophenyl)-1,2,4-oxadiazole (**A12**), white solid, m.p. 31.8–32.1 °C, yield, 66.8%; ^1H NMR (400 MHz, $CDCl_3$) δ 8.05 (td, J = 6.8, 5.2 Hz, 1H, Ar-H), 7.06–6.95 (m, 2H, Ar-H), 4.76 (s, 2H, -CH_2Cl). ^{13}C NMR (101 MHz, $CDCl_3$)δ 174.22 , 165.97-165.12 (m), 163.14 (dd, *J* =153.5，9.1 Hz), 160.30 (d, *J* = 10.1 Hz), 132.10 (dd, *J* = 10.3, 3.8 Hz), 112.27 (dd, *J* = 17.2, 3.0 Hz), 111.08 (dd, *J* = 10.1, 3.0 Hz), 105.58- 105.18 (m), 33.28. HRMS (ESI) m/z for $C_9H_5ClF_2N_2O$ $[M+H]^+$ calcd: 231.01312, found: 231.01286.

Data for 5-(chloromethyl)-3-(4-(trifluoromethyl)phenyl)-1,2,4-oxadiazole (**A13**), olorless oil, yield, 61.6%; ^1H NMR (400 MHz, $CDCl_3$) δ 8.21 (d, *J* = 8 Hz, 2H, Ar-H), 7.75 (d, *J* = 8 Hz, 2H, Ar-H), 4.77 (s, 2H, -CH_2Cl). ^{13}C NMR (101 MHz, $CDCl_3$) δ 175.14, 167.97, 133.22 (q, *J* = 32.9 Hz), 129.58-127.76 (m), 127.86, 127.86, 128.23–127.66 (m), 125.95 (q, *J* = 3.8 Hz), 125.05-119.63 (m), 16.24. HRMS (ESI) m/z for $C_{10}H_6ClF_3N_2O$ $[M-H]^-$ calcd: 261.00370, found: 261.00480.

Data for 5-(chloromethyl)-3-(6-fluoropyridin-3-yl)-1,2,4-oxadiazole (**A14**), white solid, m.p. 87.0.–87.5 °C, yield, 79.5%; ^1H NMR (400 MHz, $CDCl_3$) δ 8.91 (d, *J* = 2.4 Hz, 1H, Ar-H), 8.44 (ddd, *J* = 8.5, 7.5, 2.4 Hz, 1H, Ar-H), 7.09–7.00 (m, 1H, Ar-H), 4.76 (s, 2H, -CH_2Cl). ^{13}C NMR (101 MHz, $CDCl_3$) δ 175.02, 166.12, 165.13(d, *J* = 245.4 Hz), 147.52 (d, *J* = 16.1 Hz), 140.13 (d, *J* = 8.7 Hz), 120.74 (d, *J* = 4.6 Hz), 110.20 (d, *J* = 37.9 Hz), 33.20. HRMS (ESI) m/z for $C_8H_5ClFN_3O$ $[M+H]^+$ calcd: 214.01779, found: 214.017233.

Data for 5-(chloromethyl)-3-(3-fluorophenyl)-1,2,4-oxadiazole (**A15**), colorless oil, yield, 70.0%; ^1H NMR (500 MHz, $CDCl_3$) δ 7.87 (dt, J = 7.5, 1.5 Hz, 1H, Ar-H), 7.78 (ddd, J = 9.0, 2.5, 1.5 Hz, 1H, Ar-H), 7.46 (td, J = 8.0, 6.0 Hz, 1H, Ar-H), 7.22 (tdd, J = 8.3, 2.6, 0.8 Hz, 1H, Ar-H), 4.75 (s, 2H, -CH_2Cl). ^{13}C NMR (101 MHz, $CDCl_3$) δ 174.62 , 168.04 , 162.87 (d, *J* = 248.5 Hz), 130.70 (d, *J* = 9.09 Hz), 128.15 (d, *J* = 8.1 Hz), 123.22 (d, *J* = 3.0 Hz), 118.56 (d, *J* = 22.2 Hz), 114.55 (d, *J* = 23.2 Hz), 33.29. HRMS (ESI) m/z for $C_9H_6ClFN_2O$ $[M+H]^+$ calcd: 213.02255, found: 213.02245.

Data for 5-(bromomethyl)-3-(2-fluorophenyl)-1,2,4-oxadiazole (**A16**), colorless oil, yield, 75.9%; ^1H NMR (400 MHz, $CDCl_3$) δ 7.97 (td, J = 7.6, 2.0 Hz, 2H, Ar-H), 7.53 (ddd, J = 8.3, 1.8 Hz, 1H, Ar-H), 7.32–7.22 (m, 2H, Ar-H), 4.78 (s, 2H, -CH_2Br). ^{13}C NMR (101 MHz, $CDCl_3$) δ 174.33 (s), 165.82 (d, *J* = 5.1 Hz), 160.69 (d, *J* = 258.6 Hz), 133.14 (d, *J* = 8.1 Hz), 130.71 (d, *J* = 2.0 Hz), 124.54 (d, *J* = 4.0 Hz), 116.79 (d, *J* = 21.2 Hz), 114.51 (d, *J* = 12.1 Hz), 16.31 (s). HRMS (ESI) m/z for $C_9H_6BrFN_2O$ $[M+H]^+$ calcd: 256.97203, found: 256.97186.

Data for 5-(chloromethyl)-3-(2,4-dichlorophenyl)-1,2,4-oxadiazole (**A17**), white solid, m.p. 52.3–54.8 °C, yield 60.8%; ^1H NMR (400 MHz, CDCl3) δ 7.84 (d, *J* = 8.4 Hz, 1H, Ar-H), 7.50 (d, *J* = 2.0 Hz, 1H, Ar-H), 7.32 (dd, *J* = 8.4, 2.0 Hz, 1H, Ar-H), 4.70 (s, 2H, -CH_2Cl). ^{13}C NMR (101 MHz, $CDCl_3$) δ 174.06, 166.99, 137.72, 134.37, 132.55, 131.00, 127.47, 124.01, 33.23. HRMS (ESI) m/z for $C_9H_5Cl_3N_2O$ $[M+H]^+$ calcd: 265.95402, found: 265.95402.

Data for 5-(bromomethyl)-3-(2,4-difluorophenyl)-1,2,4-oxadiazole (**A18**), colorless oil, yield, 71.9 %; ^1H NMR (400 MHz, $CDCl_3$) δ 8.05 (td, *J* = 6.8, 5.2 Hz, 2H, Ar-H), 7.06–6.95 (m, 2H, Ar-H), 4.57 (s, 2H,

-CH_2Br). ^{13}C NMR (101 MHz, $CDCl_3$) δ 174.42, 164.80 (dd, *J* = 255.5, 11.1 Hz), 165.13 (d, *J* = 6.2 Hz), 161.24 (dd, *J* = 260.7, 12.0 Hz), 132.01 (dd, *J* = 10.2, 3.7 Hz), 112.15 (dd, *J* = 21.9, 3.7 Hz), 111.06 (dd, *J* = 12.4, 3.8 Hz), 105.27 (t, *J* = 25.1 Hz), 16.25. HRMS (ESI) m/z for $C_9H_5BrF_2N_2O$ $[M+H]^+$ calcd: 274.96261, found: 274.96146.

Data for 5-(bromomethyl)-3-(2,4-dichlorophenyl)-1,2,4-oxadiazole (**A19**), yellow solid, m.p. 46.3–47.7 °C, yield, 60.1%; ^1H NMR (400 MHz, $CDCl_3$) δ 7.91 (d, *J* = 8.4 Hz, 1H, Ar-H), 7.57 (d, *J* = 2.0 Hz, 1H, Ar-H), 7.39 (dd, *J* = 8.4, 2.0 Hz, 1H, Ar-H), 4.58 (s, 2H, -CH_2Br). ^{13}C NMR (101 MHz, $CDCl_3$) δ 174.33, 167.07, 137.68, 134.34, 132.55, 130.99, 127.46, 124.04, 16.22. HRMS (ESI) m/z for $C_9H_5BrCl_2N_2O$ $[M+H]^+$ calcd: 306.90351, found: 306.90237.

Data for 5-(chloromethyl)-3-(2-chlorophenyl)-1,2,4-oxadiazole (**A20**), yellow oil, yield, 71.5 %; ^1H NMR NMR (500 MHz, $CDCl_3$) δ 7.92 (dd, *J* = 8.0, 2.0 Hz, 1H, Ar-H), 7.54 (dd, *J* = 8.0, 1.0 Hz, 1H, Ar-H), 7.45 (td, *J* = 7.5, 2.0 Hz, 1H, Ar-H), 7.39 (td, *J* = 7.5, 1.0 Hz, 1H, Ar-H), 4.78 (s, 2H, -CH_2Cl). ^{13}C NMR (101 MHz, $CDCl_3$) δ 173.92, 167.68, 132.05, 131.77, 131.03, 126.99, 125.44, 33.32. HRMS (ESI) m/z for $C_9H_6Cl_2N_2O$ $[M+H]^+$ calcd: 228.99299, found: 228.99297.

Data for 5-(chloromethyl)-3-(2,6-dichloropyridin-3-yl)-1,2,4-oxadiazole (**A21**), white solid, m.p. 41.6–42.3 °C, yield, 79.3%; ^1H NMR (500 MHz, $CDCl_3$) δ 8.26 (d, *J* = 8.5 Hz, 1H, Ar-H), 7.42 (d, *J* = 8.5 Hz, 1H, Ar-H), 4.77 (s, 2H, -CH_2Cl). ^{13}C NMR (101 MHz, $CDCl_3$) δ 174.64, 165.76, 152.46, 149.15, 142.28, 123.23, 121.40, 33.15. HRMS (ESI) m/z for $C_8H_4Cl_3N_3O$ $[M+H]^+$ calcd: 263.94927, found: 263.94839.

Data for 5-(bromomethyl)-3-(2-bromophenyl)-1,2,4-oxadiazole (**A22**), white solid, m.p. 53.7-55.0 °C, yield, 58.7%; ^1H NMR (400 MHz, $CDCl_3$) δ 7.84 (dd, *J* = 7.6, 1.6Hz, 1H, Ar-H), 7.74 (dd, J = 8.0, 1.2 Hz, 1H, Ar-H), 7.44 (td, *J* = 7.2, 1.2 Hz, 1H, Ar-H), 7.37 (td, *J* = 8.0, 2.0 Hz, 1H, Ar-H), 4.59 (s, 2H, -CH_2Br). ^{13}C NMR (101 MHz, $CDCl_3$) δ 173.26, 168.63, 162.43, 129.29, 129.29, 118.07, 118.07, 114.42 , 58.28, 55.44 HRMS (ESI) m/z for $C_9H_6Br_2N_2O$ $[M+H]^+$ calcd: 316.89196, found: 316.89084.

Data for 3-(2-chloro-5-fluorophenyl)-5-(chloromethyl)-1,2,4-oxadiazole (**A23**), yellow solid, m.p. 31.8–32.1 °C, yield, 76.90%; ^1H NMR (400 MHz, $CDCl_3$) δ 7.68 (dd, *J* = 8.8, 3.2 Hz, 1H, Ar-H), 7.50 (dd, *J* = 8.8, 4.8 Hz, 1H, Ar-H), 7.16 (ddd, J = 9.2, 7.2, 2.8 Hz, 1H, Ar-H), 4.77 (s, 2H, -CH_2Cl). ^{13}C NMR (101 MHz, $CDCl_3$) δ 174.77, 163.15, 161.19 (d, *J* = 256.54 Hz), 135.31 (d, *J* = 3.0 Hz), 132.77 (d, *J* = 3.0 Hz), 125.90 (d, *J* = 3.0 Hz), 115.34 (d, *J* = 17.2 Hz), 114.65 (d, *J* = 21.2 Hz), 33.38 (s). HRMS (ESI) m/z for $C_9H_5Cl_2FN_2O$ $[M-H]^-$ calcd: 244.96792, found: 244.96907.

Data for 3-(2-bromophenyl)-5-(chloromethyl)-1,2,4-oxadiazole (**A24**), white solid, m.p. 47.8–47.9 °C, yield, 60.1%; ^1H NMR (400 MHz, CDCl3) δ 7.81 (dd, *J* = 7.6, 1.6 Hz, 1H, Ar-H), 7.73 (dd, *J* = 8.0, 1.2 Hz, 1H, Ar-H), 7.43 (td, *J* = 7.6, 1.2 Hz, 1H, Ar-H), 7.35 (td, *J* = 7.6, 1.6 Hz, 1H, Ar-H), 4.78 (s, 2H, -CH_2Cl). ^{13}C NMR (101 MHz, $CDCl_3$) δ 173.99, 168.45, 134.31, 133.90, 133.21, 132.00, 127.53, 122.12, 33.36. HRMS (ESI) m/z for $C_9H_6BrClN_2O$ $[M+H]^+$ calcd: 272.94248, found: 272.94241.

Data for 5-(2-chloroethyl)-3-(4-chlorophenyl)-1,2,4-oxadiazole (**A25**), white solid, m.p. 61.2–63.2 °C, yield, 51.2%; ^1H NMR (400 MHz, $CDCl_3$) δ 8.05–8.00 (m, 2H, Ar-H), 7.49–7.44 (m, 2H, Ar-H), 3.99 (t, *J* = 6.8 Hz, 2H，-CH_2-), 3.43 (t, *J* = 6.8 Hz, 2H, -CH_2Cl). ^{13}C NMR (101 MHz, $CDCl_3$) δ 176.49, 167.72, 137.48, 129.24, 129.24,128.79, 128.79, 125.07, 39.42, 30.32. HRMS (ESI) m/z for $C_{10}H_8Cl_2N_2O$ $[M+H]^+$ calcd: 243.00864, found: 243.00871.

Data for 5-(chloromethyl)-3-(2,4,5-trifluorophenyl)-1,2,4-oxadiazole (**A26**), white solid, m.p. 43.7–44.1 °C, yield, 73.1%, ^1H NMR (400 MHz, $CDCl_3$) δ 7.90 (ddd, *J* = 10, 8.4, 6.4 Hz, 1H, Ar-H), 7.12 (td, *J* = 9.6, 6.4 Hz, 1H, Ar-H), 4.76 (s, 2H, -CH_2Cl). ^{13}C NMR (101 MHz, $CDCl_3$) δ 174.44, 164.41 (d, *J* = 6.2 Hz), 156.32 (ddd, *J* = 258.5, 9.9, 2.7 Hz), 152.09 (ddd, *J* = 257.9, 14.4, 11.8 Hz), 146.99 (ddd, *J* = 246.6, 12.8, 3.8 Hz), 118.47 (ddd, *J* = 21.4, 3.6, 1.8 Hz), 111.20–110.85 (m), 107.11 (dd, *J* = 27.0, 21.2 Hz), 33.10. HRMS (ESI) m/z for $C_9H_4ClF_3N_2O$ $[M+H]^+$ calcd: 249.00370, found: 249.00259.

Data for 5-(chloromethyl)-3-(2,6-difluorophenyl)-1,2,4-oxadiazoleyield (**A27**), white solid, m.p. 59.8–61.2 °C, yield, 70.4%; ^1H NMR (400 MHz, $CDCl_3$) δ 7.48 (tt, *J* = 8.5, 6.2 Hz, 1H, Ar-H), 7.05 (t, *J* = 8 Hz, 2H, Ar-H), 4.79 (s, 2H, -CH_2Cl). ^{13}C NMR (101 MHz, $CDCl_3$) δ 174.61, 162.19 (d, *J* = 5.8 Hz), 161.51 (t, *J* = 2.8

Hz), 159.63 (d, J = 5.7 Hz), 133.11 (t, J = 10.5 Hz), 112.32 – 112.30 (m), 112.12 – 112.07 (m), 104.97 (t, J = 17.1 Hz), 33.27. HRMS (ESI) m/z for $C_9H_5ClF_2N_2O$ $[M+H]^+$ calcd: 231.01312, found: 231.01308.

Data for 5-(chloromethyl)-3-(4-fluoro-2-methylphenyl)-1,2,4-oxadiazole (**A28**), white solid, m.p. 33.2–33.9 °C, yield, 70.4%; ^{1}H NMR (400 MHz, $CDCl_3$) δ 8.00 (dd, J = 8.4, 6.0 Hz, 1H, Ar-H), 7.04–6.99 (m, 3H, Ar-H), 4.75 (s, 2H, $-CH_2Cl$), 2.63 (s, 3H, Ar-CH_3). ^{13}C NMR (101 MHz, $CDCl_3$) δ 173.36 (s), 168.70 (s), 164.00 (d, J = 252.5 Hz), 141.45 (d, J = 9.0 Hz), 132.34 (d, J = 10.1z Hz), 121.66 (d, J = 3.0 Hz), 118.30 (d, J = 21.2 Hz), 113.21 (d, J = 21.2 Hz), 33.31, 22.33. HRMS (ESI) m/z for $C_{10}H_8ClFN_2O$ $[M+H]^+$ calcd: 227.03820, found: 227.03806.

Data for 5-(chloromethyl)-3-(3,4-difluorophenyl)-1,2,4-oxadiazole (**A29**), colorless oil, yield, 77.8%; ^{1}H NMR (400 MHz, $CDCl_3$) δ 7.86–7.74 (m, 2H, Ar-H), 7.20 (dt, J = 11.2, 8.0 Hz, 1H, Ar-H), 4.67 (s, 2H, $-CH_2Cl$). ^{13}C NMR (101 MHz, $CDCl_3$) δ 174.49, 165.26 (dd, J = 5.1, 1.0 Hz), 164.92 (dd, J = 205.03，9.09 Hz), 162.40 (d, J = 12.1 Hz), 160.32 (d, J =9.1 Hz), 132.11 (dd, J = 8.1, 3.0 Hz), 112.28 (dd, J = 17.2, 3.0 Hz), 111.14 (dd, J = 9.1, 3.0 Hz), 105.61-105.20 (m), 16.27. HRMS (ESI) m/z for $C_9H_5ClF_2N_2O$ $[M+H]^+$ calcd: 231.01312, found: 231.01292.

Data for 5-(chloromethyl)-3-(p-tolyl)-1,2,4-oxadiaz (**B1**), White solid, m.p. 42.0–42.6 °C, yield, 64.3%; ^{1}H NMR (400 MHz, $CDCl_3$) δ 7.97 (d, J = 8 Hz, 2H, Ar-H), 7.30 (d, J = 8 Hz, 2H, Ar-H), 4.74 (s, 2H, $-CH_2Cl$), 2.42 (s, 3H, Ar-CH_3). ^{13}C NMR (101 MHz, $CDCl_3$) δ 174.14, 168.88, 141.97, 129.67, 129.67, 127.34, 123.32, 33.40, 21.60. HRMS (ESI) m/z for $C_{10}H_9ClN_2O$ $[M+H]^+$ calcd:209.04762, found: 209.04762.

Data for 5-(chloromethyl)-3-(thiophen-2-yl)-1,2,4-oxadiazole (**B2**), white solid, m.p. 57.1–57.8 °C, yield, 53.7%; ^{1}H NMR (400 MHz, $CDCl_3$) δ 7.81 (dd, J = 3.6, 1.2 Hz, 1H, Thiophene-H), 7.53 (dd, J = 4.8, 0.8 Hz, 1H, thiophene-H), 7.16 (dd, J = 5.2, 4.0 Hz, 1H, thiophene-H), 4.72 (s, 2H, $-CH_2Cl$). ^{13}C NMR (101 MHz, $CDCl_3$) δ 174.27, 164.92, 130.08, 129.80, 128.12, 127.54, 33.22. HRMS (ESI) m/z for $C_7H_5ClN_2OS$ $[M+H]^+$ calcd: 200.98839, found: 200.98839.

Data for 5-(bromomethyl)-3-(furan-2-yl)-1,2,4-oxadiazole (**B3**), yellow solid, m.p. 81.9–83.0 °C, yield, 58.4%; ^{1}H NMR (400 MHz, $CDCl_3$) δ 7.64–7.60 (m, 1H, furan-H), 7.16 (dd, J = 6.0, 5.2 Hz, 1H, furan-H), 6.57 (tt, J = 4.6, 1.9 Hz, 1H, furan-H), 4.53 (d, J = 2.0 Hz, 2H, $-CH_2Br$). ^{13}C NMR (101 MHz, $CDCl_3$) δ 174.67, 161.74, 145.54, 141.62, 114.38, 111.98, 16.15. HRMS (ESI) m/z for $C_7H_5BrN_2O_2$ $[M+H]^+$ calcd: 228.96072, found: 228.96060.

Data for 5-(chloromethyl)-3-(4-methoxyphenyl)-1,2,4-oxadiazole (**B4**), white solid, m.p. 43.7–45.1 °C, yield, 59.4%; ^{1}H NMR (400 MHz, CDCl3) δ 8.01 (d, J = 9.2 Hz, 2H, Ar-H), 6.98 (d, J = 8.8 Hz, 2H, Ar-H), 4.72 (s, 2H), 3.86 (s, 3H, $-OCH_3$). ^{13}C NMR (101 MHz, $CDCl_3$) δ 174.01 168.56, 162.19, 133.99, 129.14, 118.56, 55.47, 33.41. HRMS (ESI) m/z for $C_{10}H_9ClN_2O_2$ $[M+H]^+$ calcd: 225.04253, found: 225.04242.

Data for 5-(chloromethyl)-3-(furan-2-yl)-1,2,4-oxadiazole (**B5**), white solid, m.p. 79.8–81.22 °C, yield 65.5%; ^{1}H NMR (400 MHz, $CDCl_3$) δ 7.56 (d, J = 0.8 Hz, 1H, furan-H), 7.10 (d, J = 3.2 Hz, 1H, furan-H), 6.51 (dd, J = 3.6, 2.0 Hz, 1H, furan-H), 4.66 (s, 2H, $-CH_2Cl$). ^{13}C NMR (101 MHz, $CDCl_3$) δ 174.40, 161.67, 145.58, 141.58, 114.44, 111.99, 33.16. HRMS (ESI) m/z for $C_7H_5ClN_2O_2$ $[M+H]^+$ calcd: 185.01123, found: 185.01120.

Data for 5-(bromomethyl)-3-(thiophen-2-yl)-1,2,4-oxadiazole (**B6**), yellow solid, m.p. 37.7–38.9 °C, yield 67.8%; ^{1}H NMR (400 MHz, $CDCl_3$) δ 7.76–7.71 (m, 1H, thiophene-H), 7.48–7.43 (m, 1H, thiophene-H), 7.12–7.05 (m, 1H, thiophene-H), 4.46 (d, J = 1.2 Hz, 2H, $-CH_2Br$). ^{13}C NMR (101 MHz, $CDCl_3$) δ 174.55, 165.01, 130.03, 129.76, 128.11, 127.61, 16.22. HRMS (ESI) m/z for $C_7H_5BrN_2OS$ $[M+H]^+$ calcd: 244.93787, found: 244.93774.

Data for 3-(4-(tert-butyl)phenyl)-5-(chloromethyl)-1,2,4-oxadiazole (**B7**), white solid, m.p. 47.8–48.1 °C, yield, 79.61%; ^{1}H NMR (400 MHz, $CDCl_3$) δ 8.01 (d, J = 8.0 Hz, 2H, Ar-H), 7.51 (d, J = 8.0 Hz, 2H, Ar-H),

4.74 (s, 2H, -CH_2Cl), 1.36 (s, 9H, -$C(CH_3)_3$). ^{13}C NMR (101 MHz, $CDCl_3$) δ 174.15, 168.82, 155.06, 127.30, 127.30, 125.94, 125.94, 123.29, 35.03, 33.41, 31.17, 31.17, 31.17. HRMS (ESI) m/z for $C_{13}H_{15}ClN_2O$ $[M+H]^+$ calcd: 251.09457, found: 251.09438.

Data for 5-(chloromethyl)-3-phenyl-1,2,4-oxadiazole (**B8**), white solid, m.p. 35.8–36.1 °C; yield, 60.7%; ^1H NMR (400 MHz, $CDCl_3$) δ 8.11–8.07 (m, 2H, Ar-H), 7.56–7.47 (m, 3H, Ar-H), 4.75 (s, 2H, -CH_2Cl). ^{13}C NMR (101 MHz, $CDCl_3$) δ 174.32, 168.89, 131.56, 128.96, 128.96, 127.51, 127.51, 126.16, 33.37. HRMS (ESI) m/z for $C_9H_7ClN_2O$ $[M+H]^+$ calcd: 195.03197, found: 195.03200.

Data for 5-(bromomethyl)-3-phenyl-1,2,4-oxadiazole (**B9**), white solid, m.p. 52.2–52.9 °C, yield, 83.4%; ^1H NMR (400 MHz, $CDCl_3$) δ 8.08 (dd, *J* = 7.6，1.2 Hz, 1H, Ar-H), 7.528-7.466 (m, 3H, Ar-H), 4.56 (s, 2H, -CH_2Br). ^{13}C NMR (101 MHz, $CDCl_3$) δ 174.59, 168.97, 131.54, 128.96, 128.96, 127.50, 127.50, 126.20, 16.49. HRMS (ESI) m/z for $C_9H_7BrN_2O$ $[M+H]^+$ calcd: 238.98145, found: 238.98123.

Data for 5-(chloromethyl)-3-(6-methylpyridin-3-yl)-1,2,4-oxadiazole (**B10**), yellow solid, m.p. 72.0–72.6 °C, yield, 65.8 %; ^1H NMR (500 MHz, $CDCl_3$) δ 9.13 (d, *J* = 2.0 Hz, 1H, Ar-H), 8.18 (dd, J = 8.5, 2.0 Hz, 1H, Ar-H), 7.24 (d, *J* = 8.1 Hz, 1H, Ar-H), 4.73 (s), 2.59 (s, 2H, -CH_2Cl). ^{13}C NMR (101 MHz, $CDCl_3$) δ 174.65, 167.01, 161.79, 147.93, 134.97, 134.68, 123.62, 123.37, 119.64, 33.26, 24.59. HRMS (ESI) m/z for $C_9H_8ClN_3O$ $[M+H]^+$ calcd: 210.04287, found: 210.04271.

Data for 5-(bromomethyl)-3-(4-methoxyphenyl)-1,2,4-oxadiazole (**B11**), yellow solid, m.p. 35.7–37.8 °C, yield, 68.8%; ^1H NMR (400 MHz, $CDCl_3$) δ 8.05–7.92 (m, 2H, Ar-H), 7.05–6.89 (m, 2H, Ar-H), 4.54 (s, 2H, -CH_2Br), 3.87 (s, 3H, Ar-OCH_3). ^{13}C NMR (101 MHz, $CDCl_3$) δ 174.28, 168.64, 162.17, 128.82, 128.82, 118.60, 114.35, 114.35, 55.48, 16.58. HRMS (ESI) m/z for $C_{10}H_9BrN_2O_2$ $[M+H]^+$ calcd: 268.99202, found: 268.99182.

Data for 5-(bromomethyl)-3-(3-methoxyphenyl)-1,2,4-oxadiazole (**B12**), yellow solid, m.p. 58.7–61.2 °C, yield, 59.5 %; ^1H NMR (400 MHz, $CDCl_3$) δ 7.67–7.65 (m, 1H, Ar-H), 7.59 (dd, *J* = 2.0, 1.2 Hz, 1H, Ar-H), 7.39 (d, *J* = 6.4 Hz,1H, Ar-H), 7.07–7.04 (m, 1H, Ar-H), 4.55 (s, 2H, -CH_2Br), 3.86 (s, 3H, Ar-OCH_3). ^{13}C NMR (101 MHz, $CDCl_3$) δ 174.65 (s), 168.97 (s), 159.98 (s), 130.17 (s), 127.40 (s), 120.01 (s), 118.16 (s), 111.99 (s), 55.55 (s), 16.56 (s). HRMS (ESI) m/z for $C_{10}H_9BrN_2O_2$ $[M+H]^+$ calcd: 268.99202, found: 268.99185.

Data for 5-(bromomethyl)-3-(3,4-dimethylphenyl)-1,2,4-oxadiazole (**B13**), white solid, m.p. 73.9–74.5 °C, yield, 51.6%; ^1H NMR (400 MHz, $CDCl_3$) δ7.84 (s, 1H, Ar-H), 7.80 (d, *J* = 6.4 Hz, 1H, Ar-H), 7.24 (d, *J* = 6.4 Hz, 1H, Ar-H),4.67 (s, 2H, -CH_2Br), 4.54 (s, 2H), 2.32 (s, 6H, –$(CH_3)_2$). ^{13}C NMR (101 MHz, $CDCl_3$) δ 174.42, 169.11, 140.76, 137.49, 130.30, 128.52, 125.03, 123.66, 20.06, 19.83, 16.64. HRMS (ESI) m/z for $C_{11}H_{11}BrN_2O$ $[M+H]^+$ calcd: 267.01275, found: 267.01273.

Data for 5-(bromomethyl)-3-(o-tolyl)-1,2,4-oxadiazole (**B14**), colorless oil, yield, 64.8%; ^1H NMR (400 MHz, $CDCl_3$) δ 7.99 (d, *J* = 6.4, 1H, Ar-H), 7.40-7.38 (m, 1H, Ar-H), 7.32 (s, 2H, Ar-H), 4.56 (s, 2H, -CH_2Br), 2.63 (s, 3H, Ar-CH_3). ^{13}C NMR (101 MHz, $CDCl_3$) δ 174.59, 169.13, 138.88, 132.43, 128.96, 128.11, 126.07, 124.67, 21.45, 16.59. HRMS (ESI) m/z for $C_{10}H_9BrN_2O$ $[M+H]^+$calcd: 252.99710, found: 252.99709.

Data for 5-(chloromethyl)-3-(o-tolyl)-1,2,4-oxadiazole (**B15**), white solid, m.p. 54.0–54.7 °C, yield, 60.7%; ^1H NMR (400 MHz, $CDCl_3$) δ 7.99 (d, *J* = 7.6 Hz, 1H, Ar-H), 7.43–7.38 (m, 1H, Ar-H), 7.35–7.29 (m, 2H, Ar-H), 4.76 (s, 2H, -CH_2Cl), 2.64 (s, 3H, -CH_3). ^{13}C NMR (101 MHz, $CDCl_3$) δ 173.29, 169.43, 138.37, 131.48, 130.92, 130.14, 126.07, 125.42, 33.38, 22.18. HRMS (ESI) m/z for $C_{10}H_9ClN_2O$ $[M+H]^+$ calcd: 209.04762, found: 209.04761.

Data for 5-(dichloromethyl)-3-(p-tolyl)-1,2,4-oxadiazole (**B16**), white solid, m.p. 20.5–22.4 °C, yield, 57.0%; ^1H NMR (400 MHz, $CDCl_3$) δ 7.98 (d, *J* = 6.8 Hz, 2H, Ar-H), 7.30 (d, *J* = 6.4 Hz, 2H, Ar-H), 6.85 (s, 1H, -$CHCl_2$), 2.42 (s, 3H, Ar-CH_3). ^{13}C NMR (101 MHz, $CDCl_3$) δ 173.46, 169.00, 142.42, 129.81, 129.81,

127.60, 122.90, 122.90, 58.34, 21.74. HRMS (ESI) m/z for $C_{10}H_8Cl_2N_2O$ $[M+H]^+$ calcd: 243.00864, found: 243.00871.

Data for 5-(bromomethyl)-3-(2-methoxyphenyl)-1,2,4-oxadiazole (**B17**), yellow oil, yield, 59.8%; ^1H NMR (400 MHz, $CDCl_3$) δ 7.97 (dd, J = 7.6, 1.6 Hz, 1H, Ar-H), 7.50–7.46k (m, 1H, Ar-H), 7.09-7.04 (m, 2H, Ar-H), 4.55 (s, 2H, $-CH_2Br$), 3.97 (s, 3H, $Ar-CH_3$). ^{13}C NMR (101 MHz, $CDCl_3$) δ 173.45, 167.45, 158.14, 132.69, 131.35, 120.73, 115.19, 111.72, 56.02, 16.52. HRMS (ESI) m/z for $C_{10}H_9BrN_2O_2$ $[M+H]^+$calcd: 268.99202, found: 268.99188.

Data for 3-(p-tolyl)-5-(trichloromethyl)-1,2,4-oxadiazole (**B18**), white solid, m.p. 66.4–66.9 °C, yield, 51.5%; ^1H NMR (400 MHz, $CDCl_3$) δ 8.0 (d, *J* = 6.4 Hz, 2H, Ar-H), 7.32 (d, *J* = 6.4 Hz, 2H, Ar-H), 2.44 (s, 3H, $Ar-CH_3$). ^{13}C NMR (101 MHz, $CDCl_3$) δ 174.24, 169.16, 142.55, 129.76, 129.76, 127.62, 127.62,122.63, 83.57, 21.68. HRMS (ESI) m/z for $C_{10}H_7Cl_3N_2O$ $[M+H]^+$ calcd: 276.96967, found: 276.96967.

Data for 5-(1-bromopropyl)-3-(p-tolyl)-1,2,4-oxadiazole (**B19**), colorless oil, yield, 78.4 %; ^1H NMR (400 MHz, $CDCl_3$) δ 7.95 (d, *J* = 8.0 Hz, 2H, Ar-H), 7.28 (d, *J* = 8.0 Hz, 2H, Ar-H), 3.71 (t, *J* = 6.0 Hz, 2H, $-C\underline{H}_2CH_2CH_3$), 3.14 (t, *J* = 7.2 Hz, 2H, $-CH_2C\underline{H}_2CH_3$), 2.41 (s, 3H, $Ar-CH_3$) 2.46–2.32 (m, 2H, $-CH_2CH_2C\underline{H}_3$). ^{13}C NMR (101 MHz, $CDCl_3$) δ 173.45, 167.45, 158.14, 132.69, 132.69, 131.35, 131.35, 120.74, 115.19, 111.72, 56.02, 16.54. HRMS (ESI) m/z for $C_{12}H_{13}BrN_2O$ $[M+H]^+$ calcd: 281.02840, found: 281.02820.

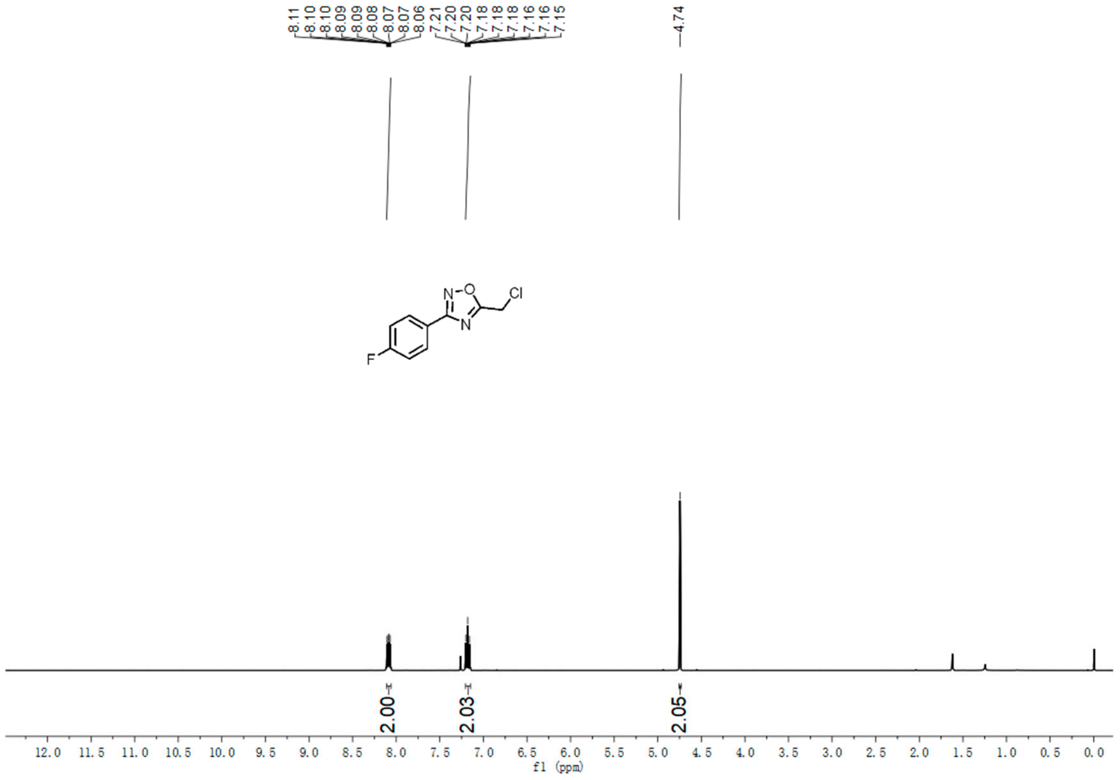

^1H NMR of compound **A1**

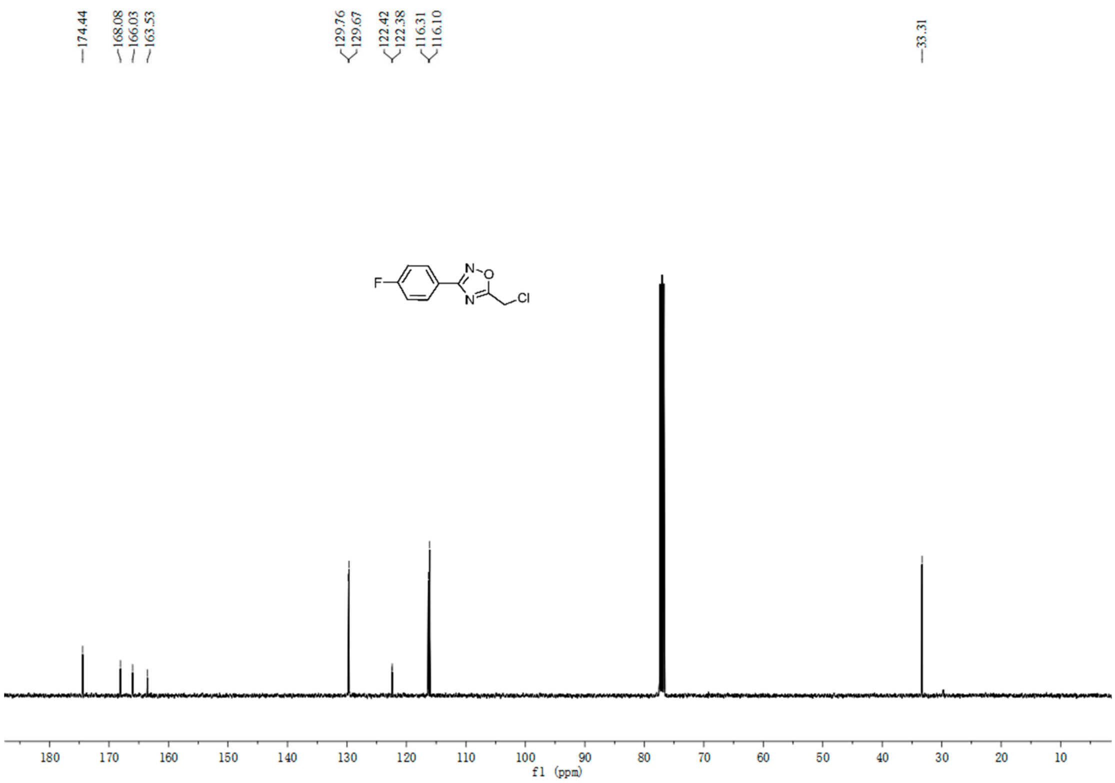

^{13}C NMR of compound **A1**

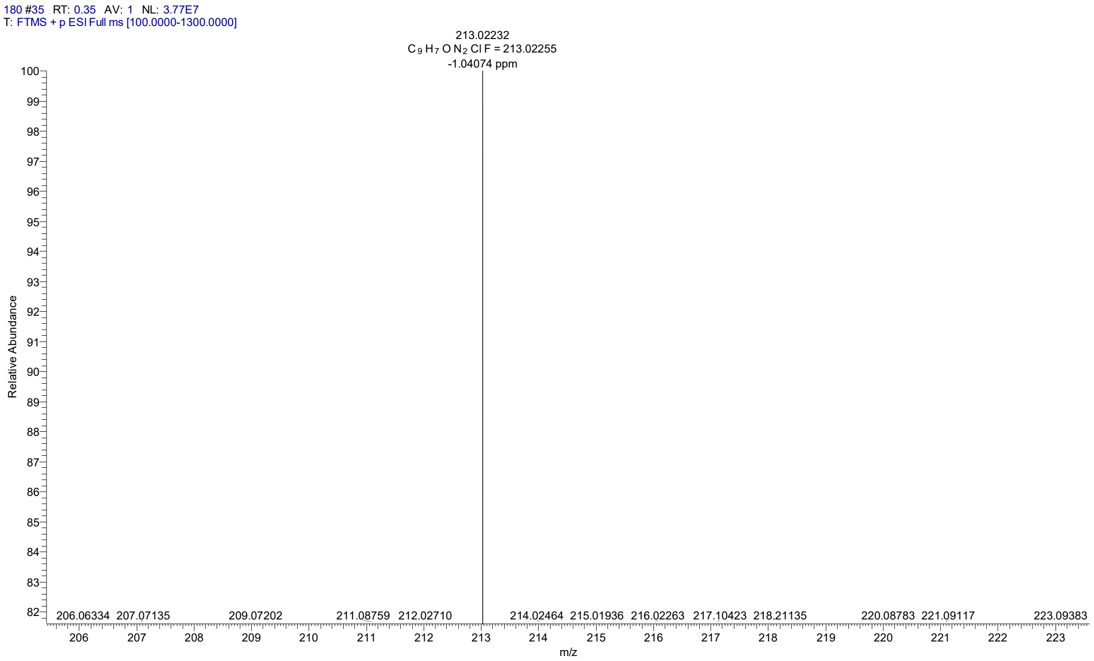

HRMS of compound **A1**

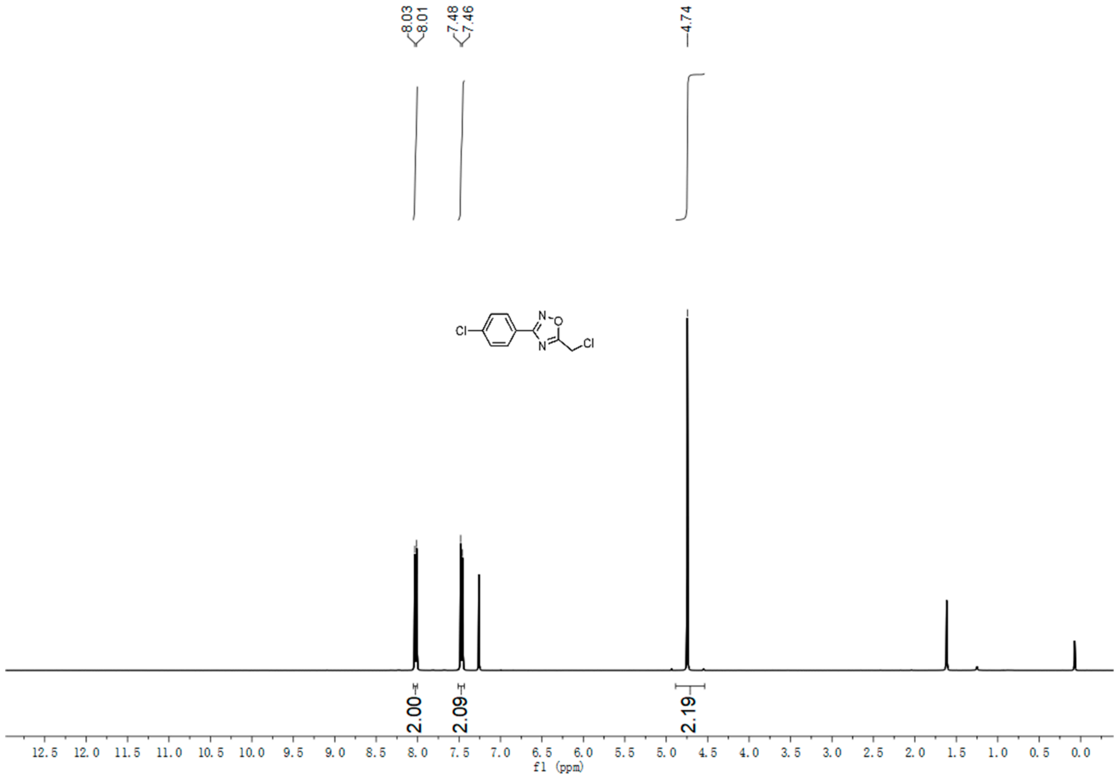

^{1}H NMR of compound **A2**

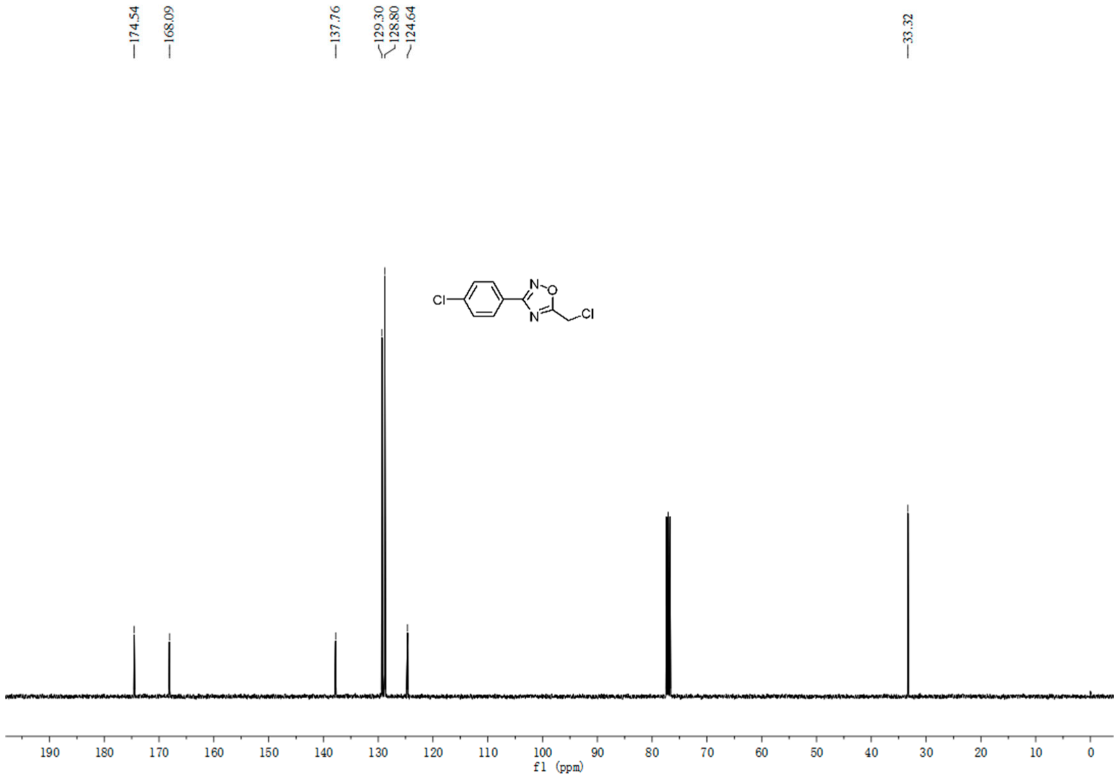

^{13}C NMR of compound **A2**

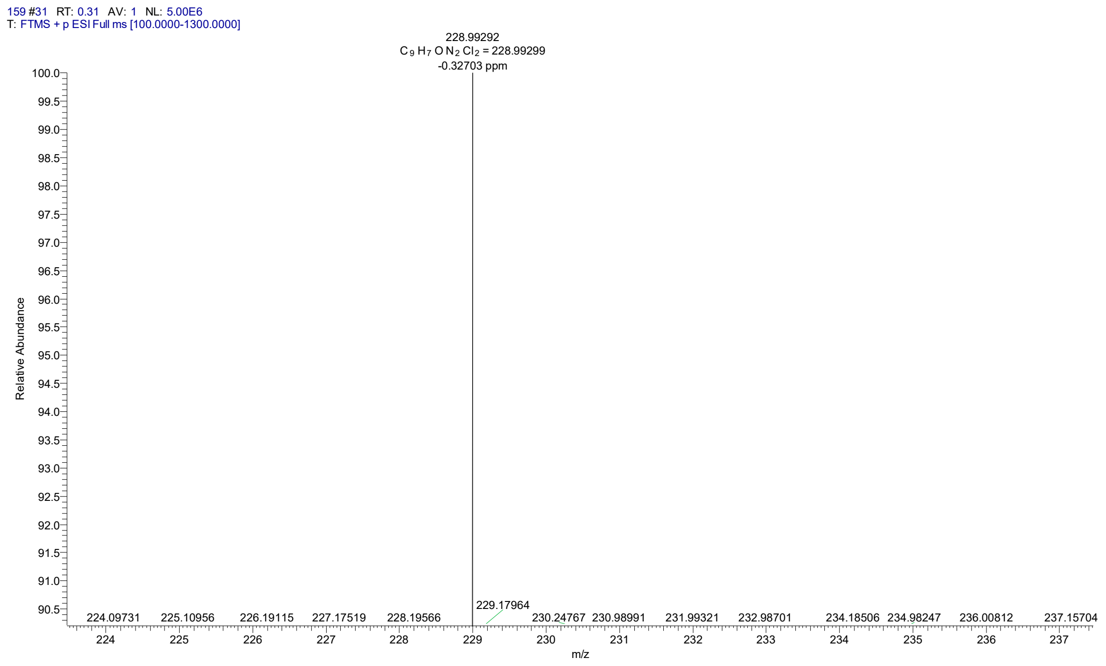

HRMS of compound **A2**

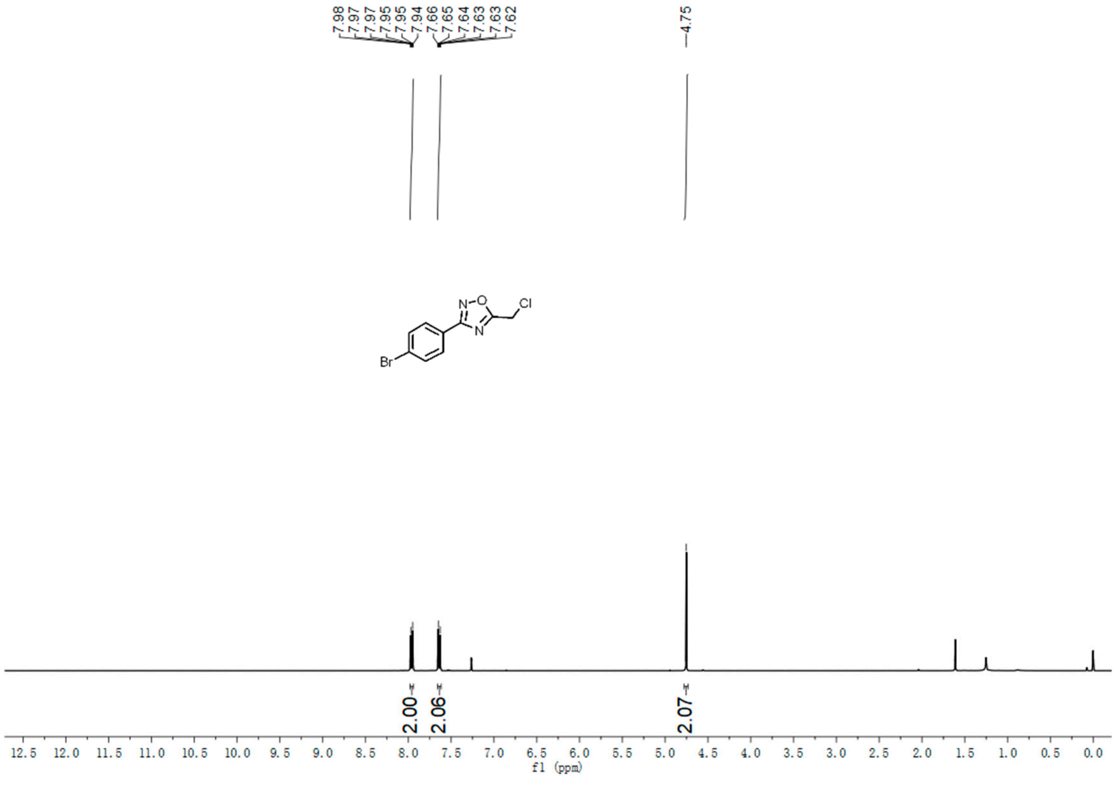

^{1}H NMR of compound **A3**

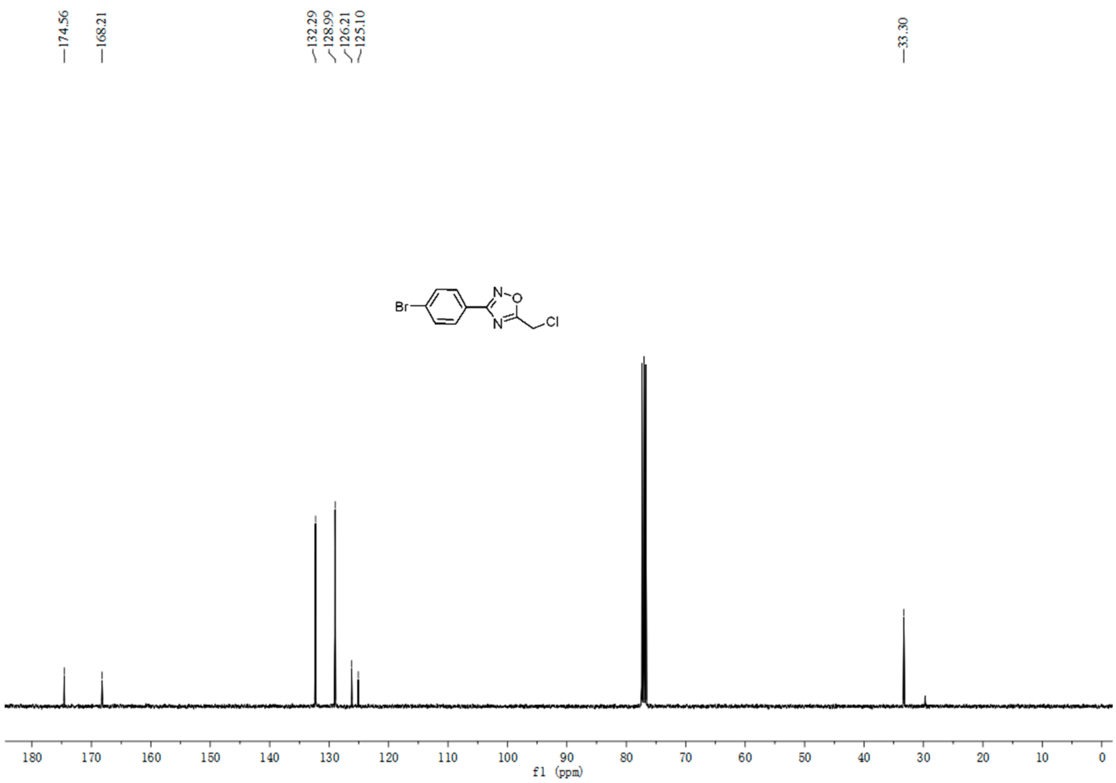

^{13}C NMR of compound **A3**

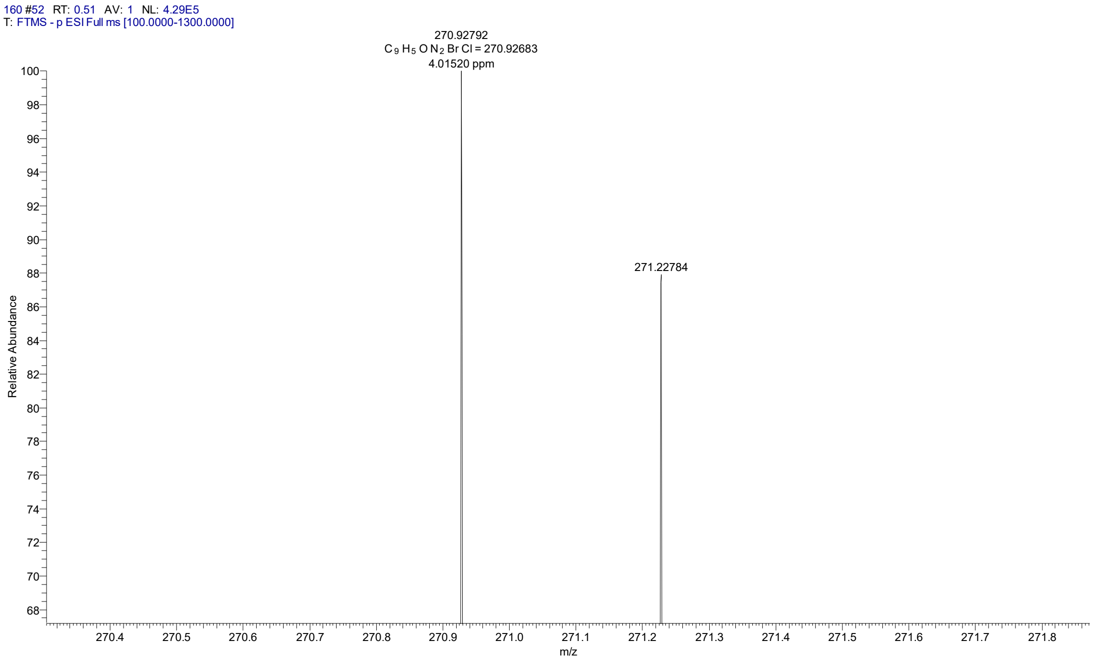

HRMS of compound **A3**

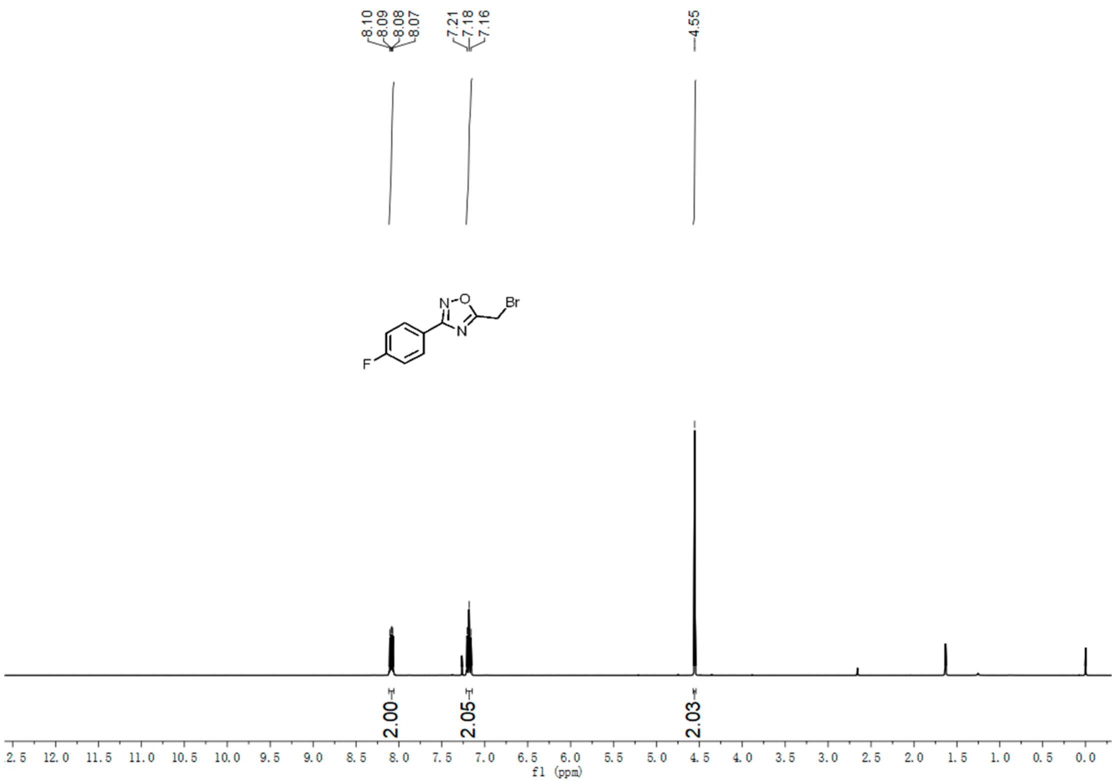

^{1}H NMR of compound **A4**

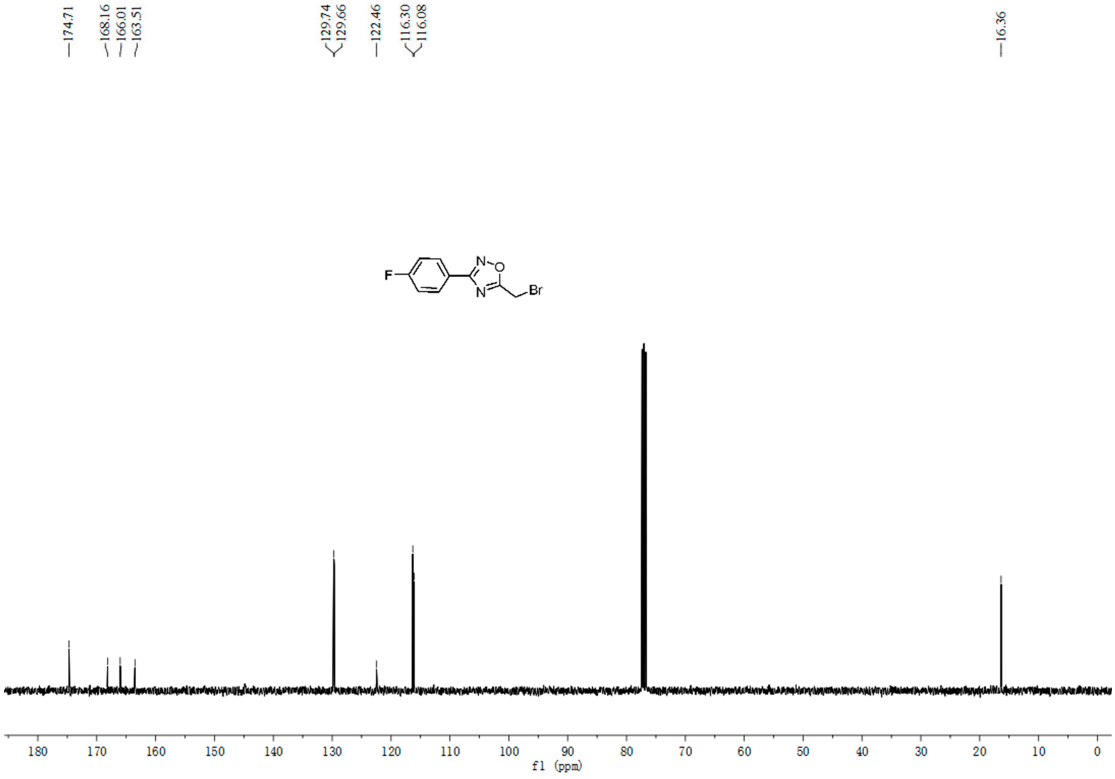

^{13}C NMR of compound **A4**

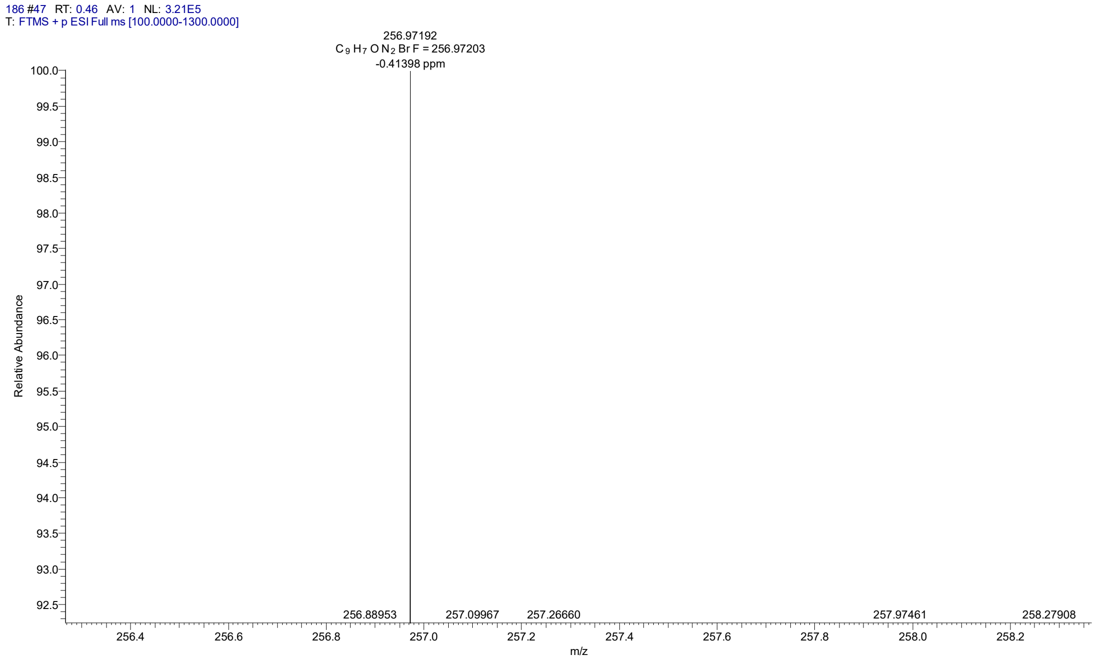

HRMS of compound **A4**

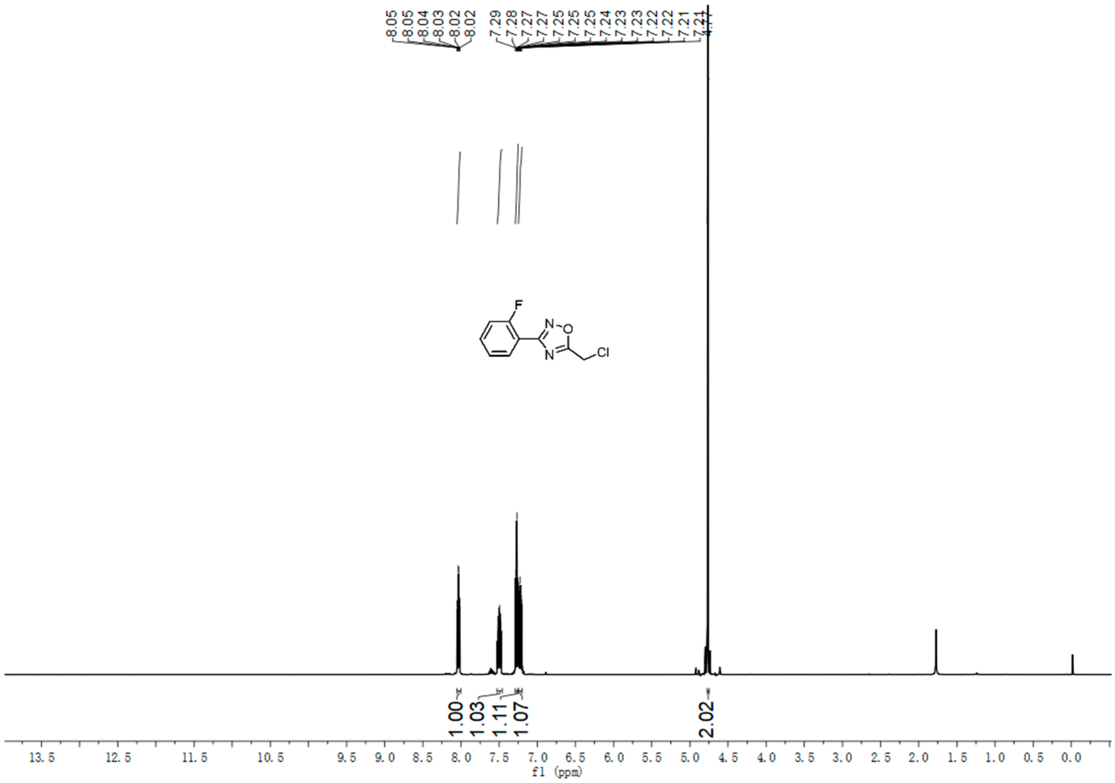

^{1}H NMR of compound **A5**

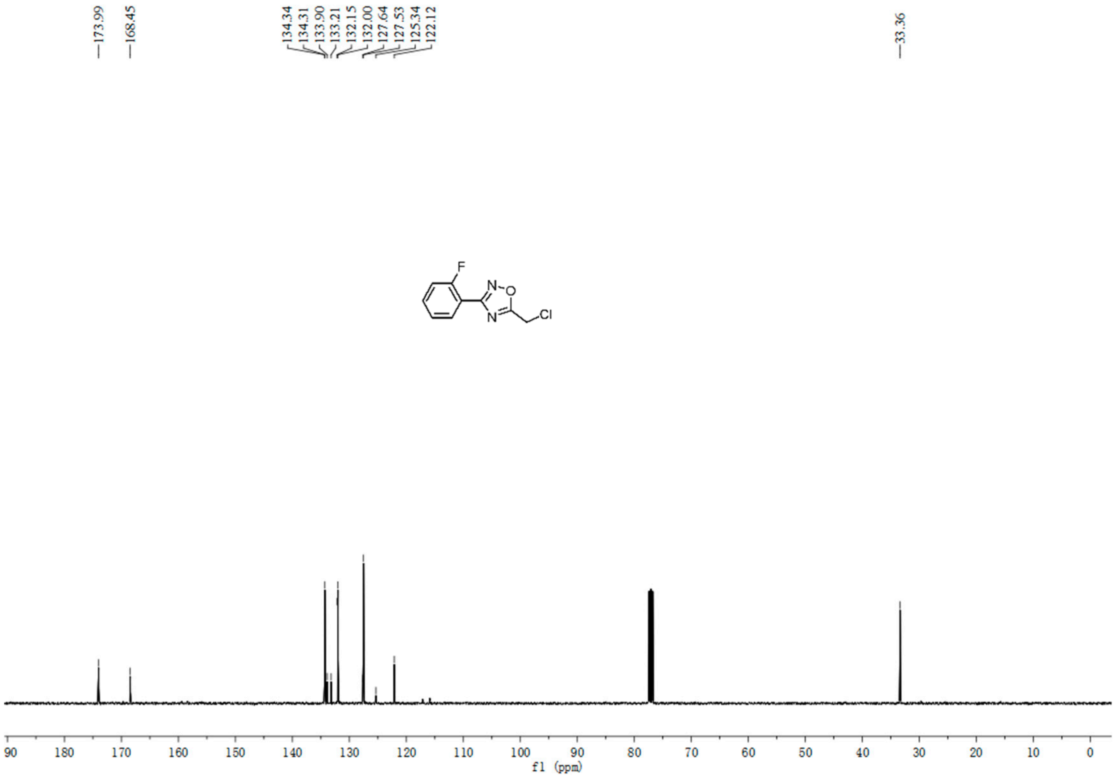

^{13}C NMR of compound **A5**

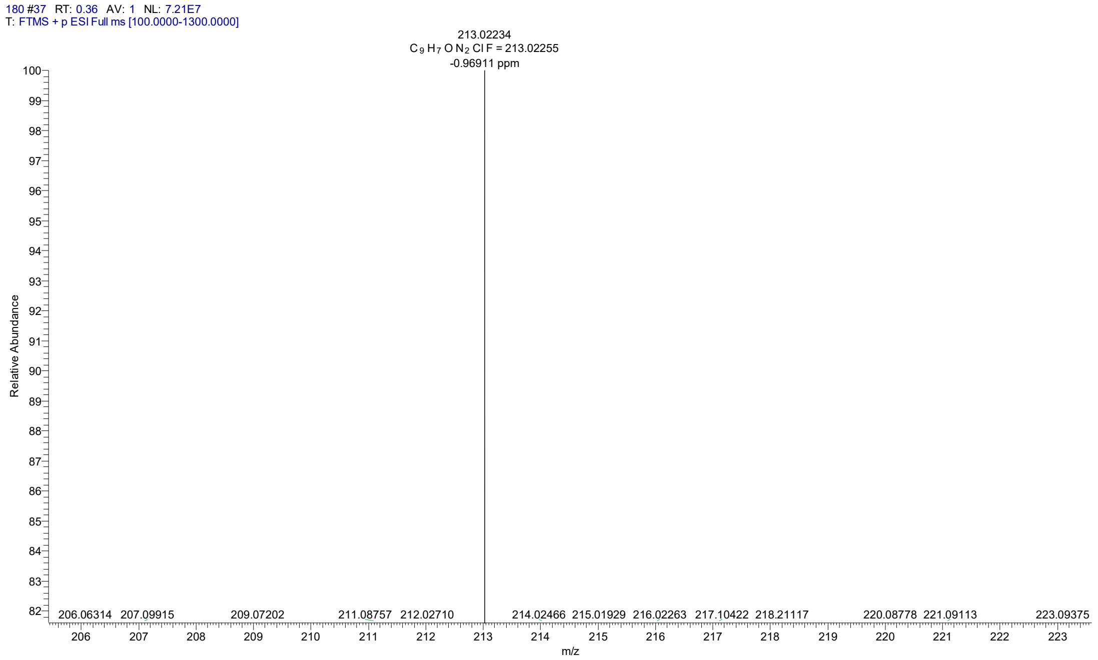

HRMS of compound **A5**

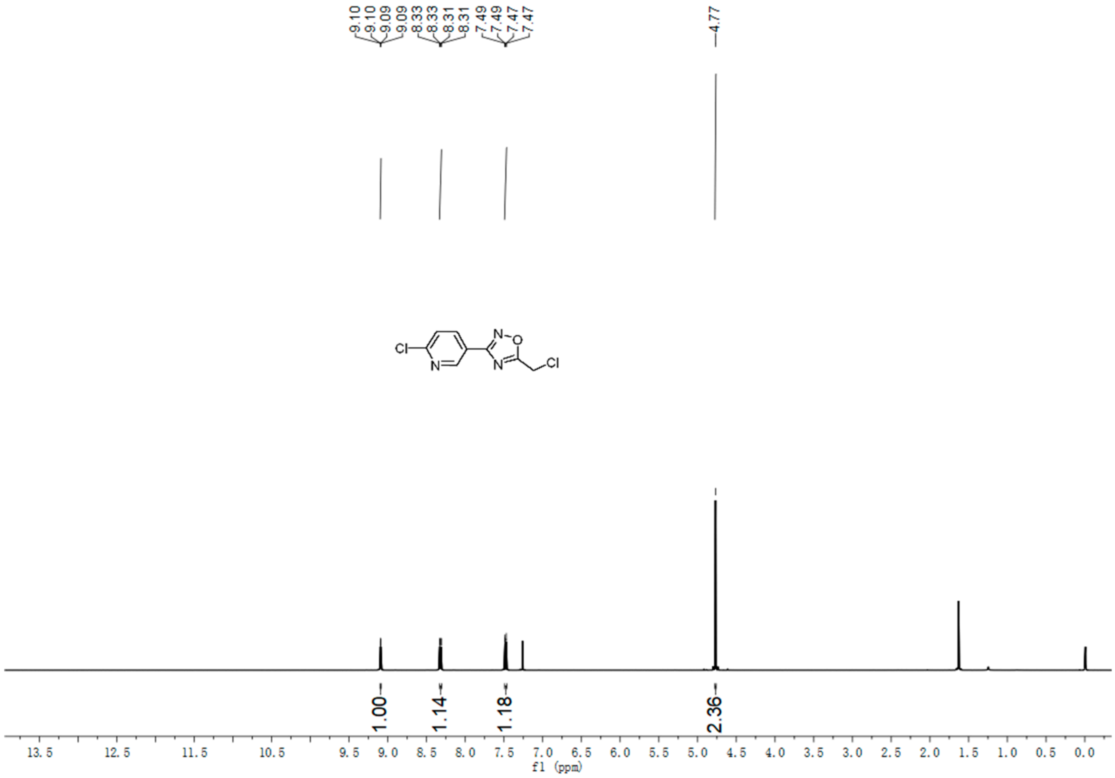

^{1}H NMR of compound **A6**

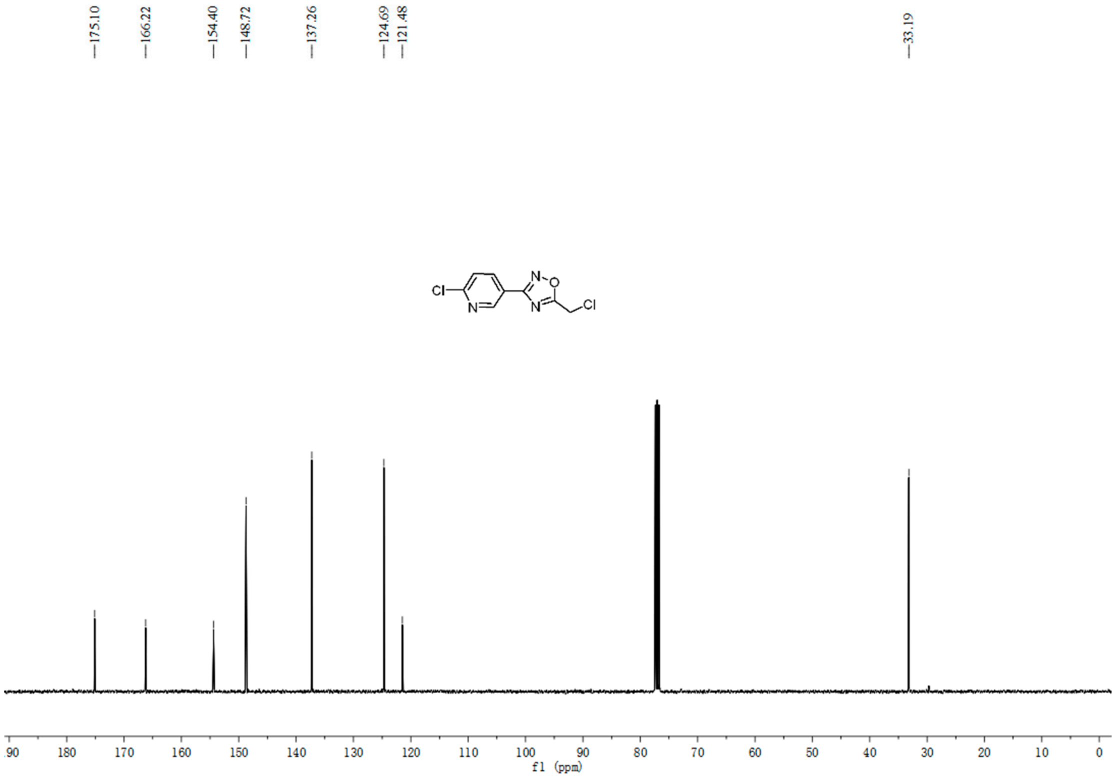

^{13}C NMR of compound **A6**

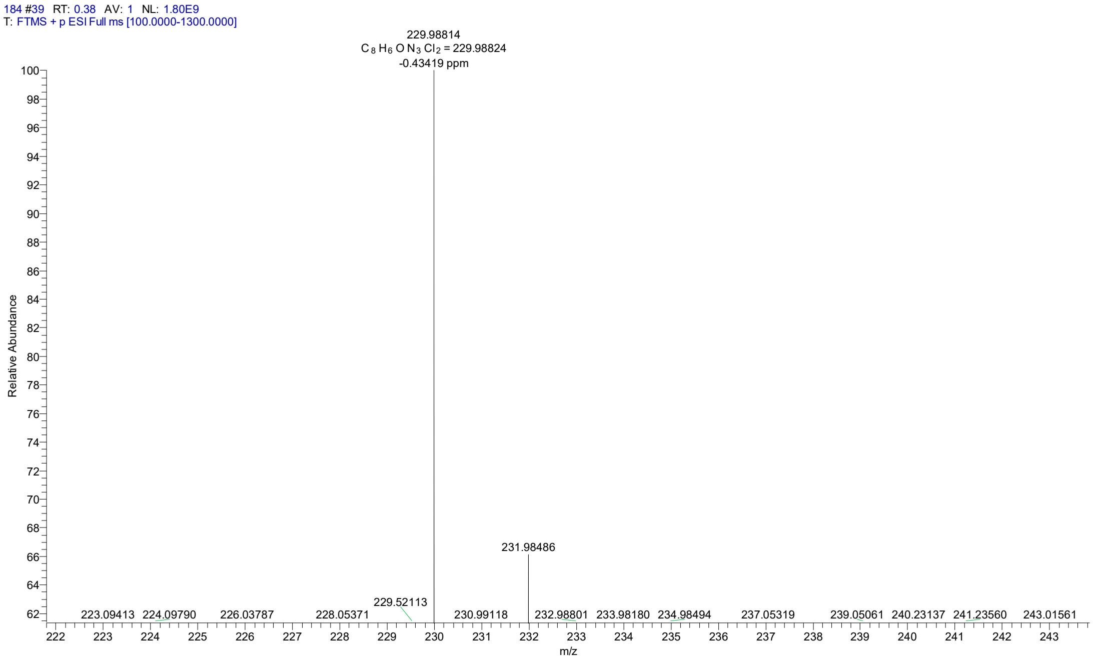

HRMS of compound **A6**

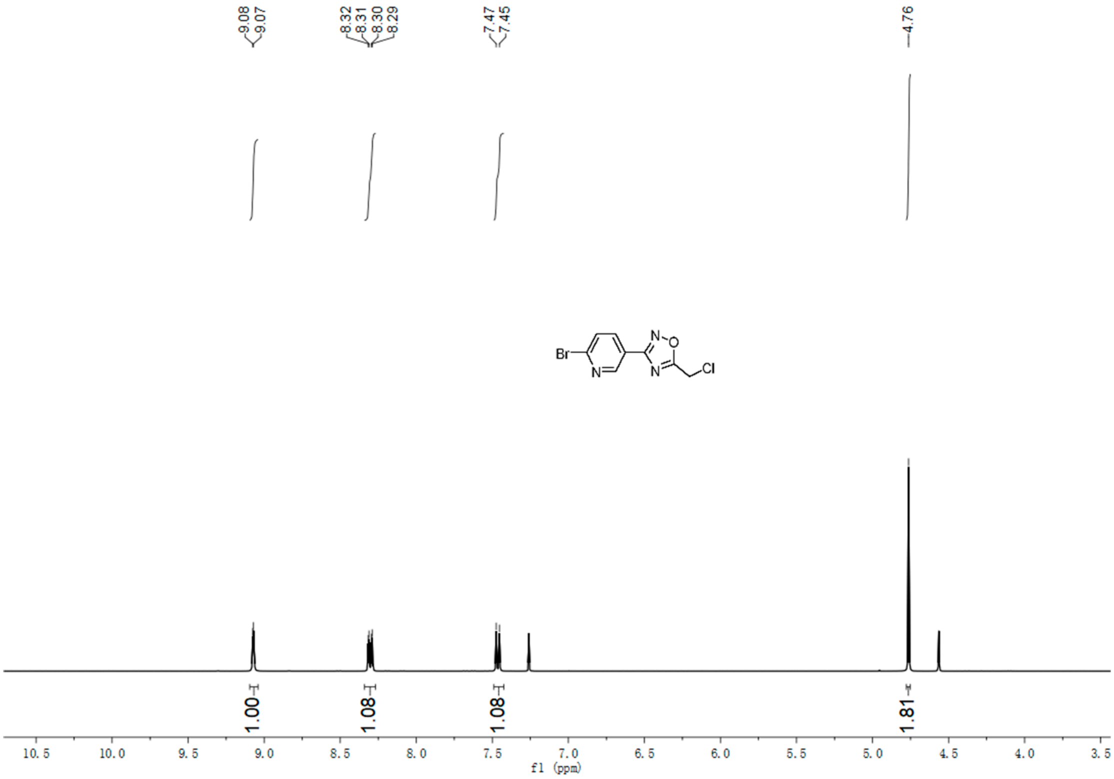

^{1}H NMR of compound **A7**

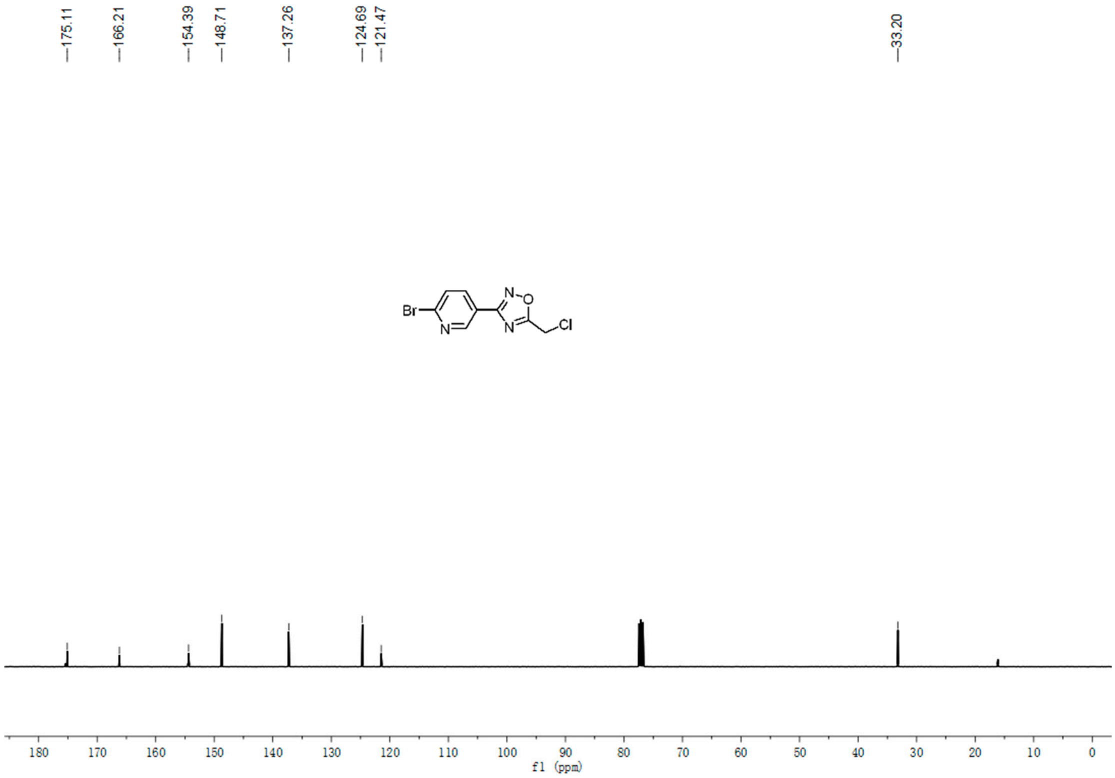

^{13}C NMR of compound **A7**

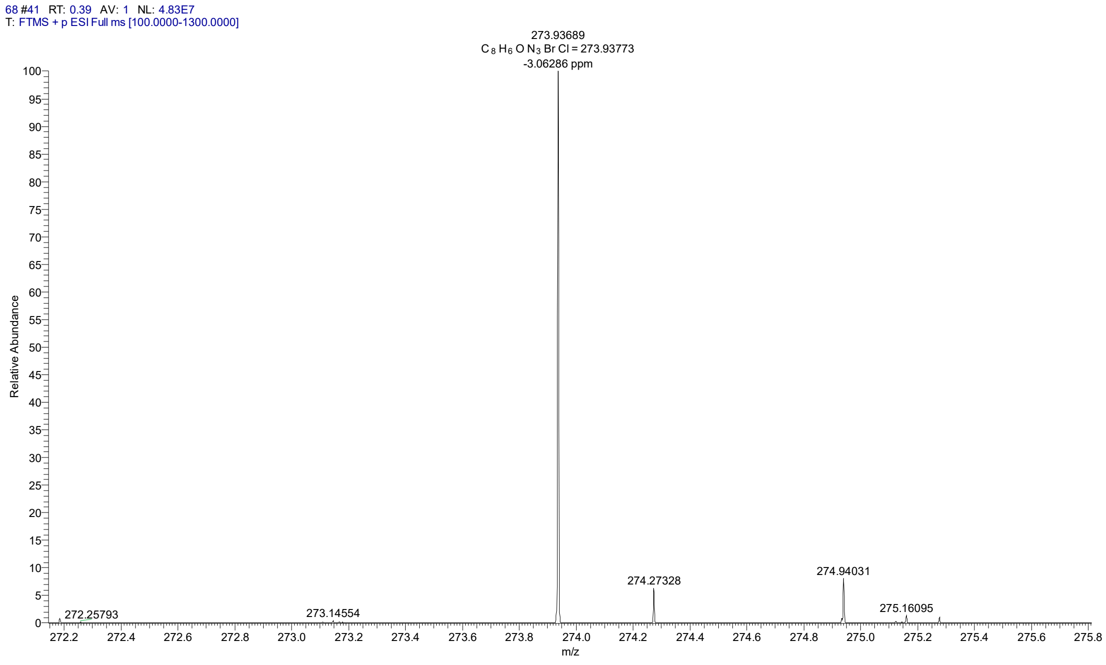

HRMS of compound **A7**

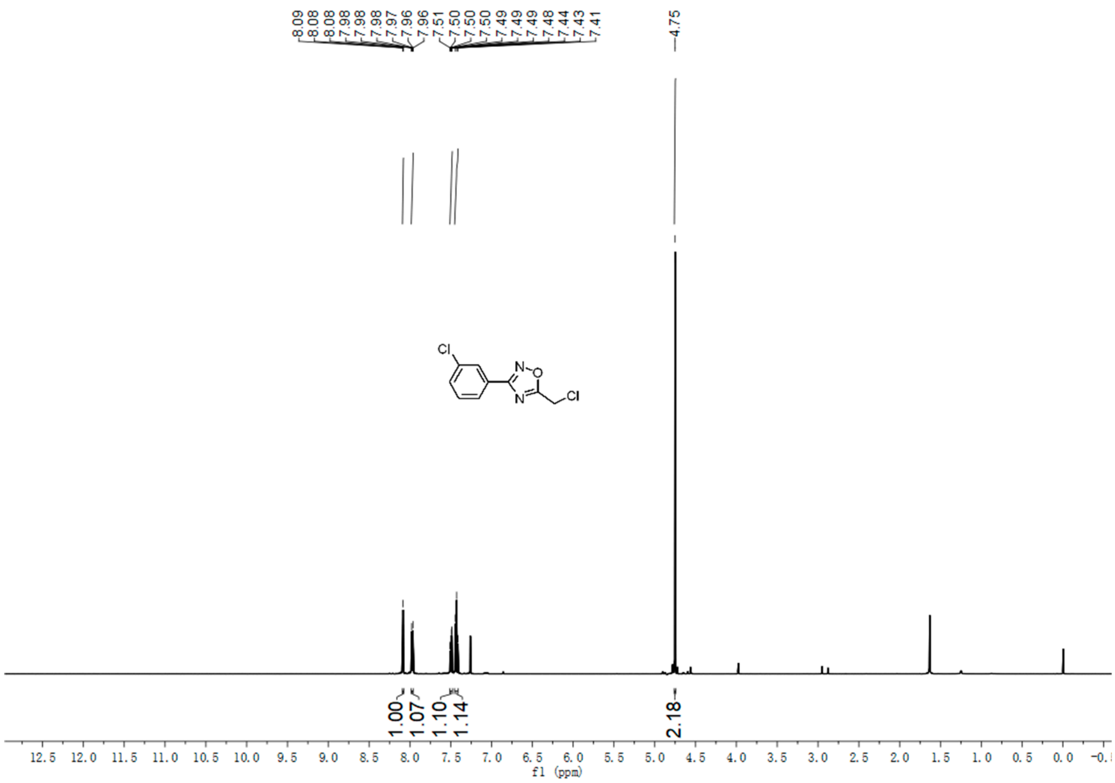

^{1}H NMR of compound **A8**

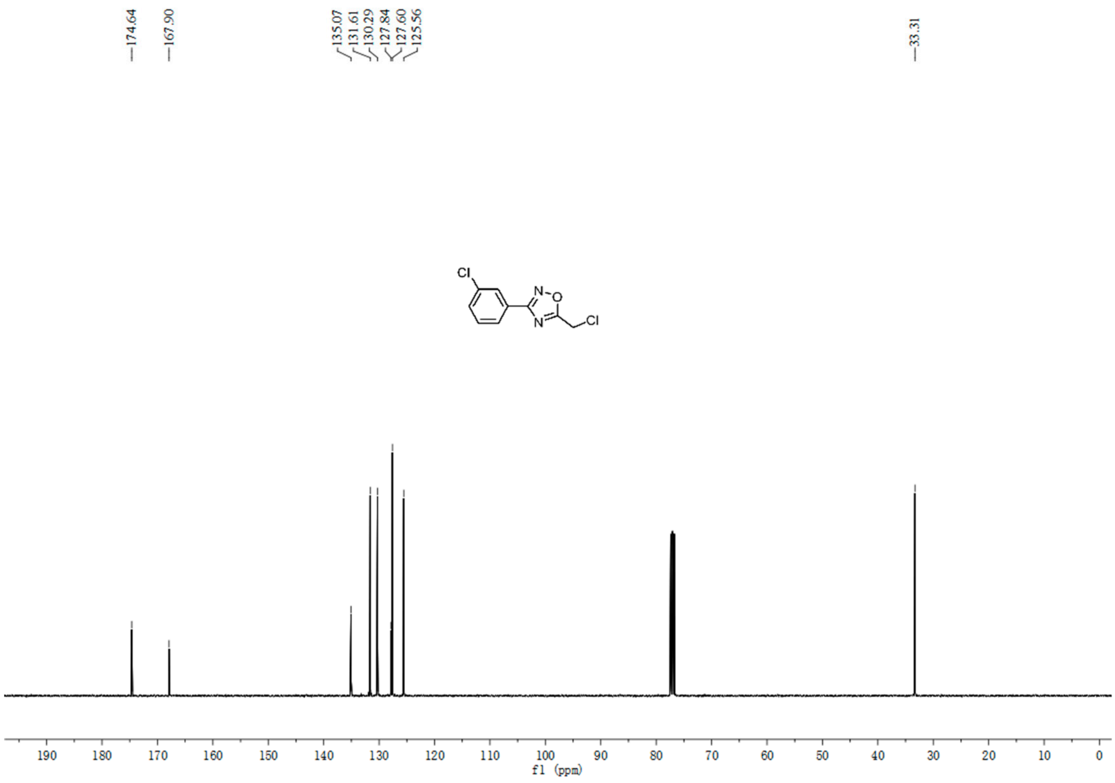

^{13}C NMR of compound **A8**

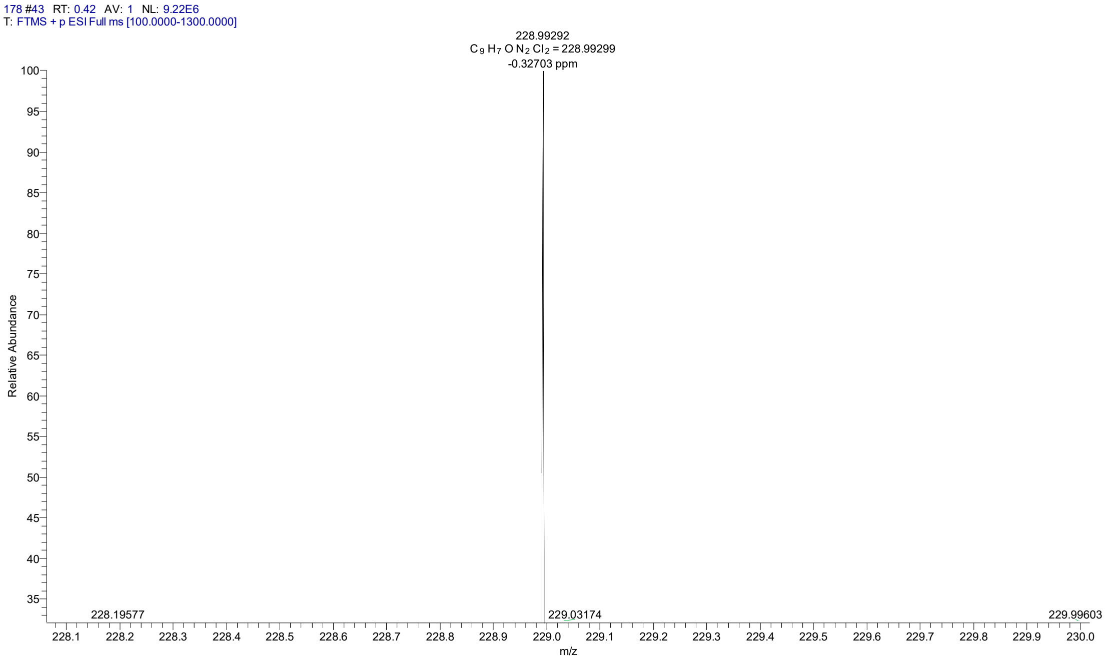

HRMS of compound **A8**

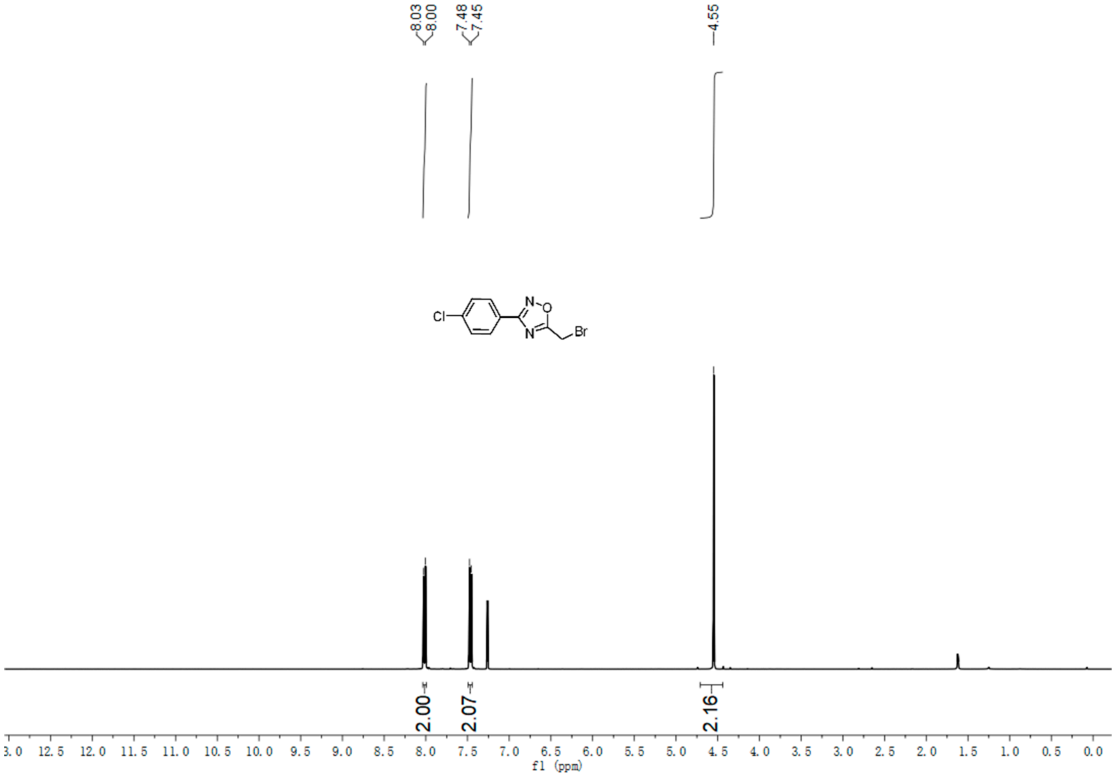

^{1}H NMR of compound **A9**

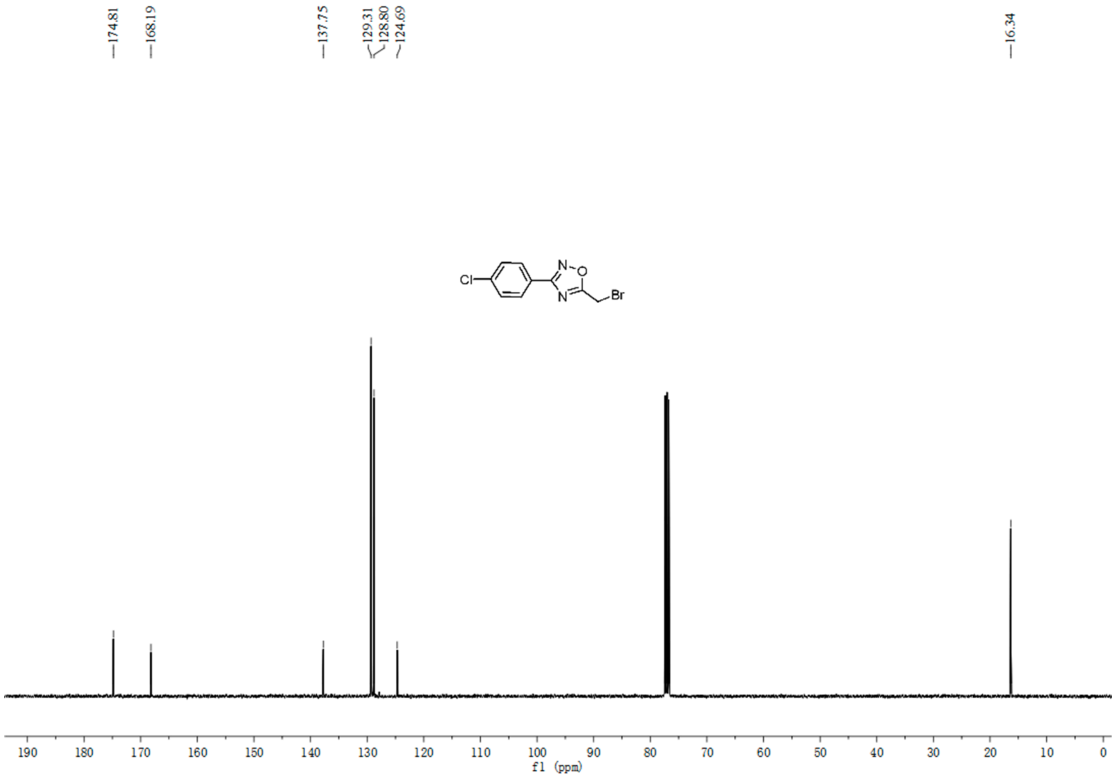

^{13}C NMR of compound **A9**

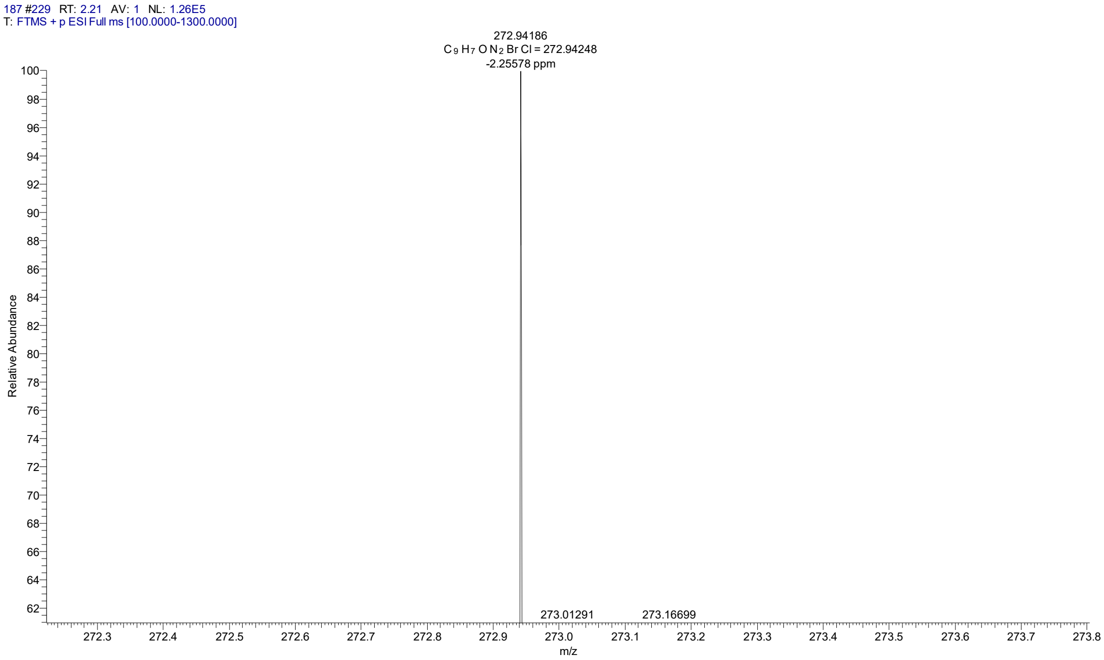

HRMS of compound **A9**

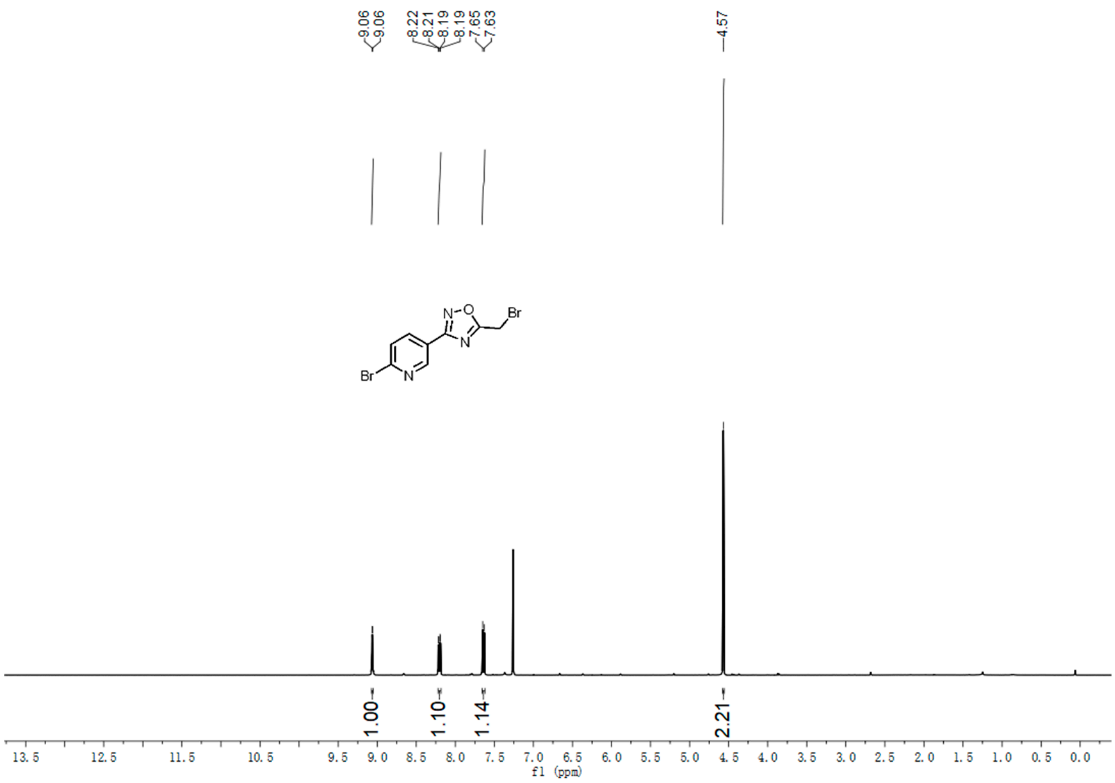

^{1}H NMR of compound **A10**

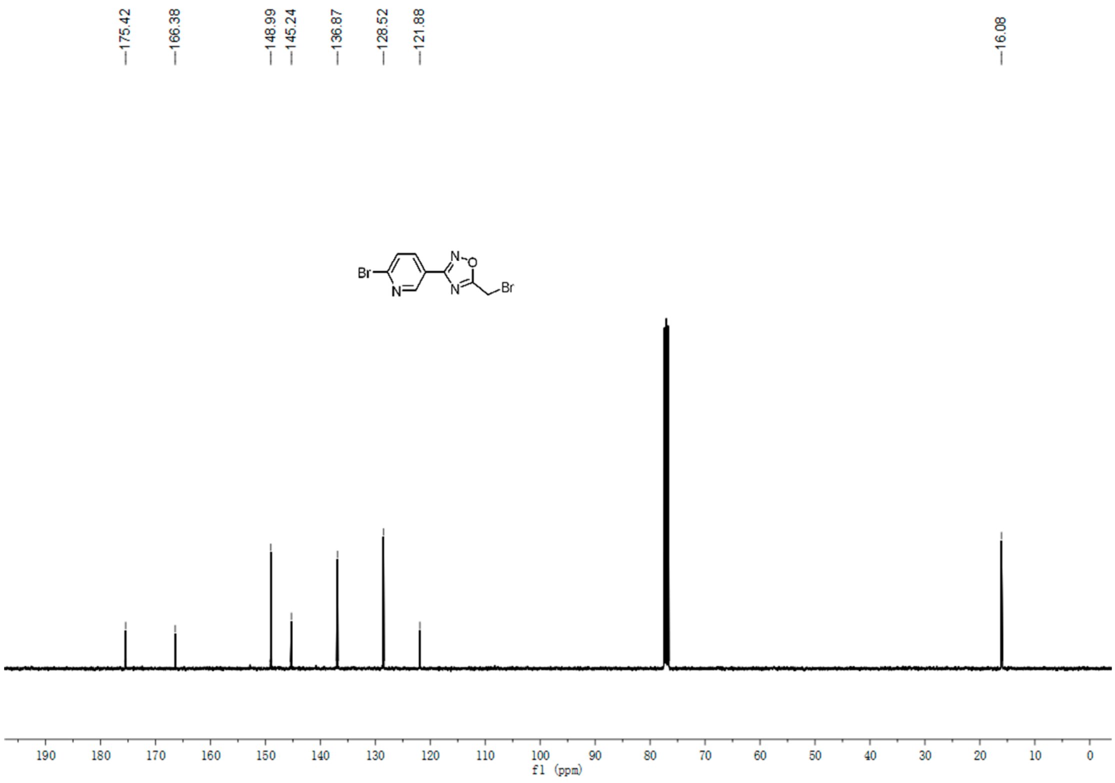

^{13}C NMR of compound **A10**

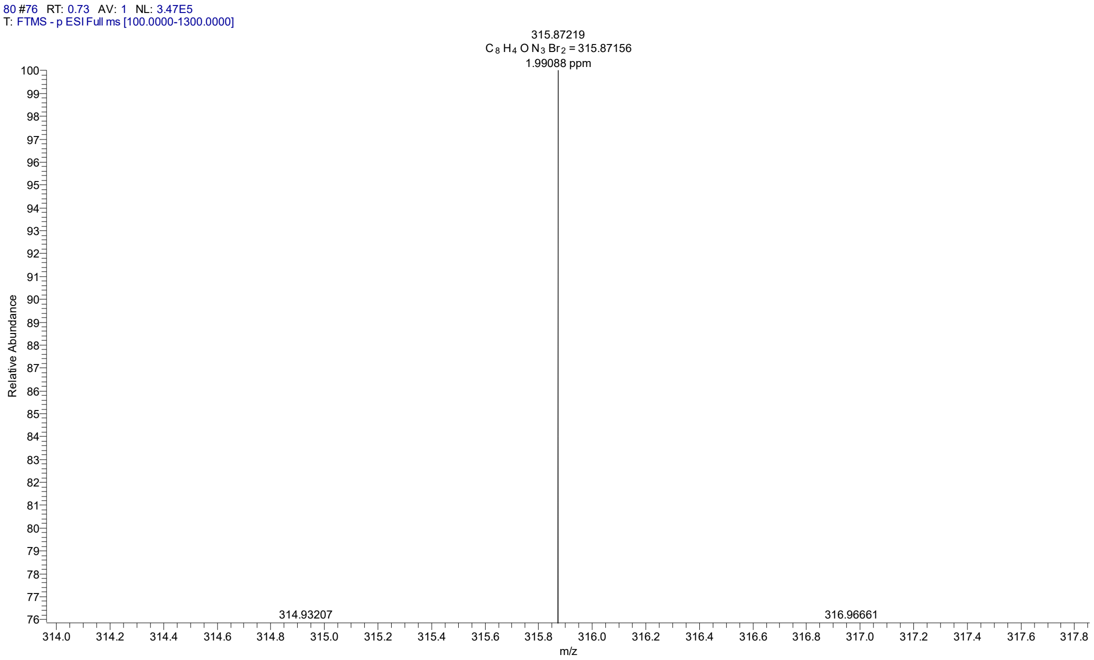

HRMS of compound **A10**

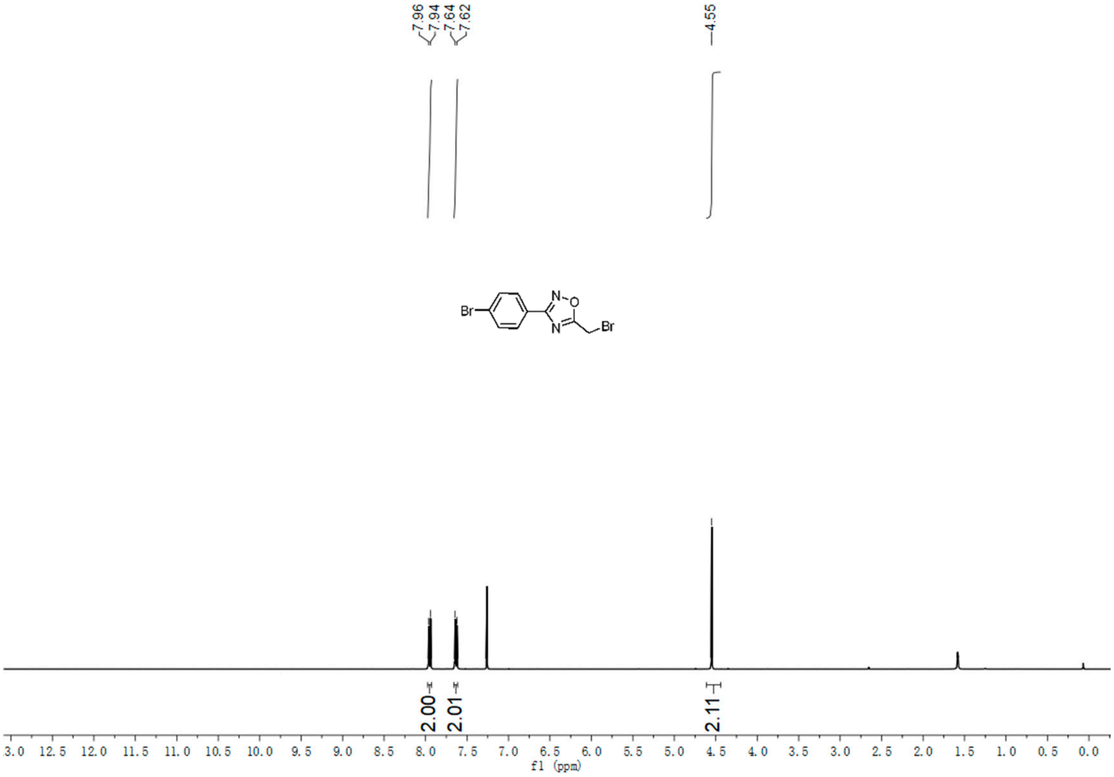

^{1}H NMR of compound **A11**

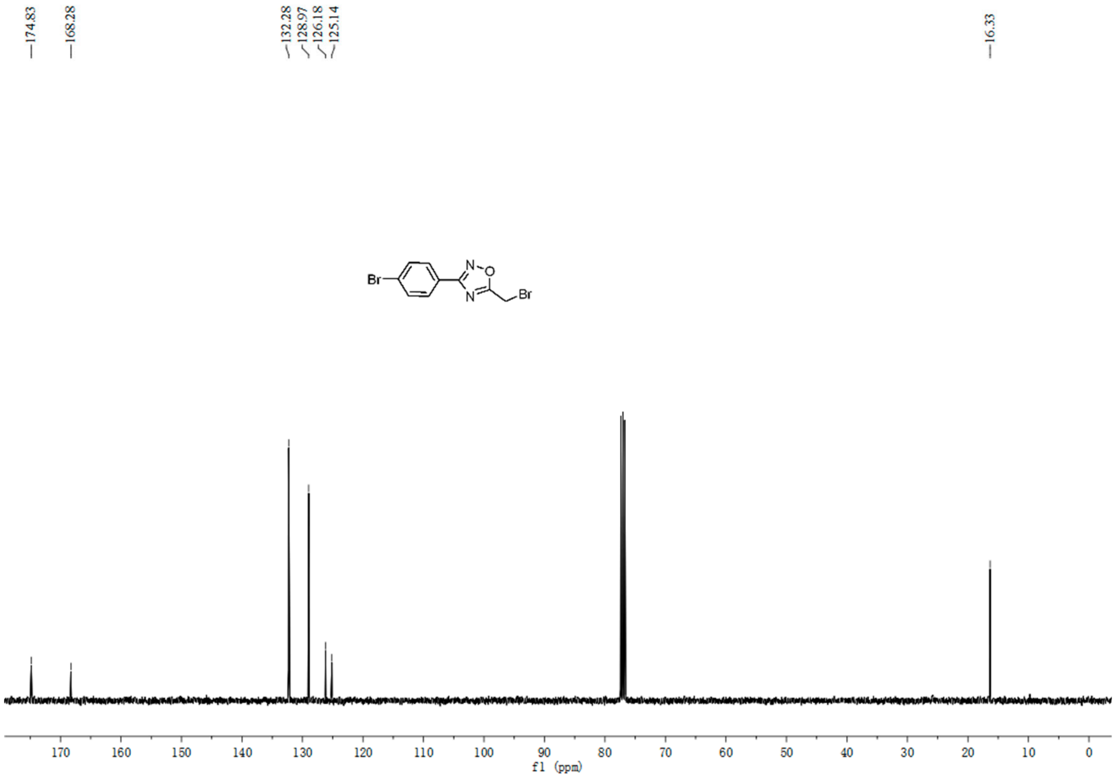

^{13}C NMR of compound **A11**

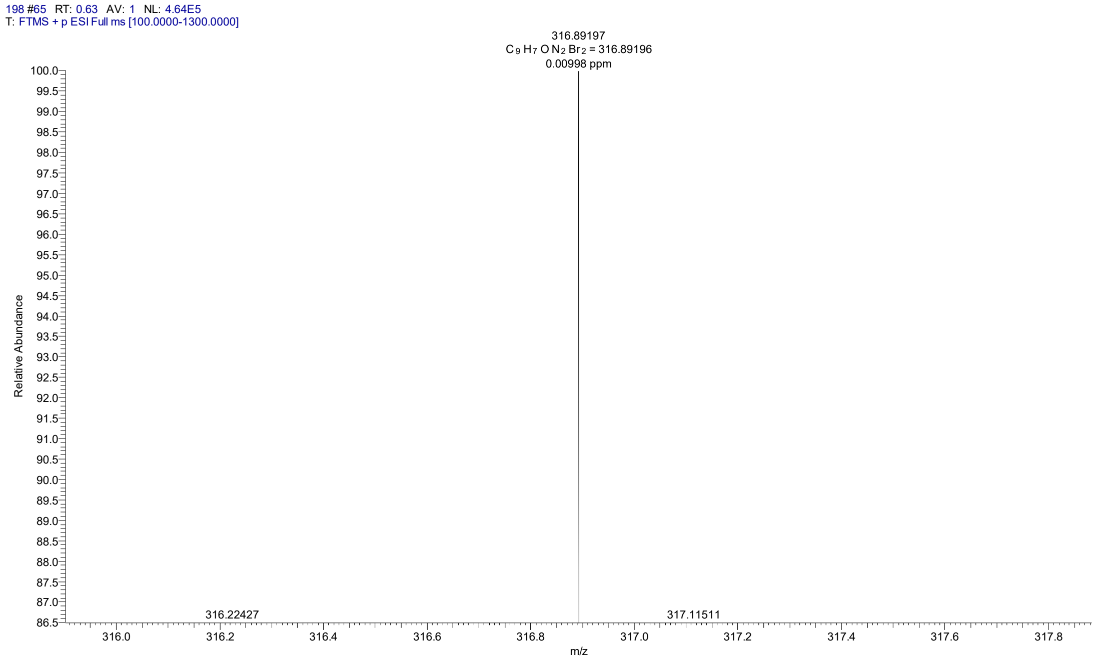

HRMS of compound **A11**

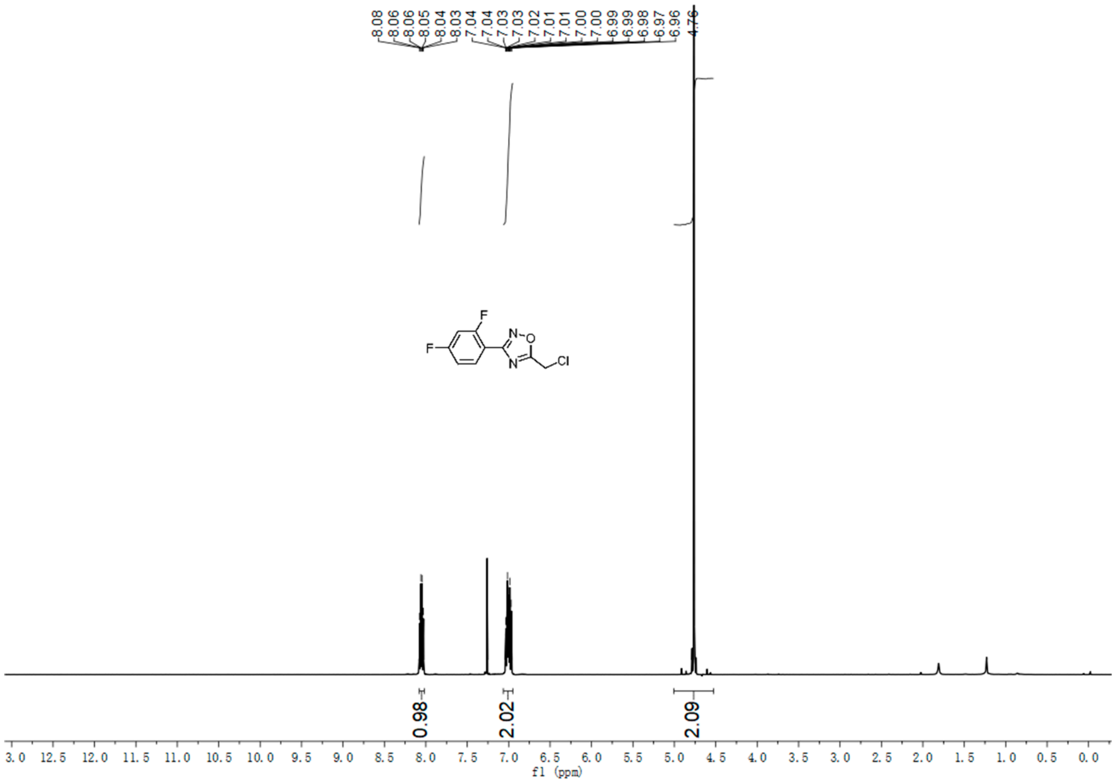

^{1}H NMR of compound **A12**

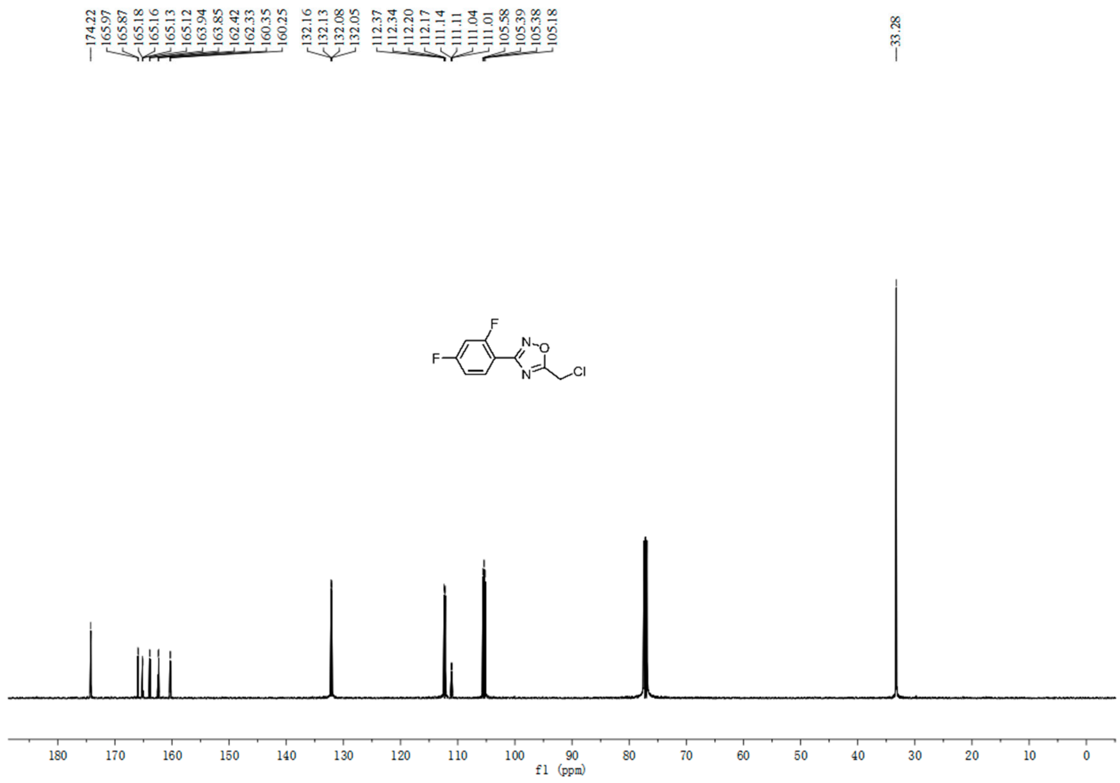

^{13}C NMR of compound **A12**

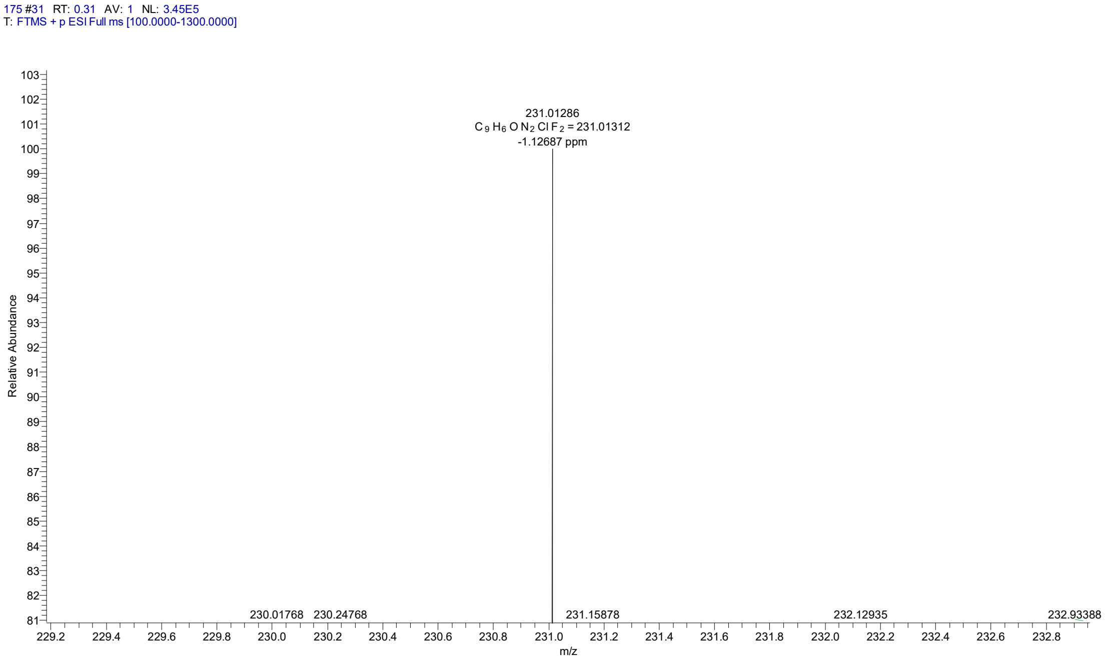

HRMS of compound **A12**

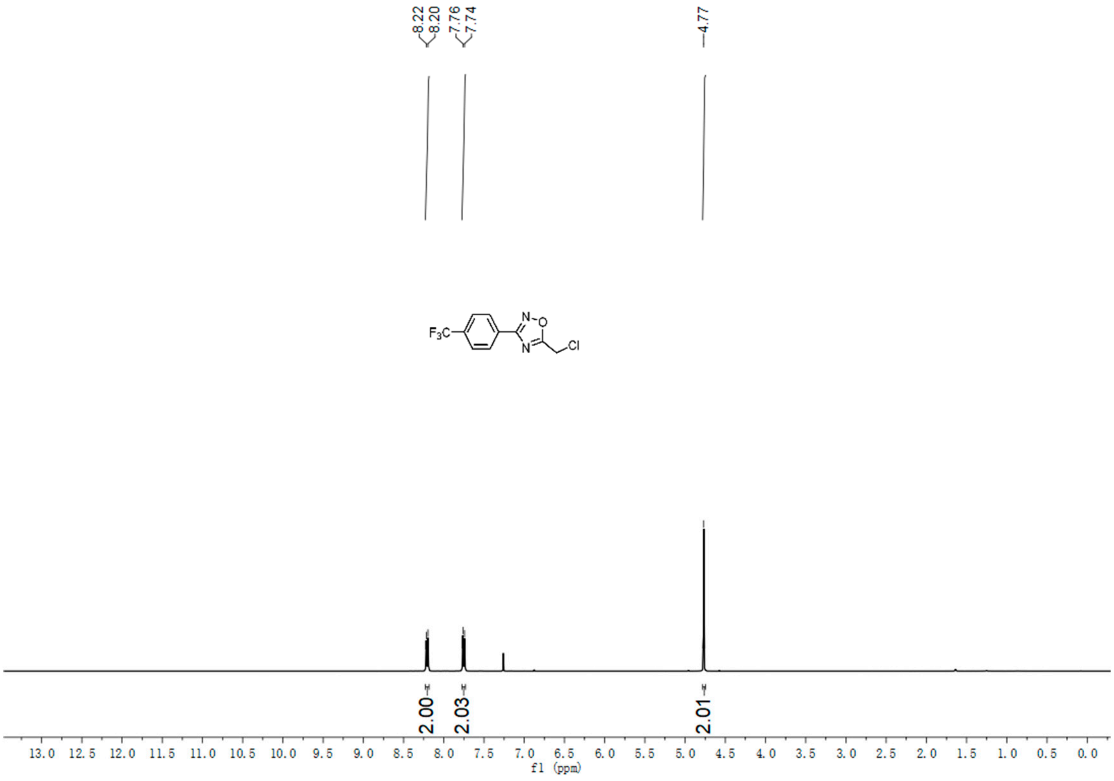

^{1}H NMR of compound **A13**

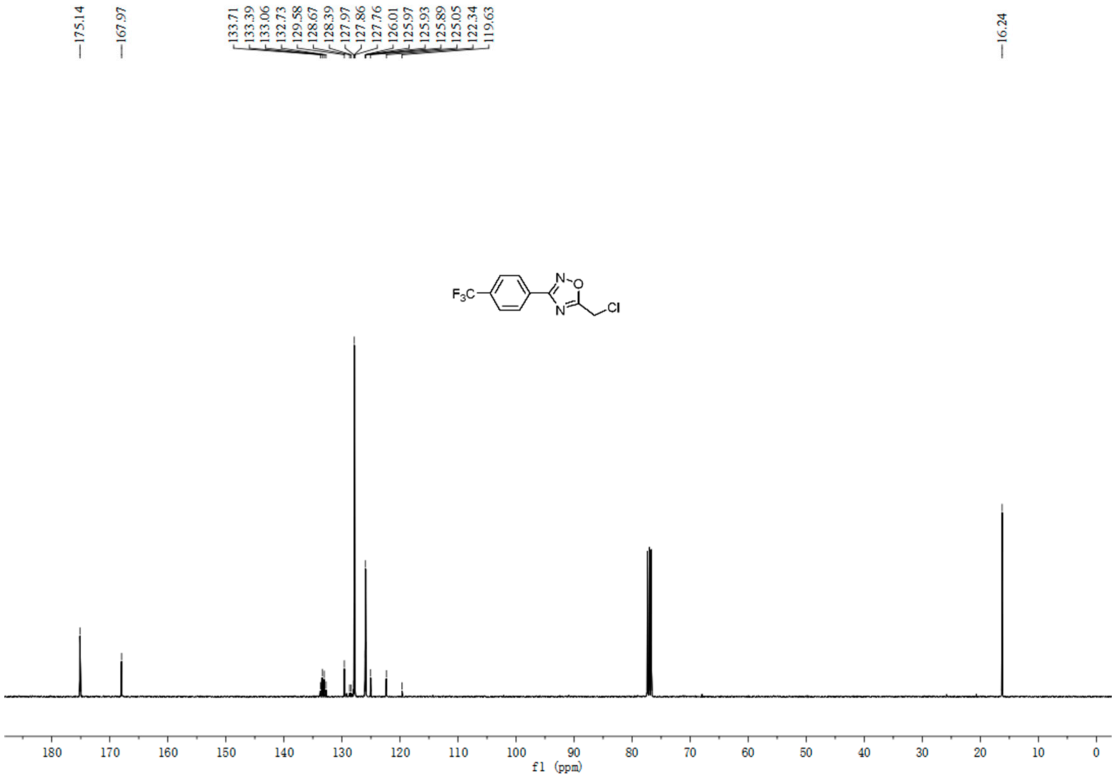

^{13}C NMR of compound **A13**

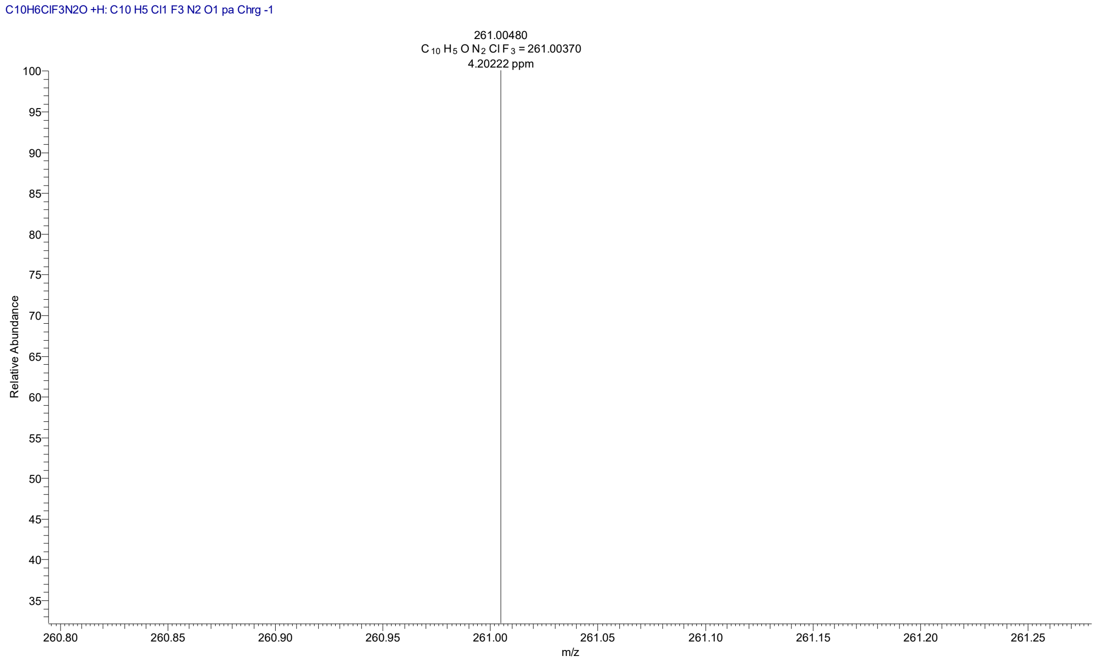

HRMS of compound **A13**

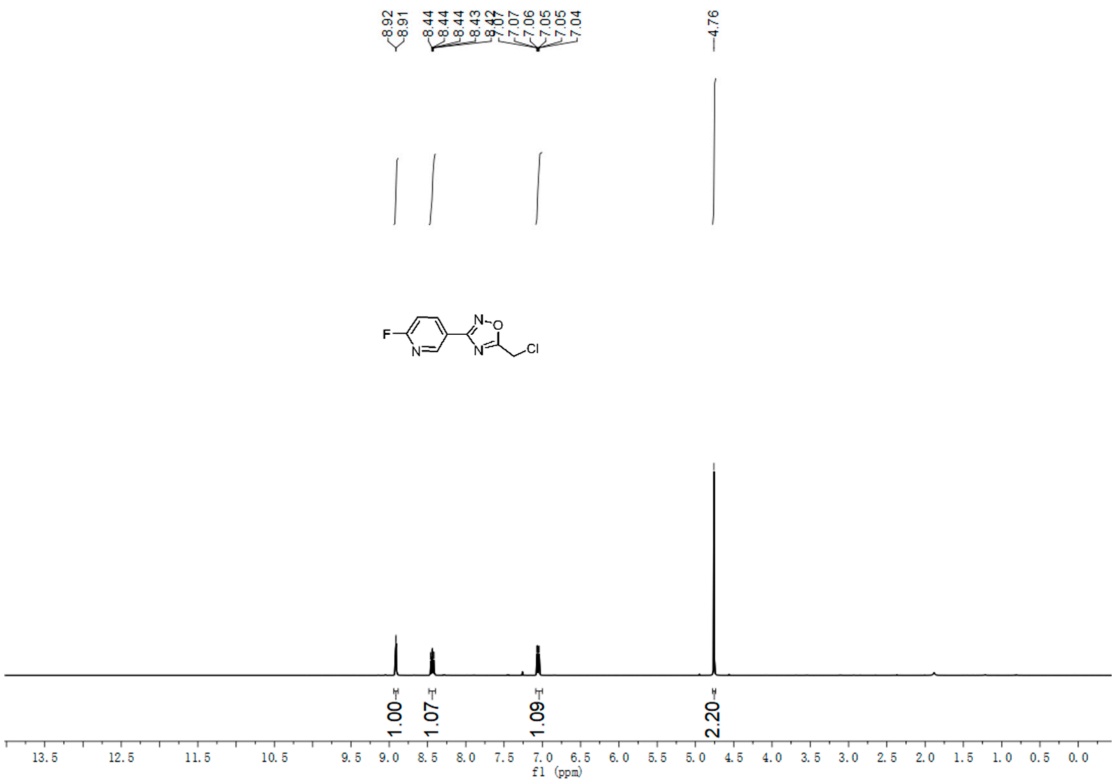

^{1}H NMR of compound **A14**

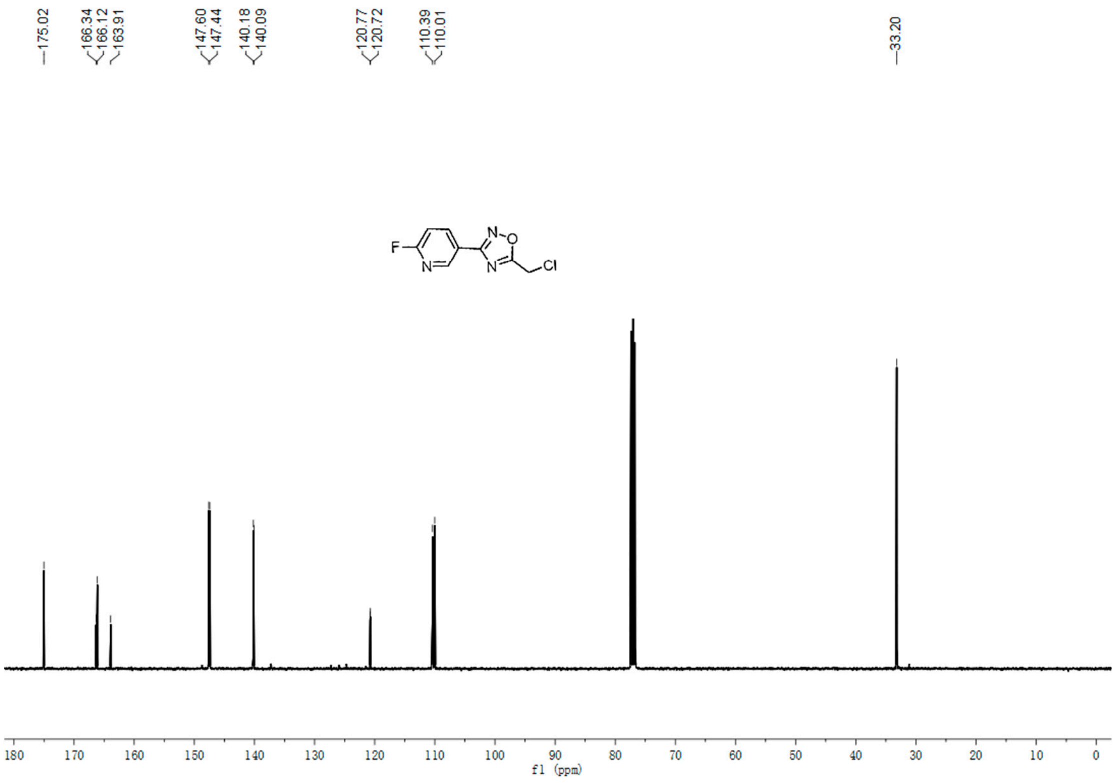

^{13}C NMR of compound **A14**

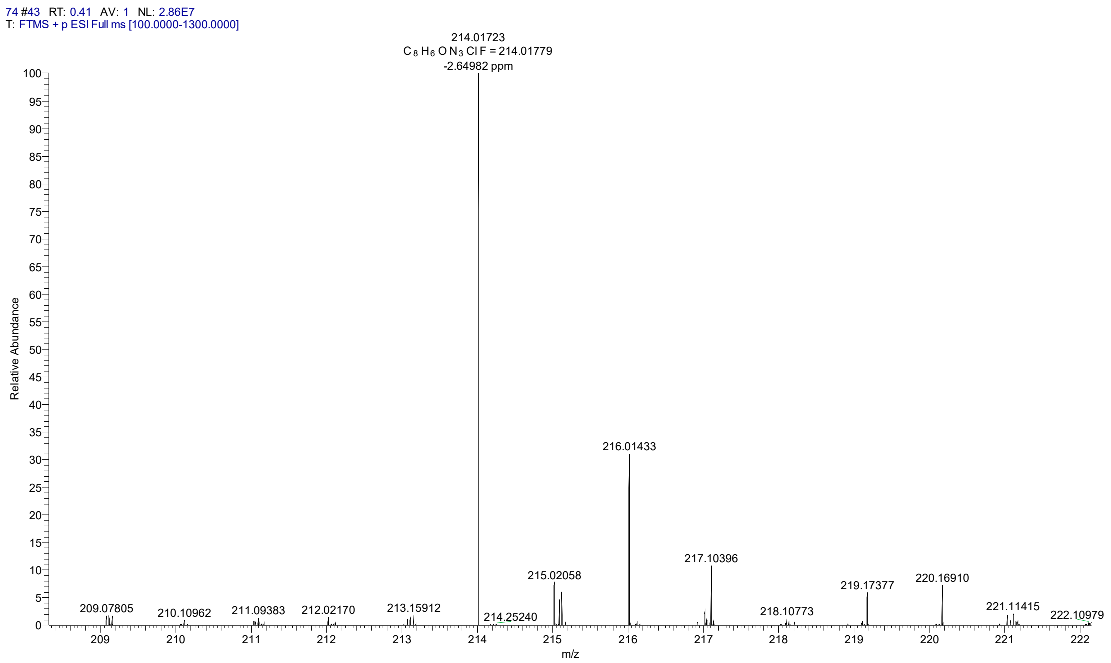

HRMS of compound **A14**

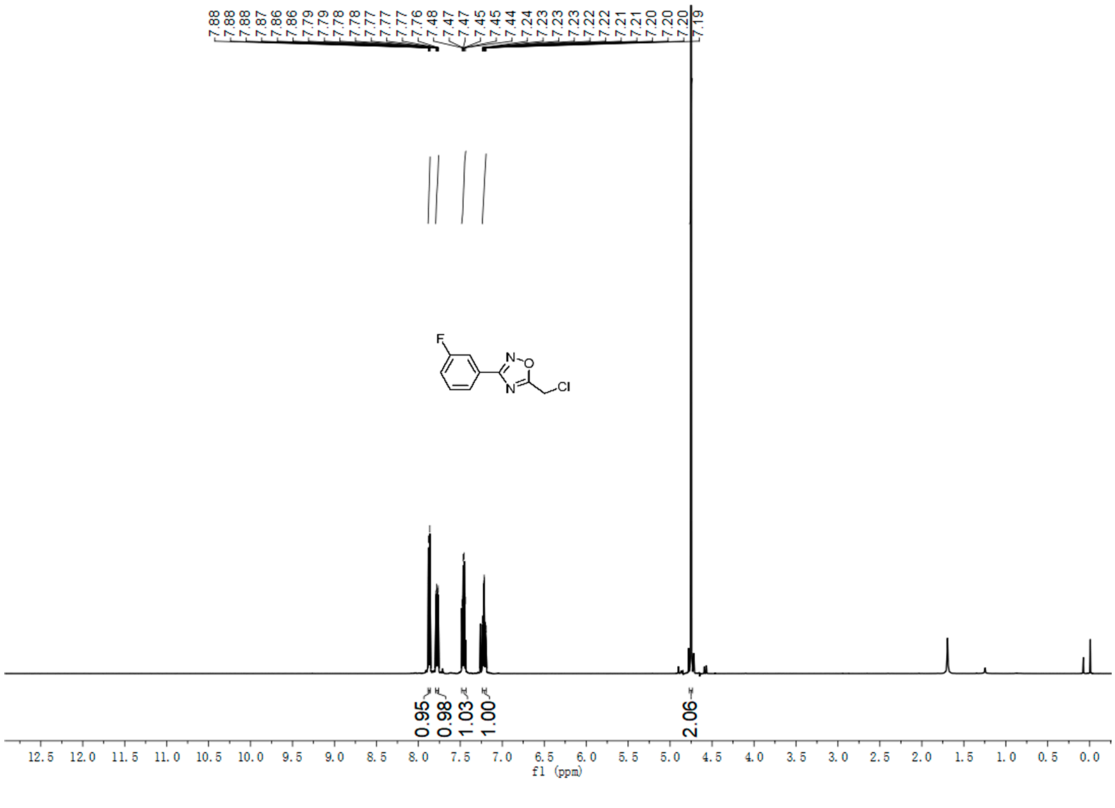

^{1}H NMR of compound **A15**

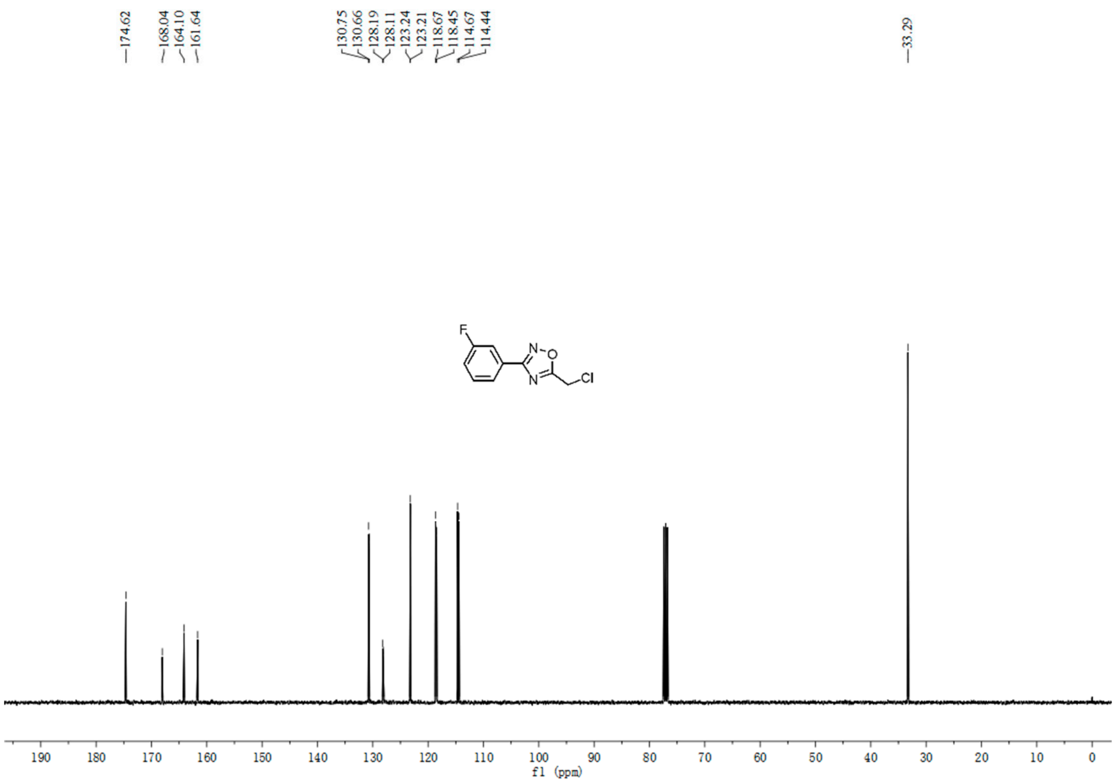

^{13}C NMR of compound **A15**

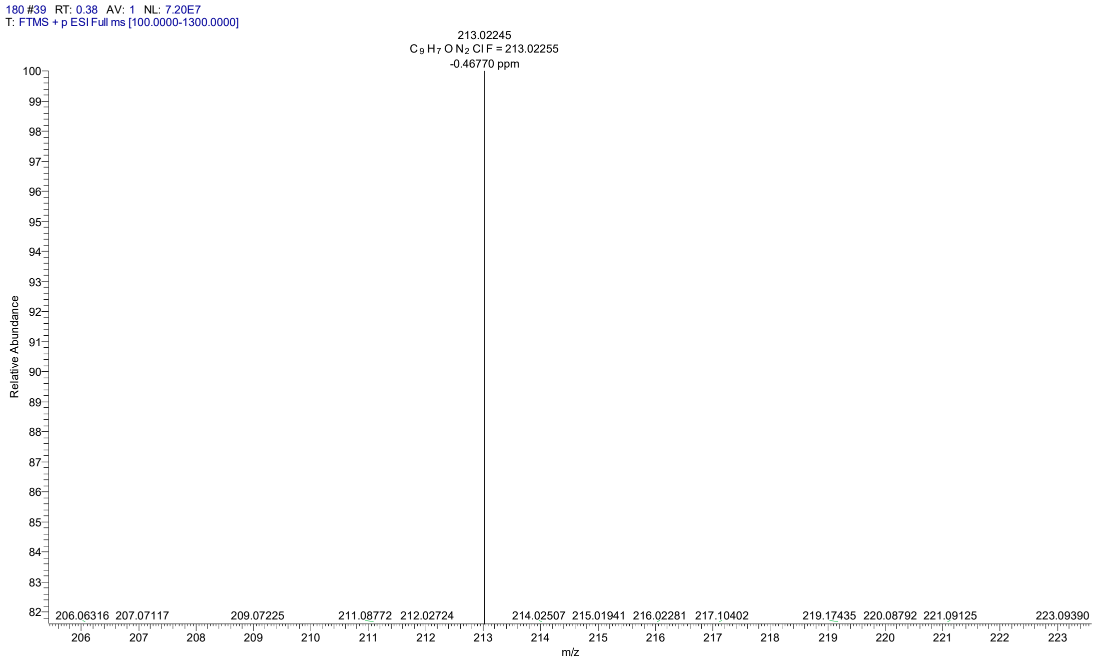

HRMS of compound **A15**

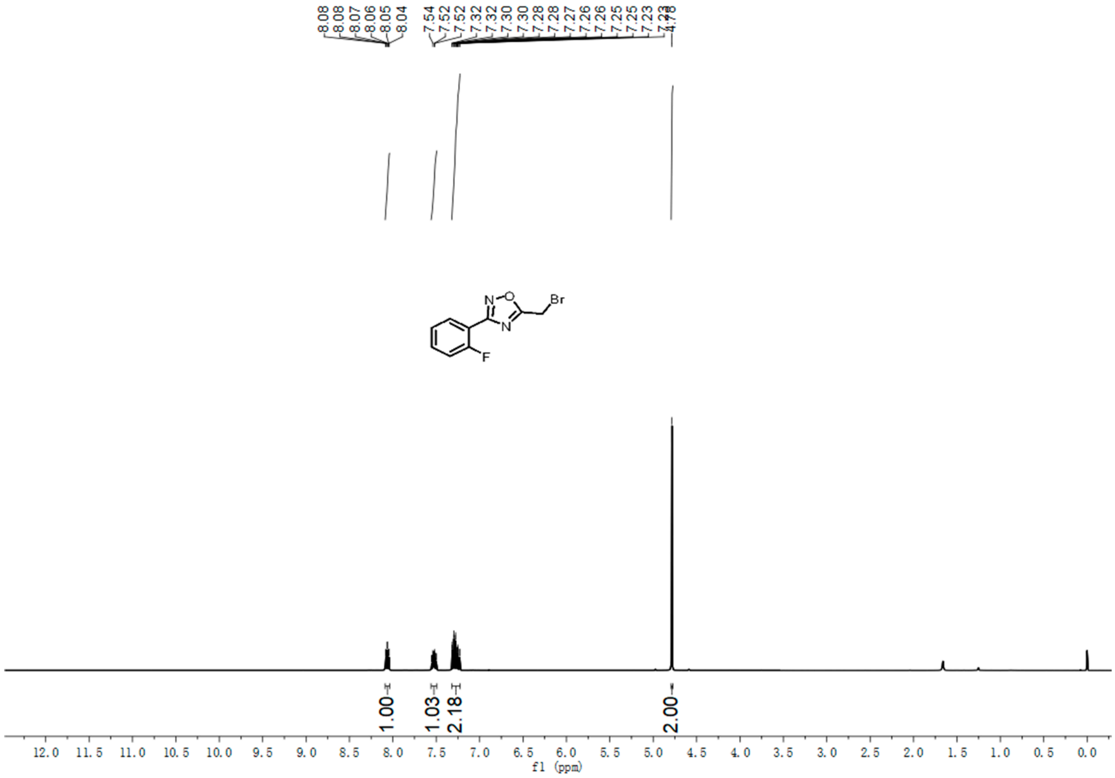

^{1}H NMR of compound **A16**

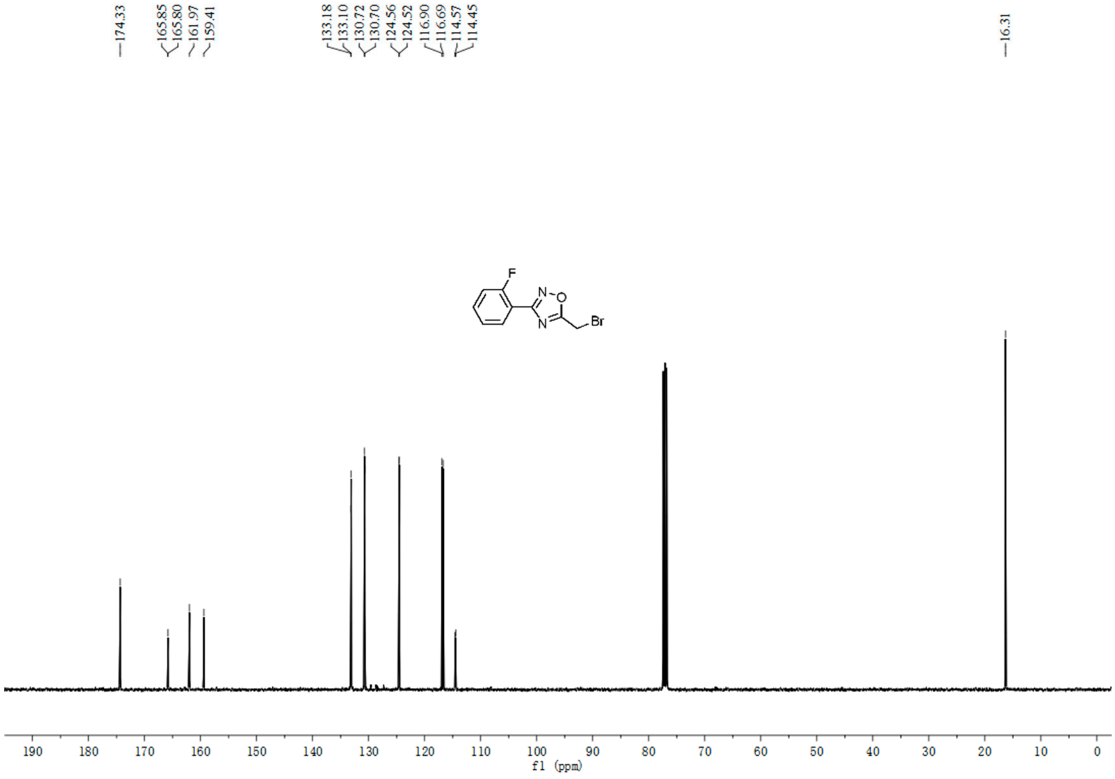

^{13}C NMR of compound **A16**

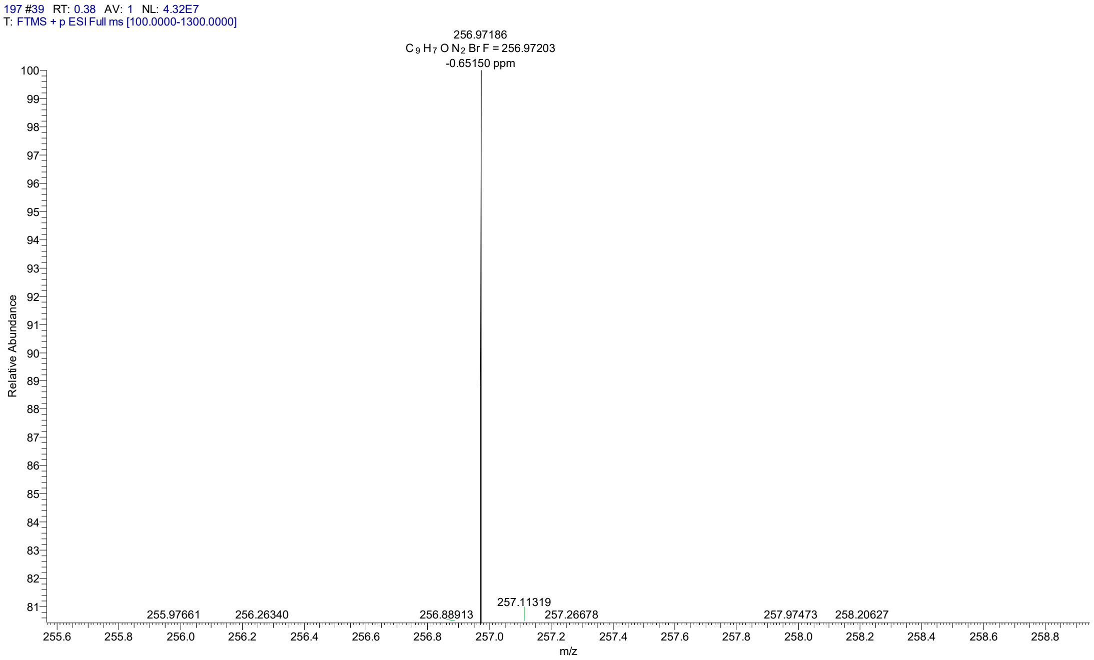

HRMS of compound **A16**

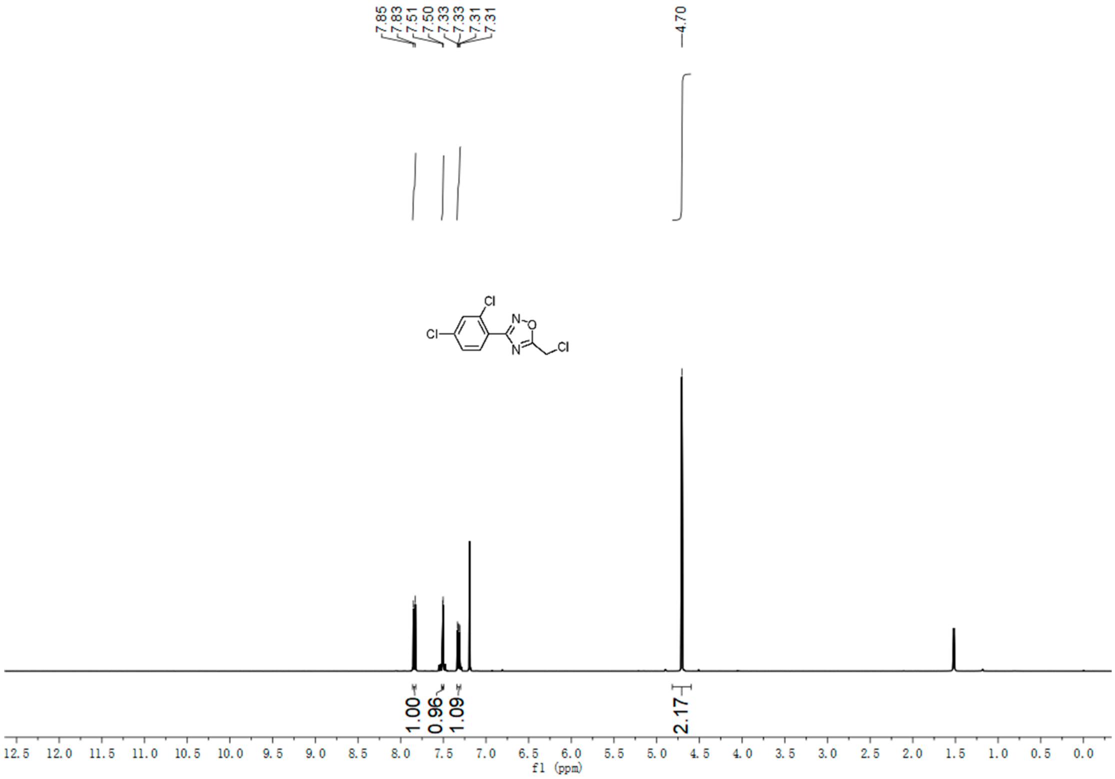

^{1}H NMR of compound **A17**

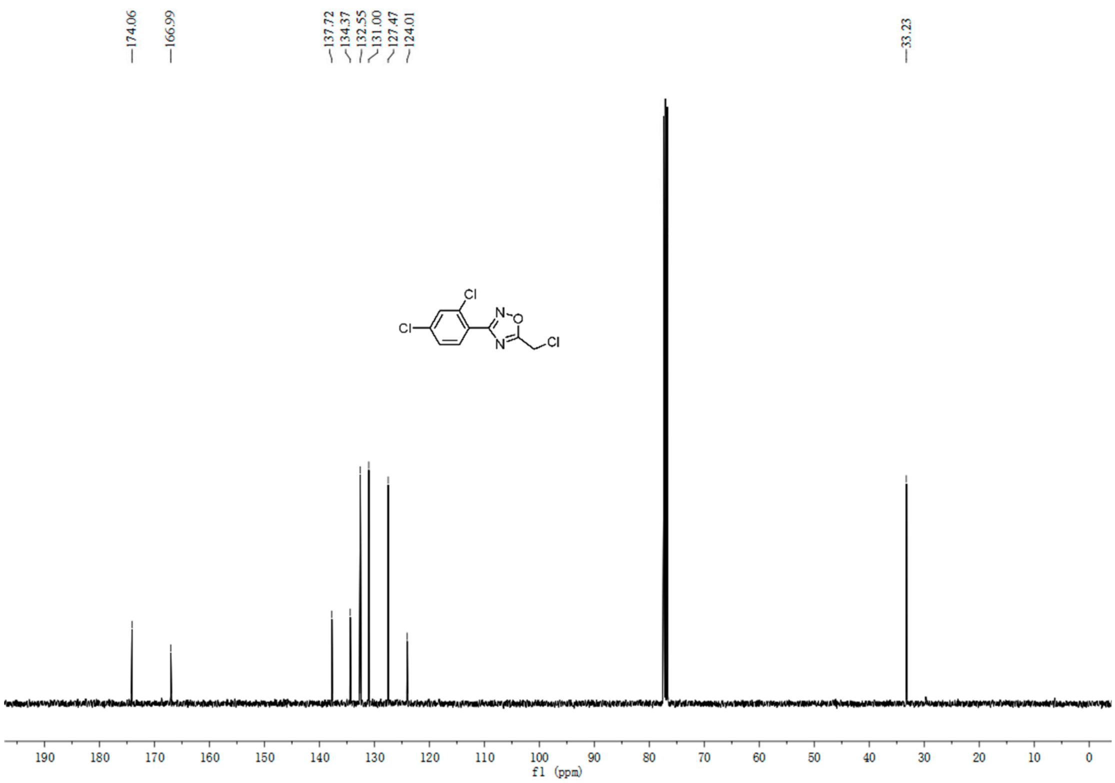

^{13}C NMR of compound **A17**

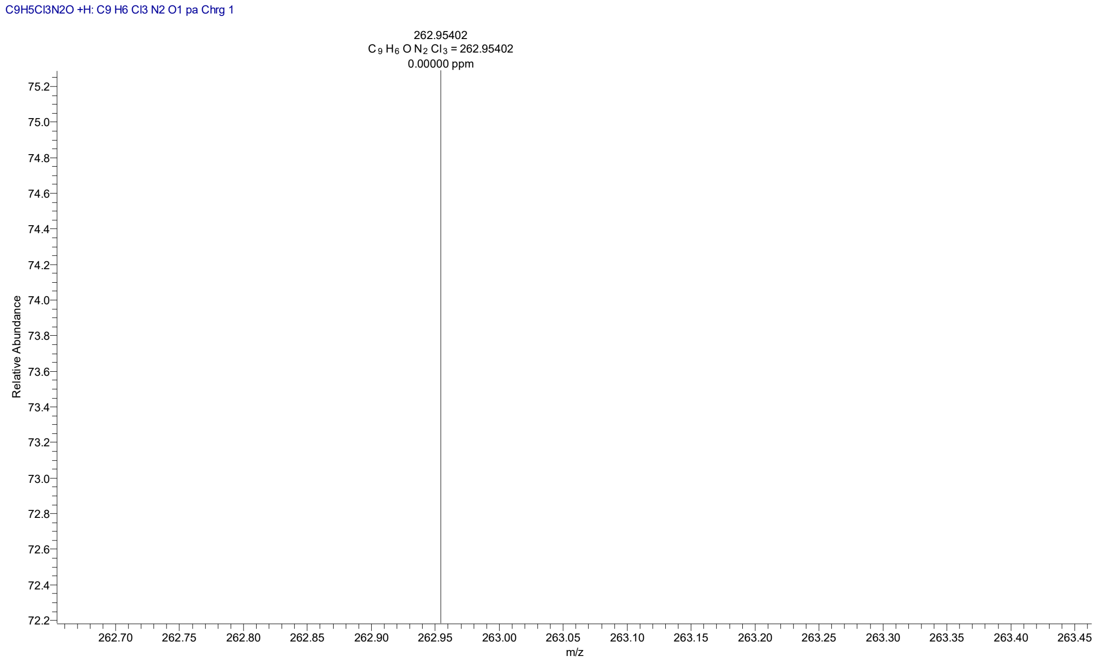

HRMS of compound **A17**

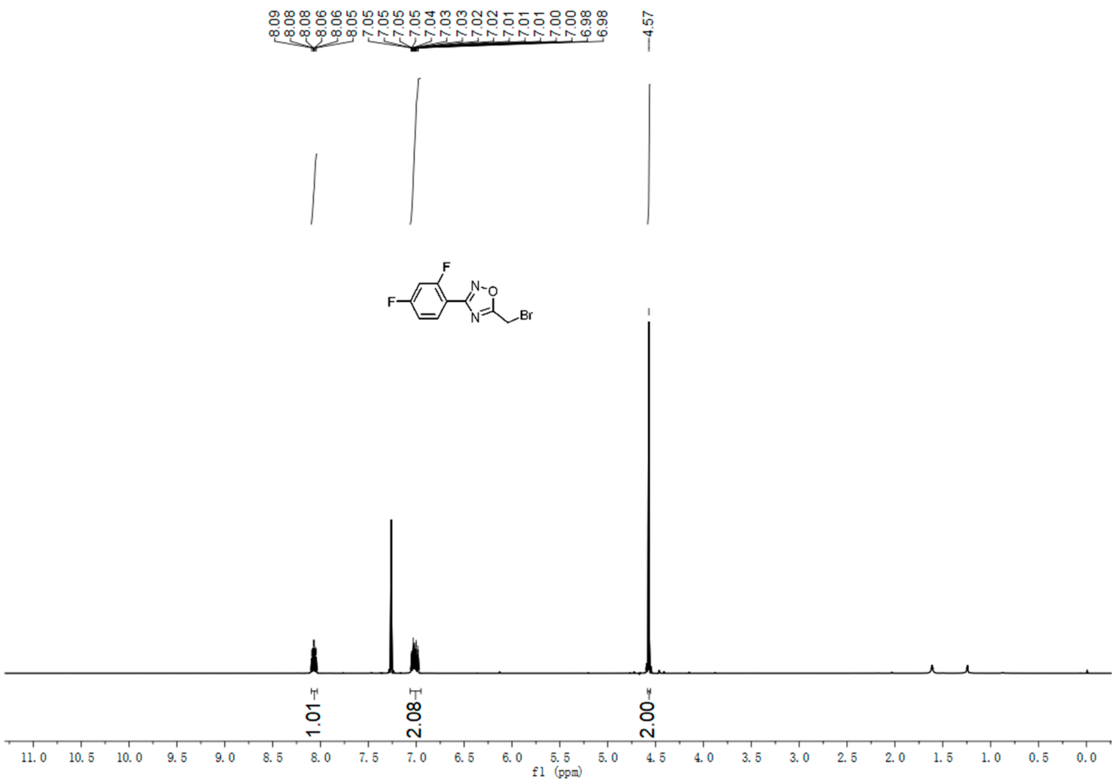

^{1}H NMR of compound **A18**

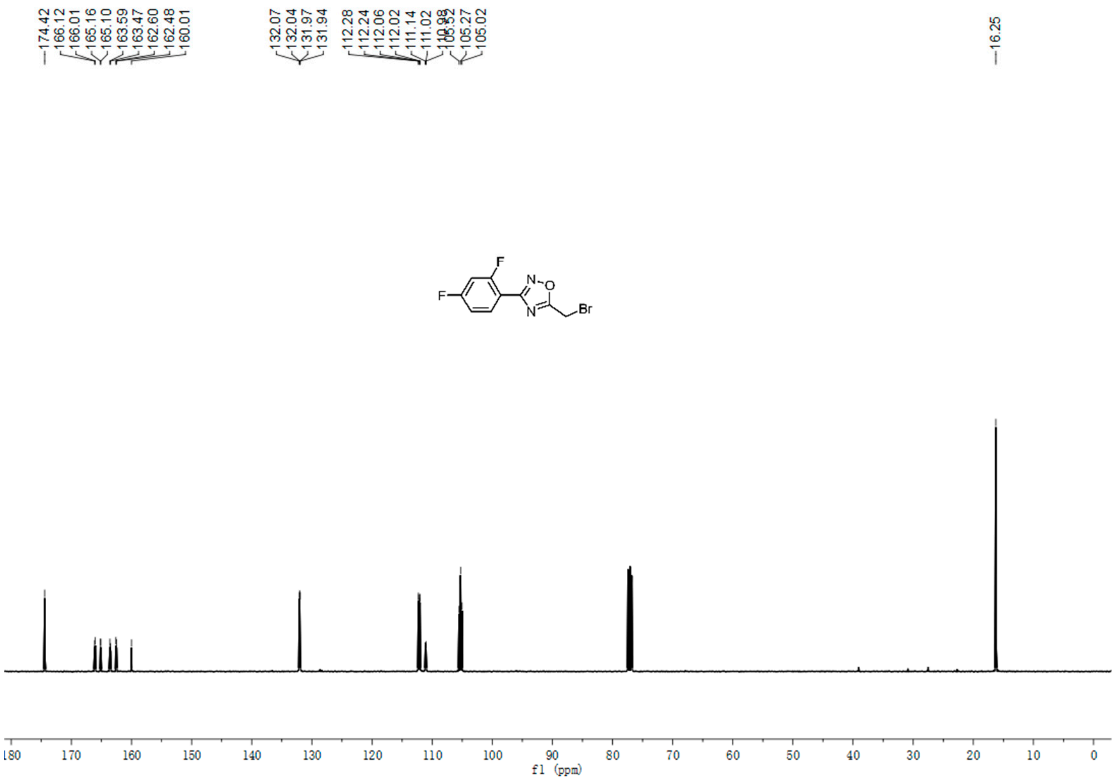

^{13}C NMR of compound **A18**

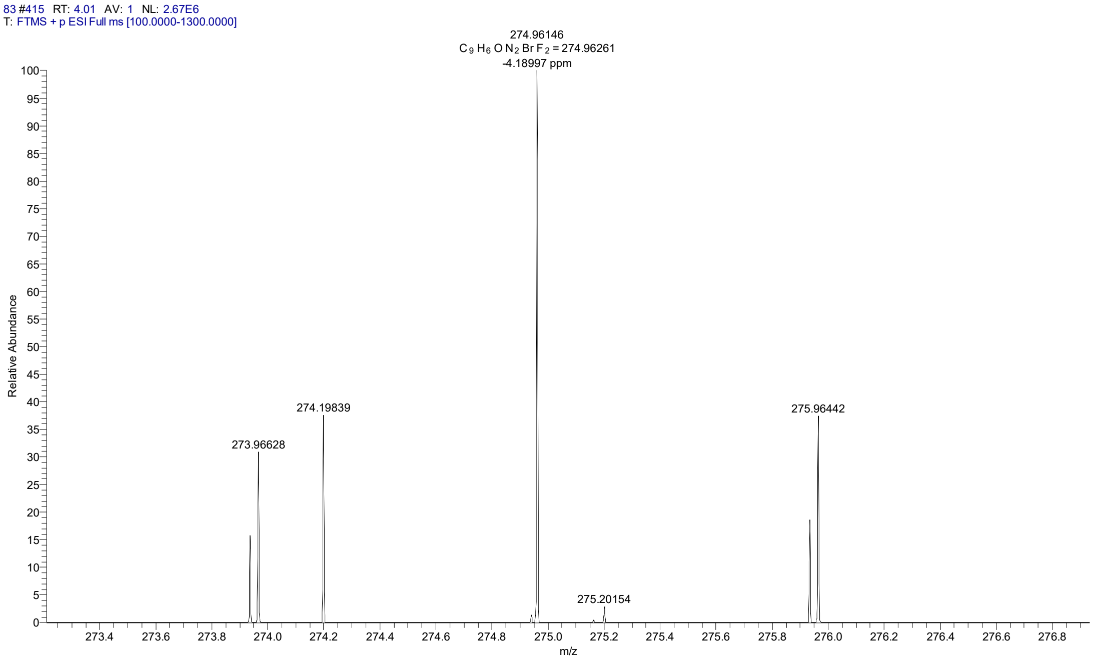

HRMS of compound **A18**

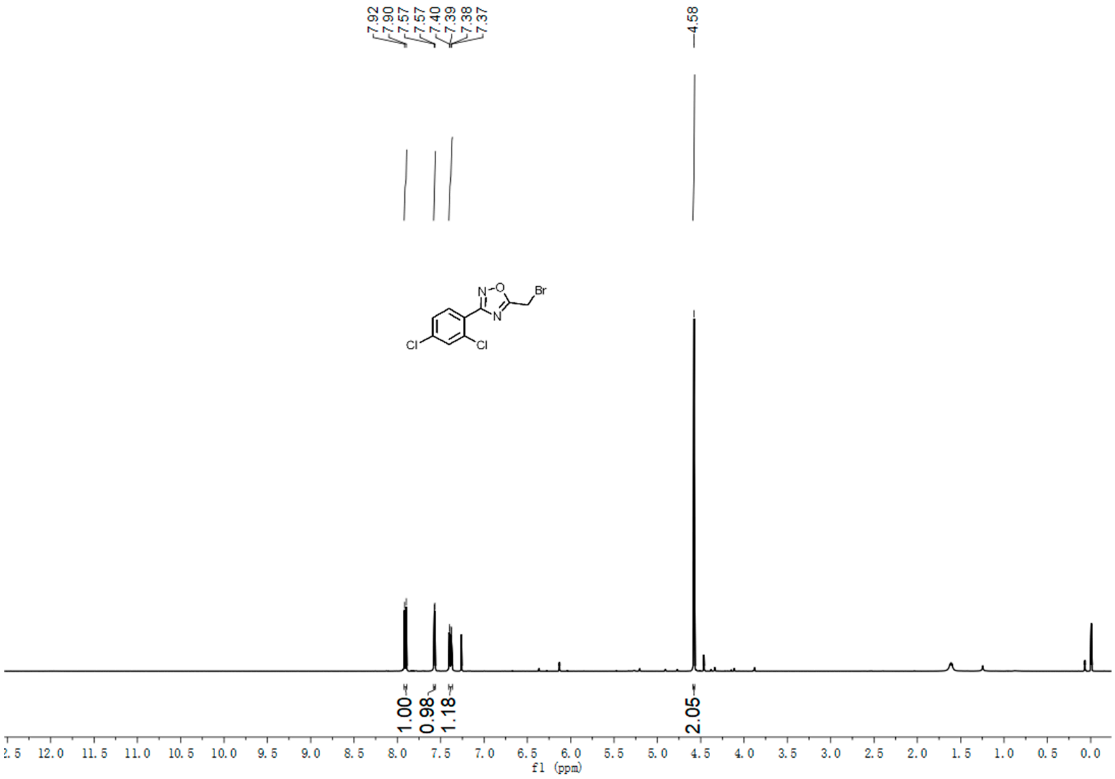

^{1}H NMR of compound **A19**

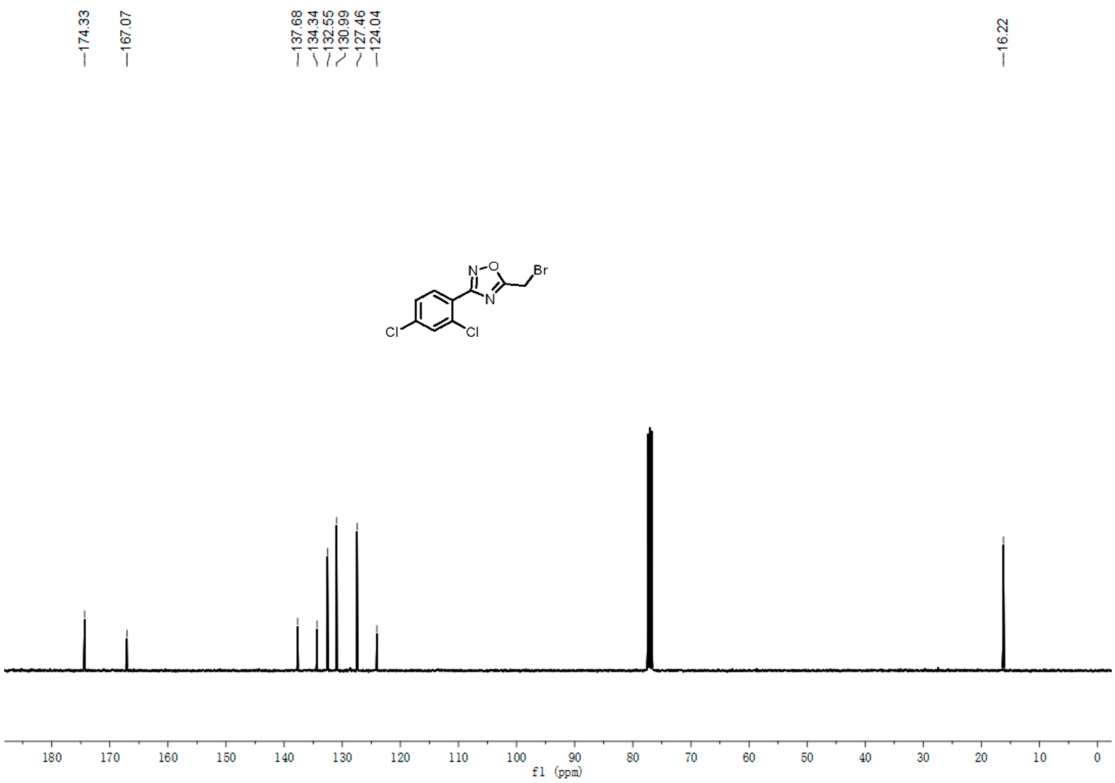

^{13}C NMR of compound **A19**

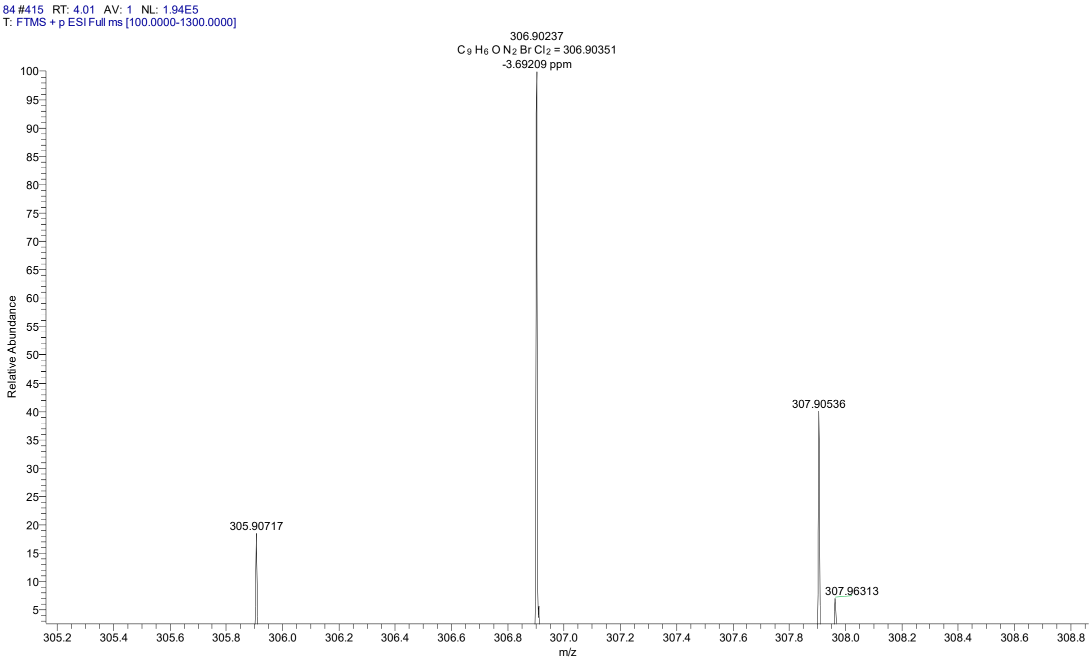

HRMS of compound **A19**

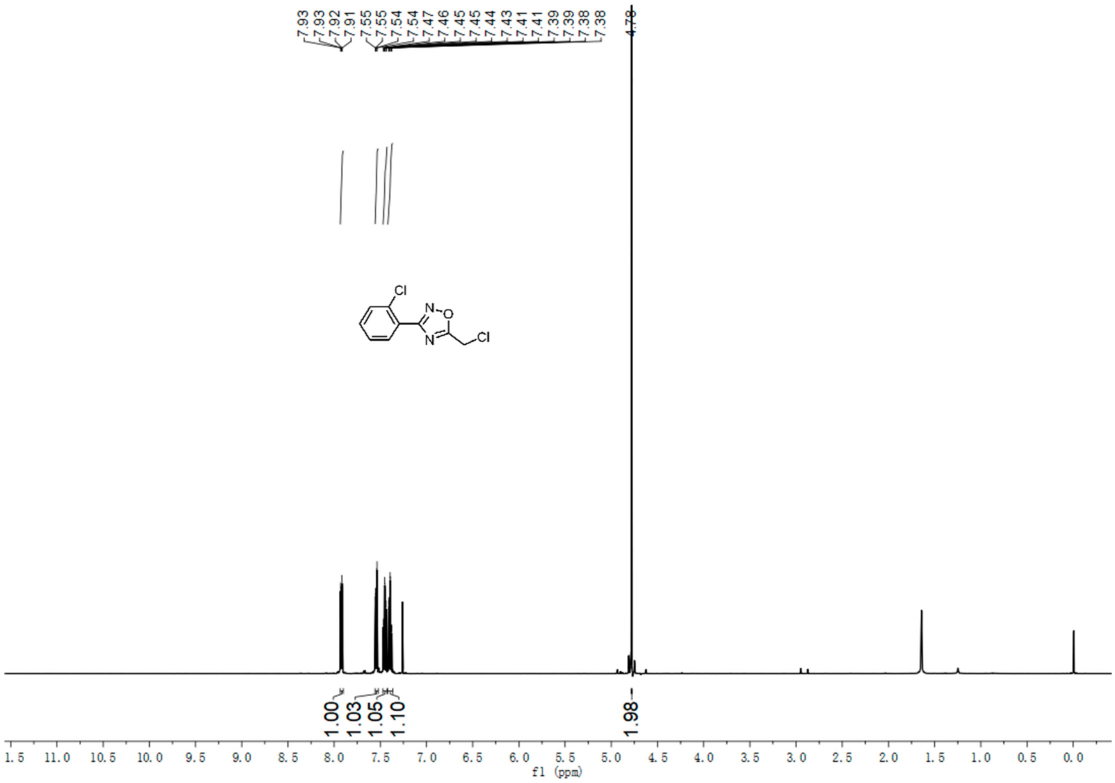

^{1}H NMR of compound **A20**

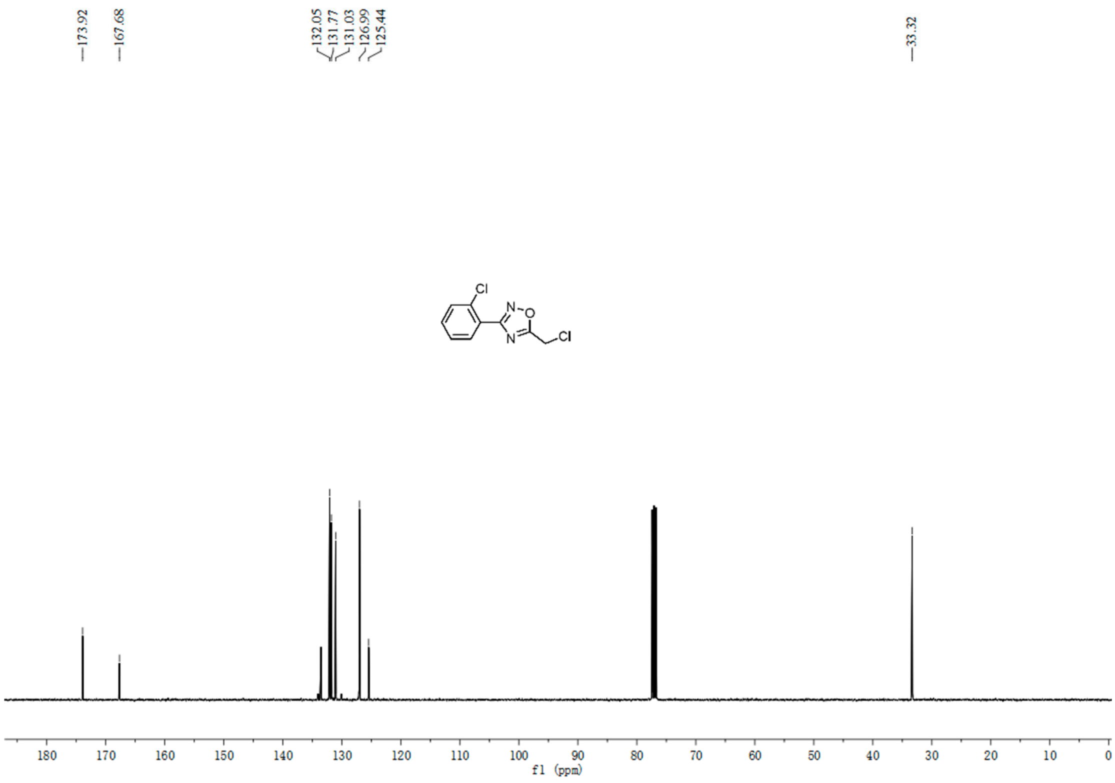

^{13}C NMR of compound **A20**

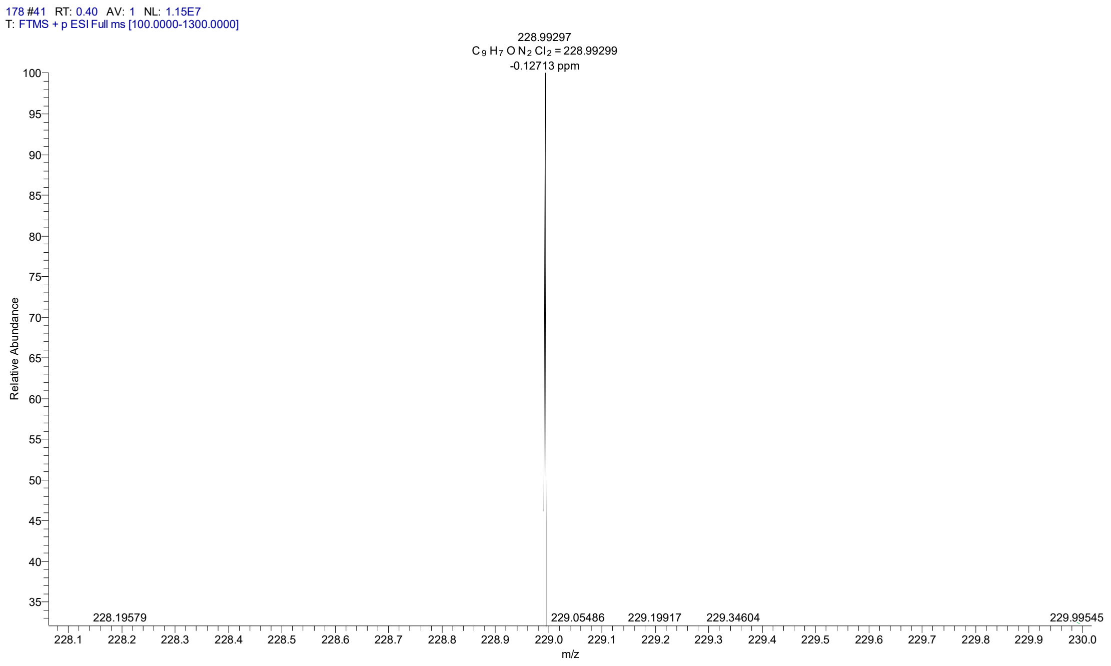

HRMS of compound **A20**

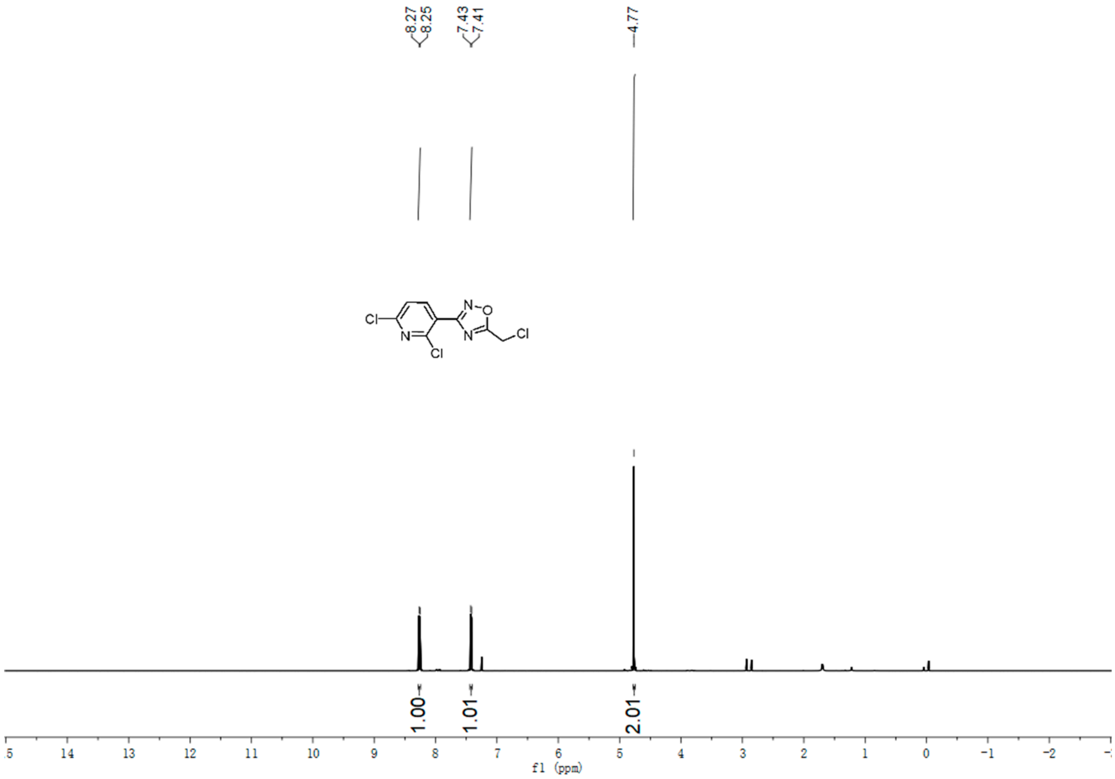

^{1}H NMR of compound **A21**

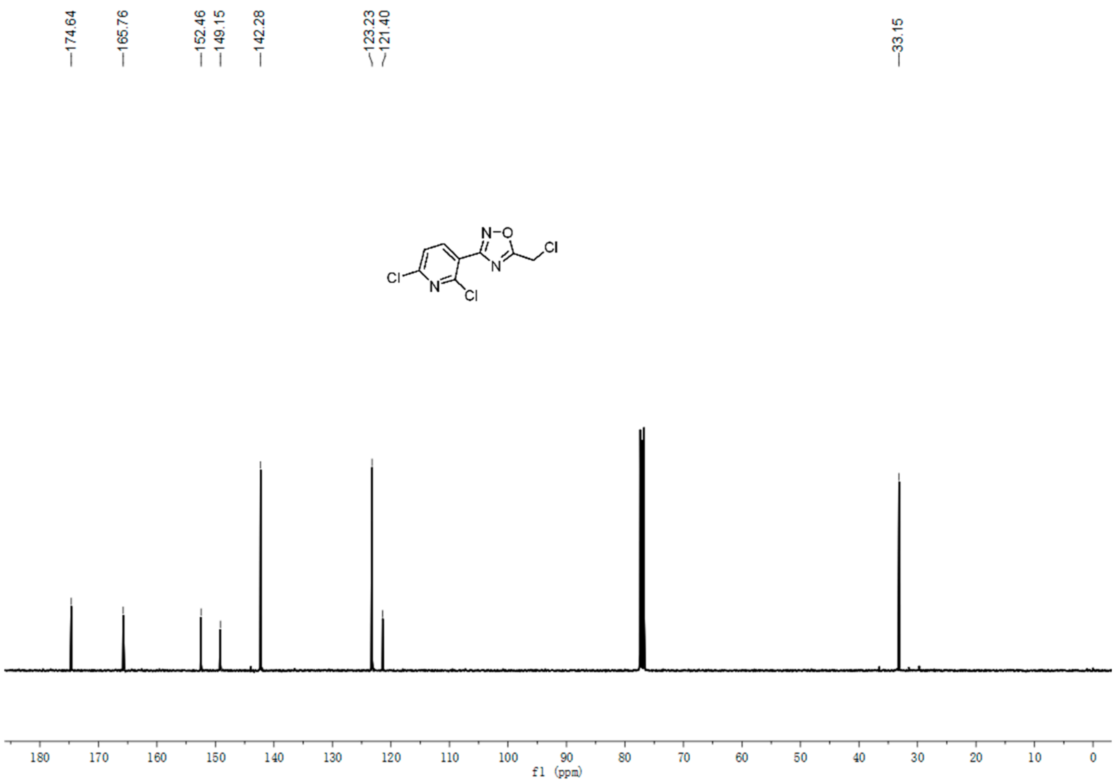

^{13}C NMR of compound **A21**

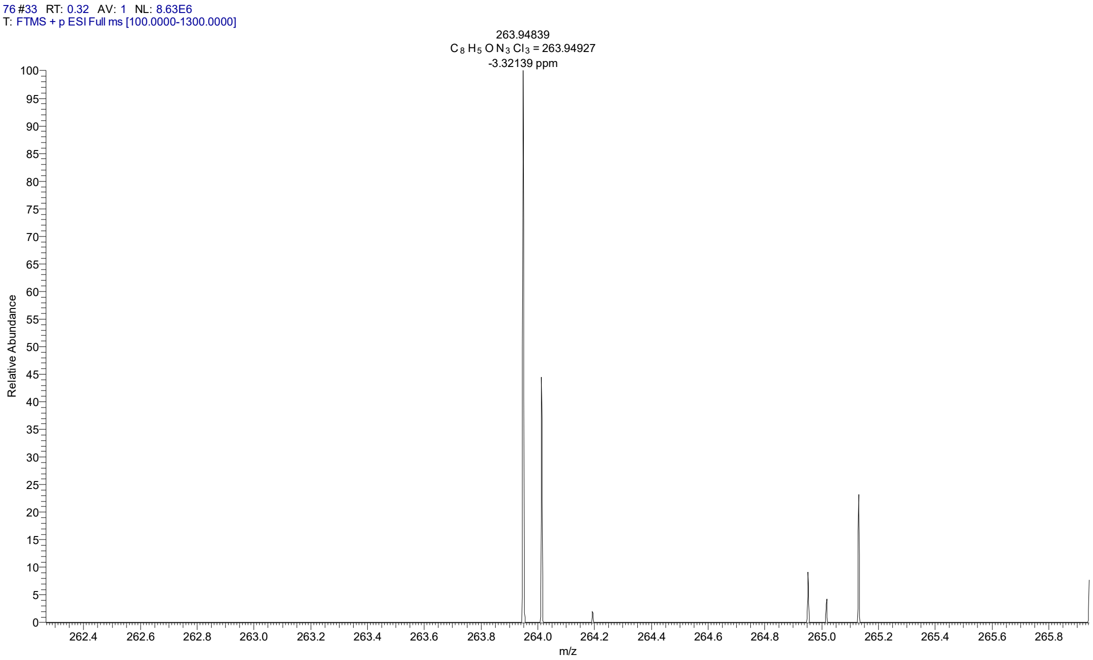

HRMS of compound **A21**

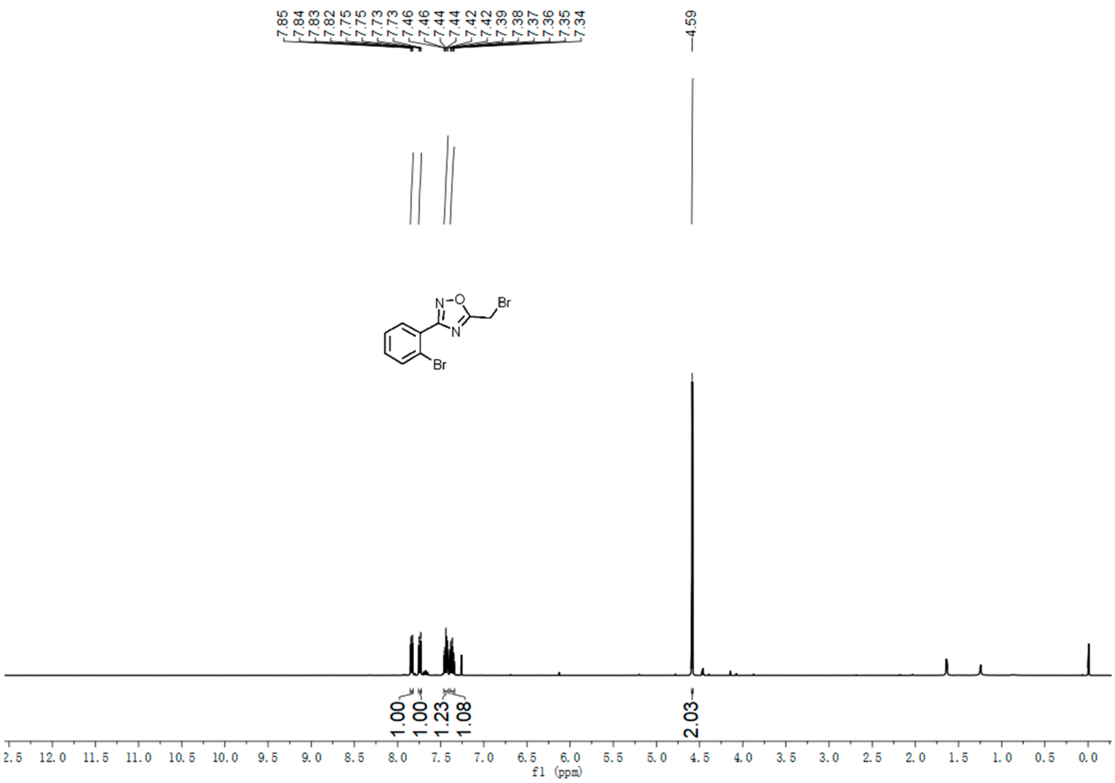

^{1}H NMR of compound **A22**

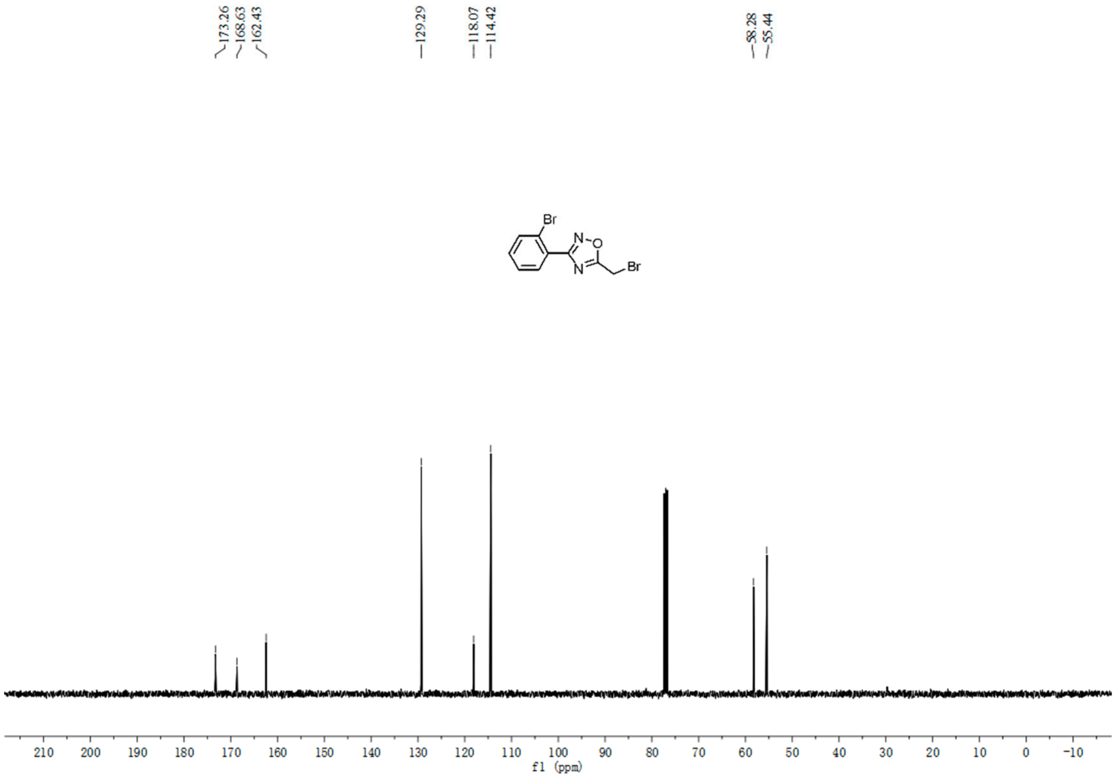

^{13}C NMR of compound **A22**

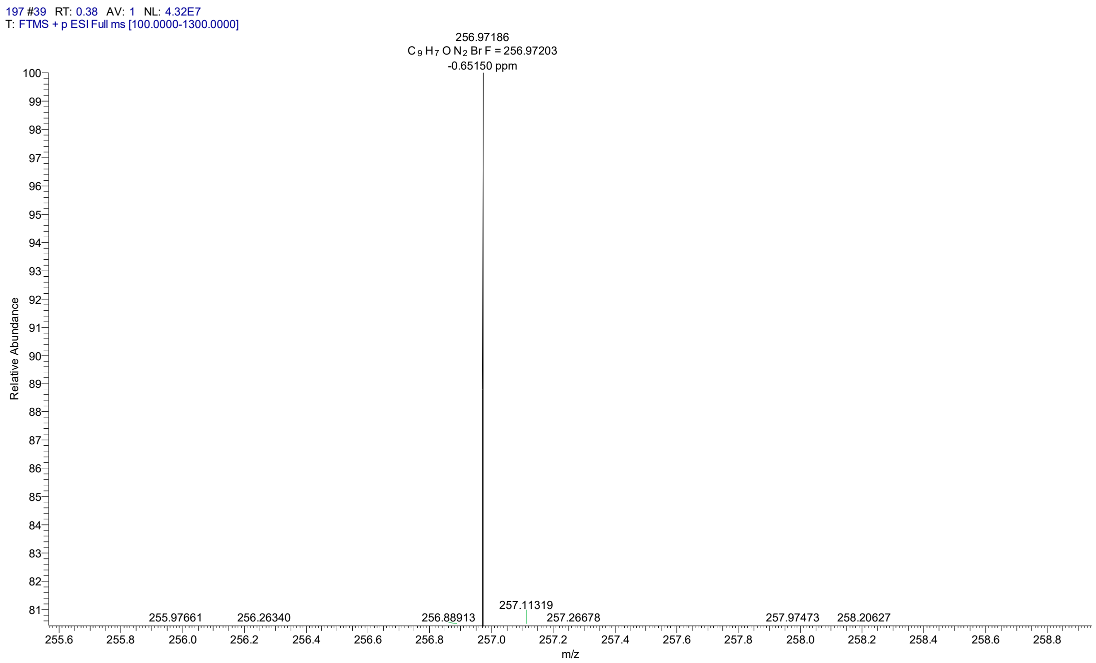

HRMS of compound **A22**

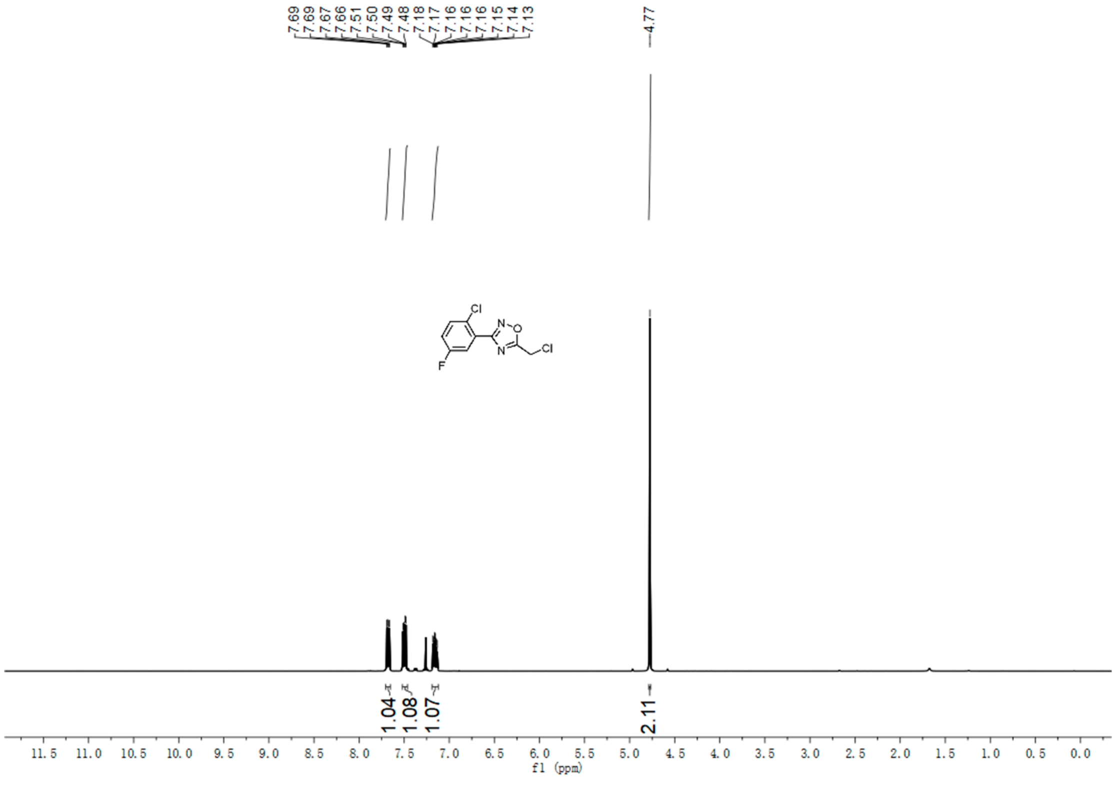

^{1}H NMR of compound **A23**

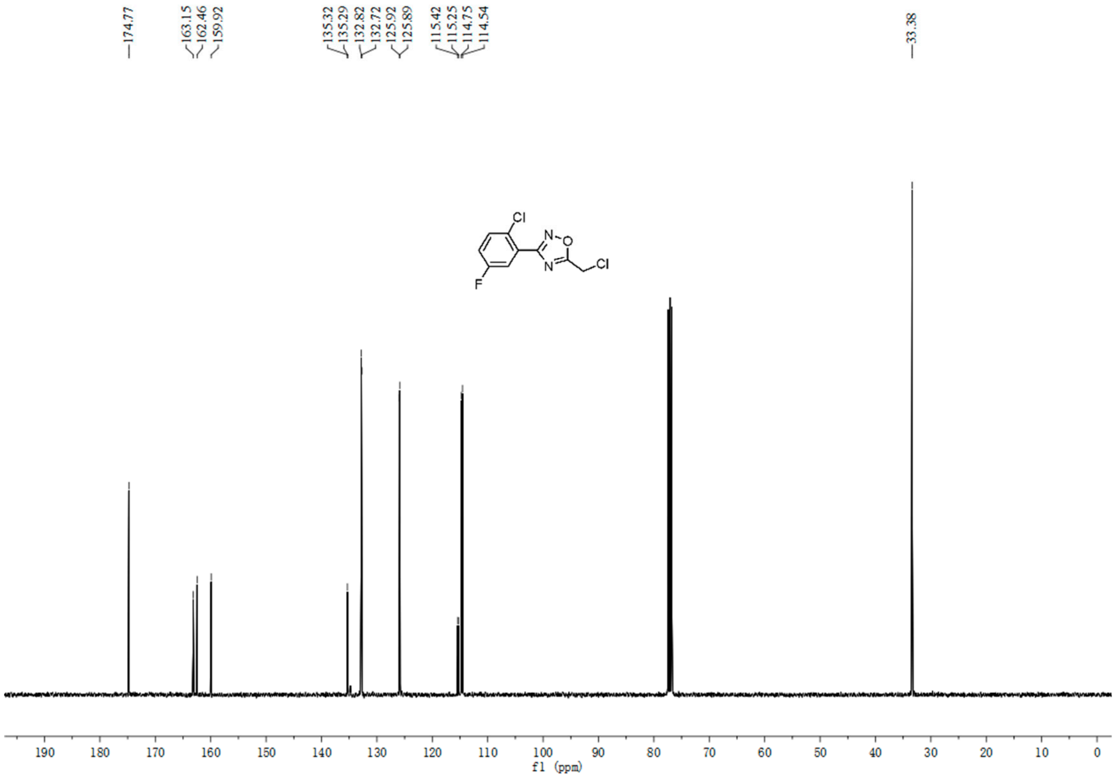

^{13}C NMR of compound **A23**

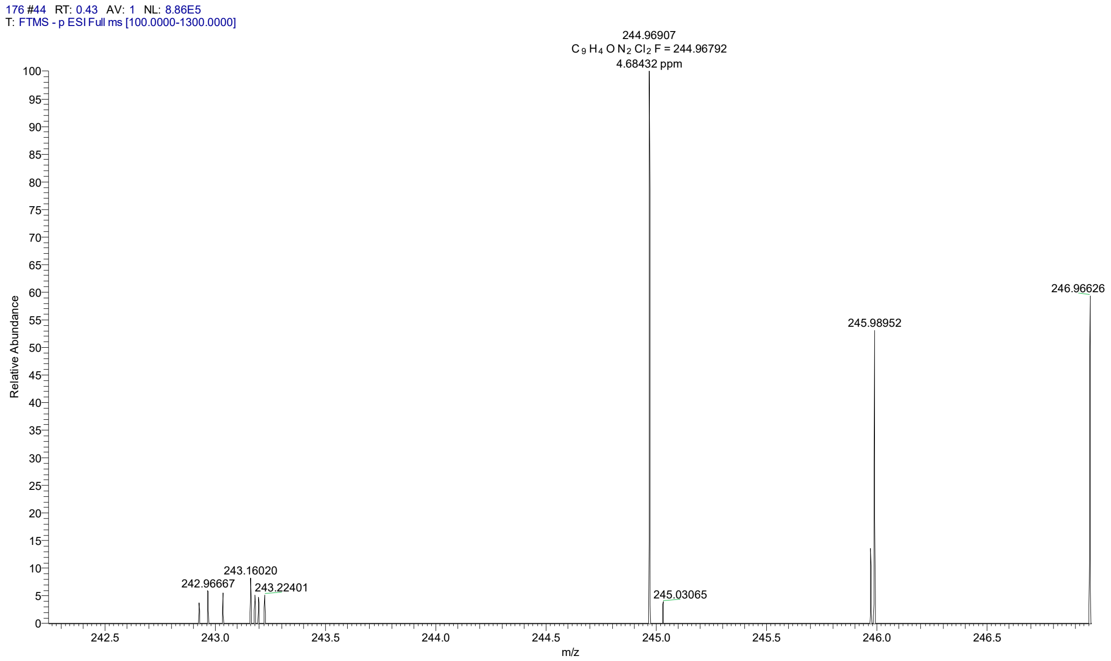

HRMS of compound **A23**

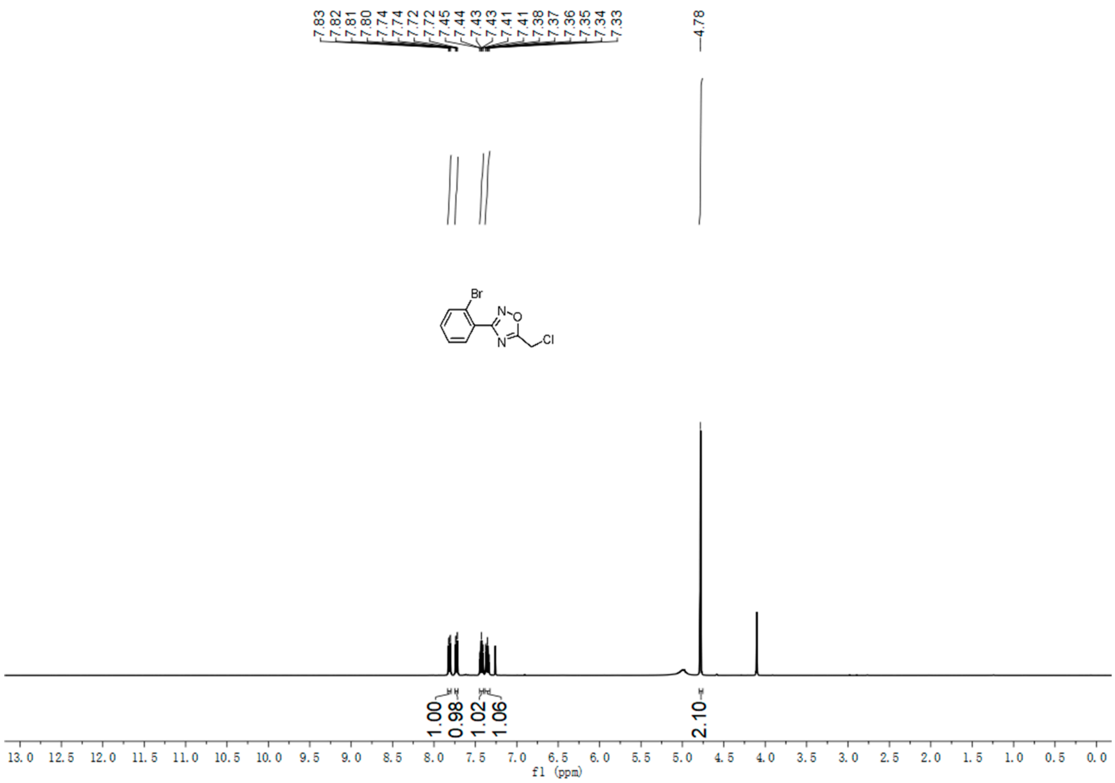

^{1}H NMR of compound **A24**

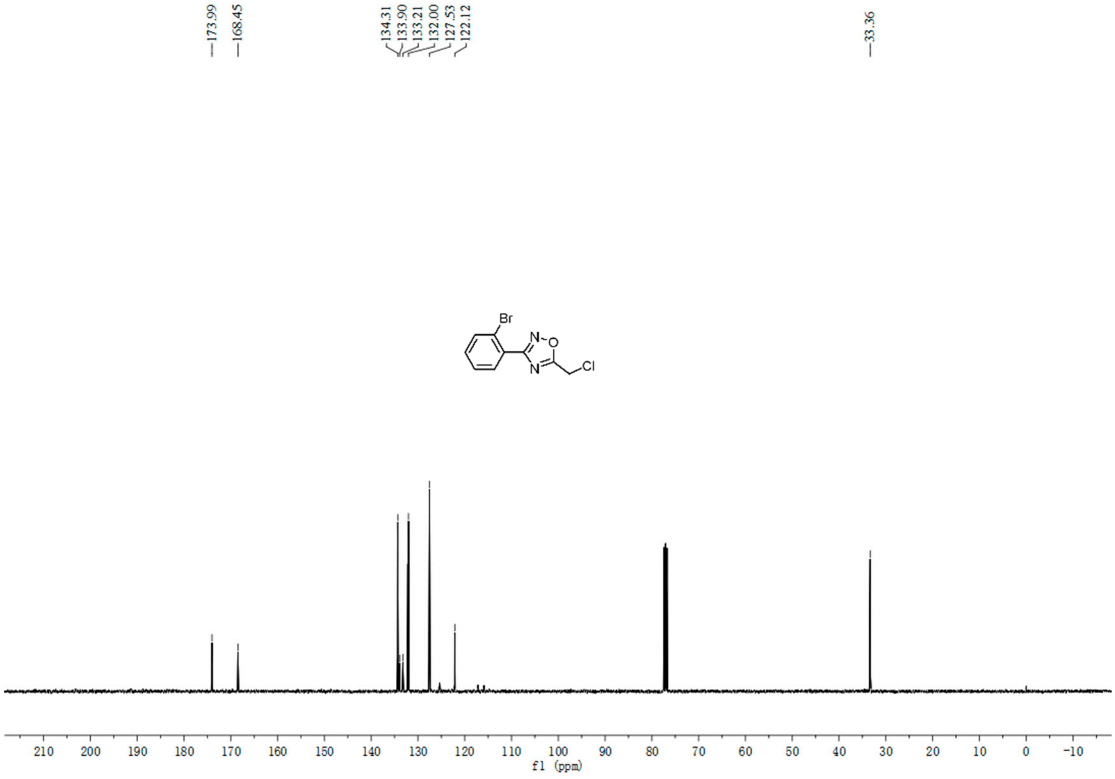

^{13}C NMR of compound **A24**

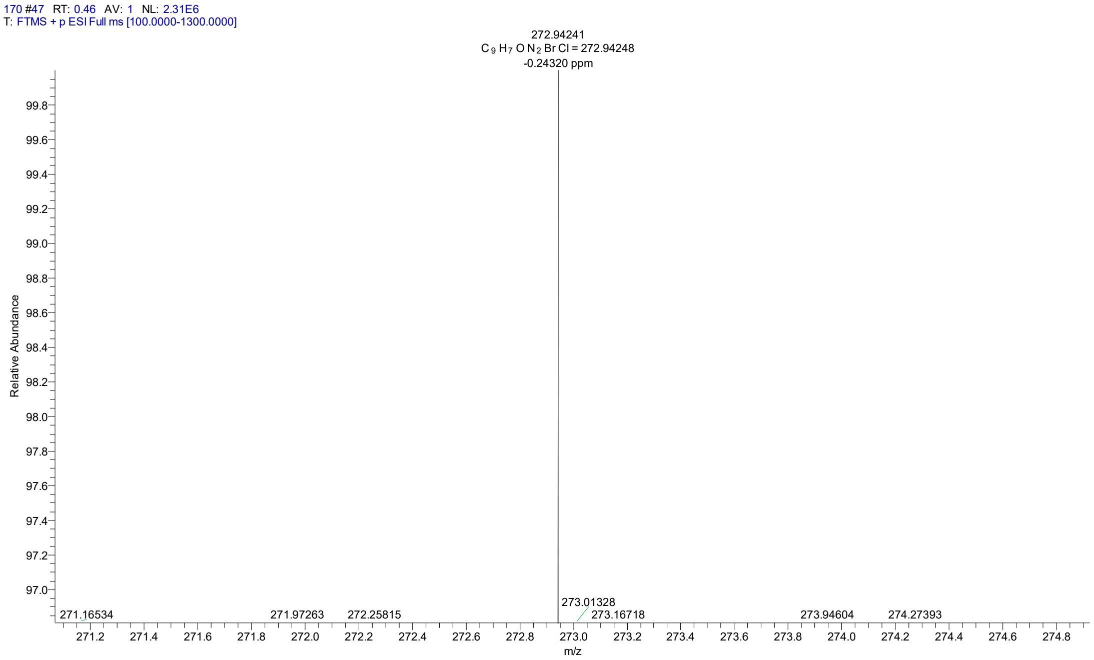

HRMS of compound **A24**

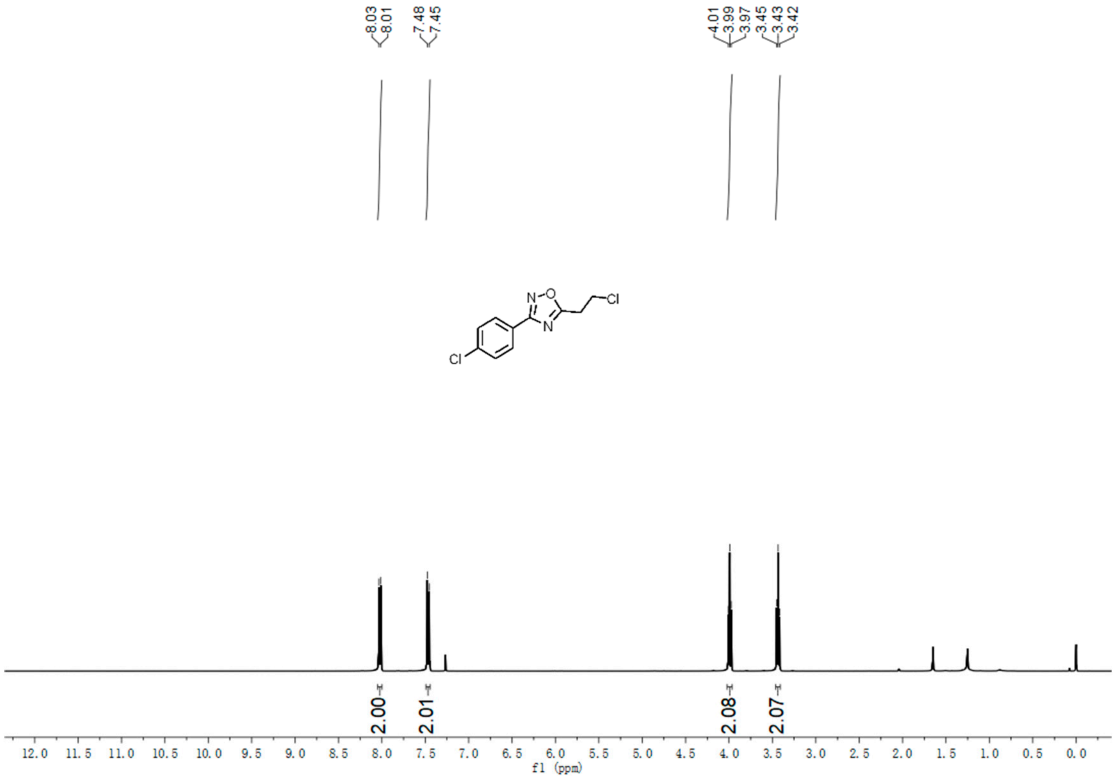

^{1}H NMR of compound **A25**

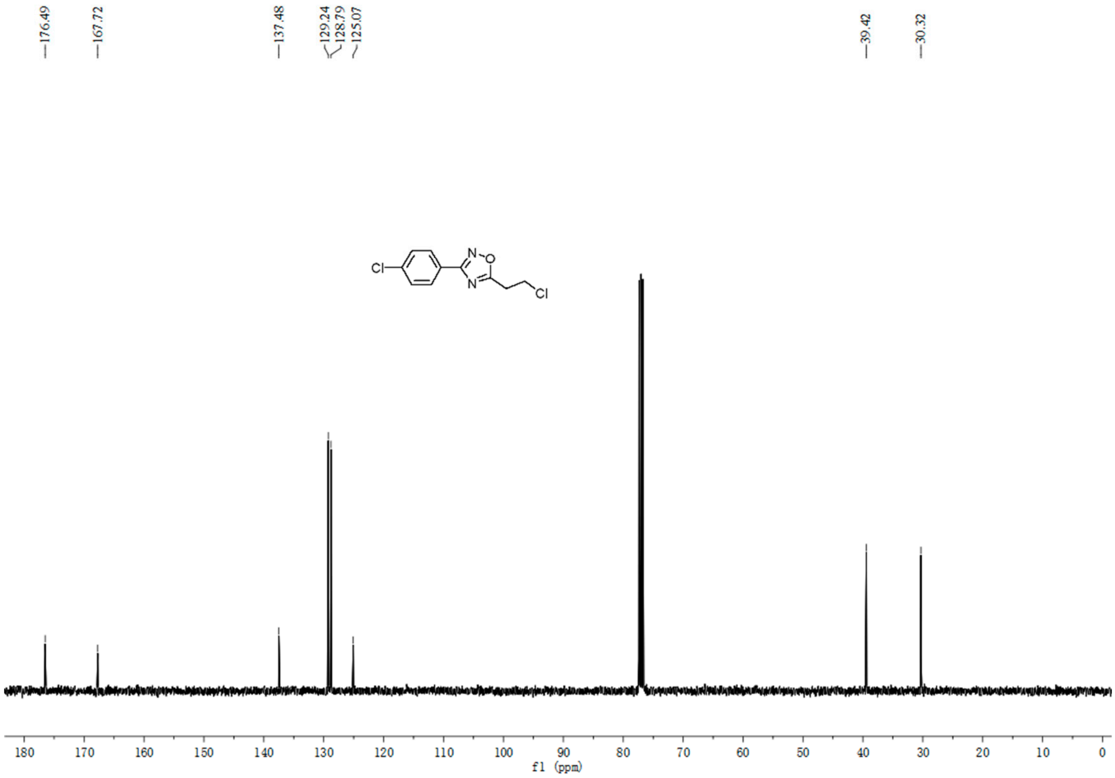

^{13}C NMR of compound **A25**

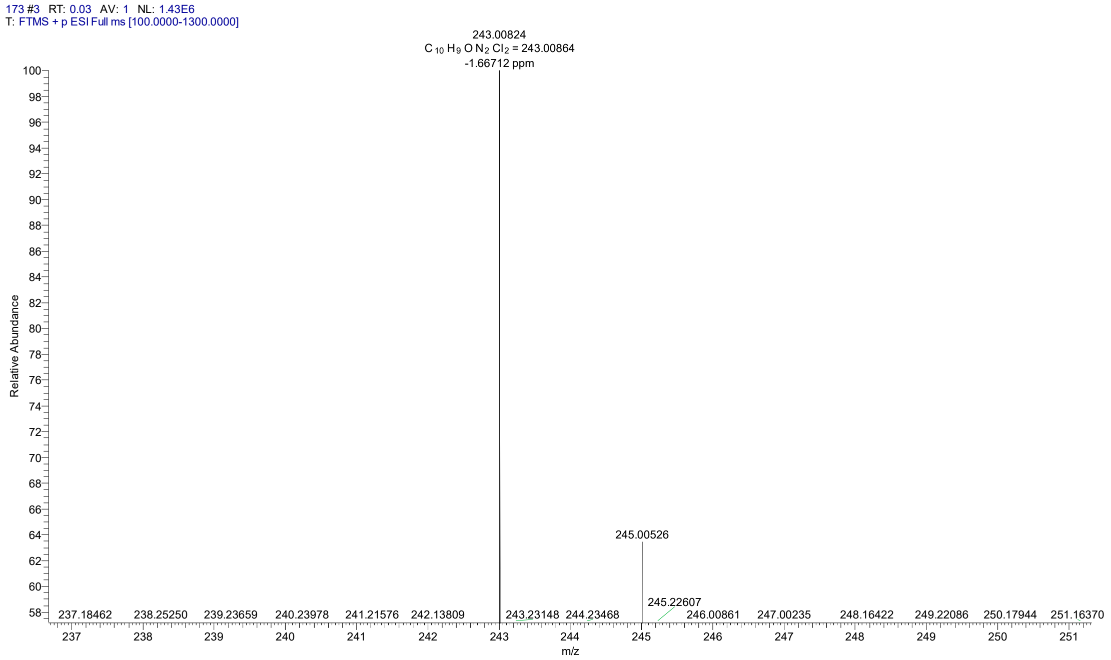

HRMS of compound **A25**

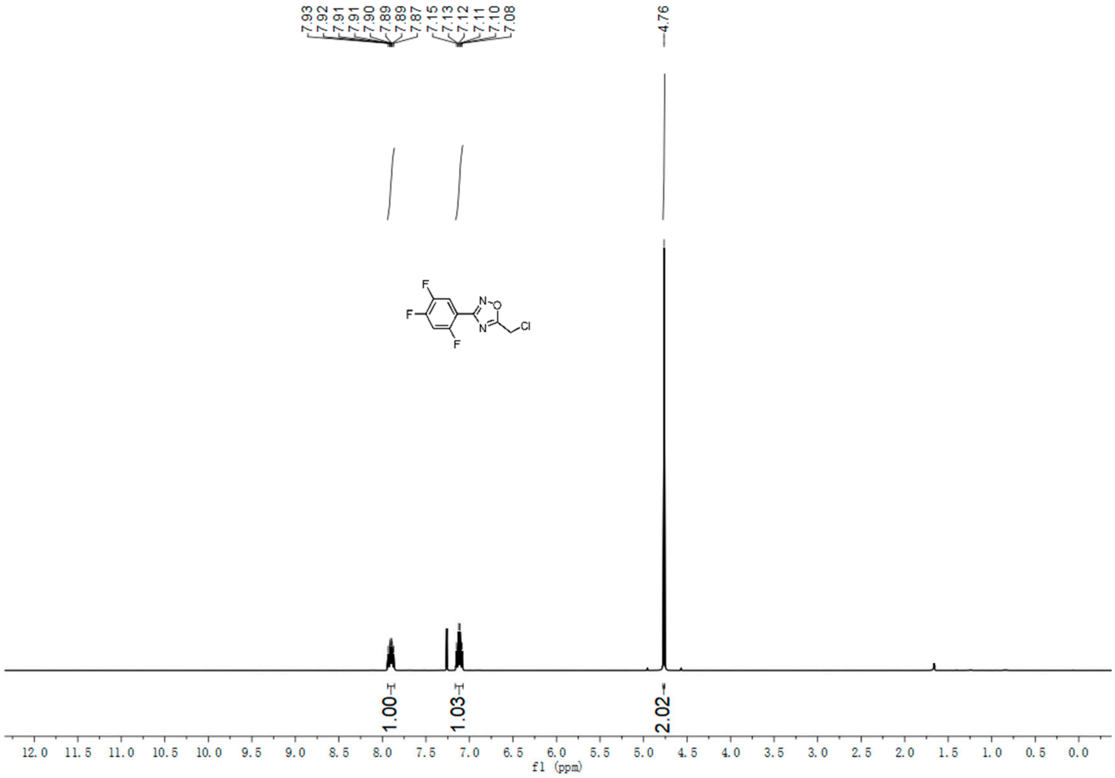

^{1}H NMR of compound **A26**

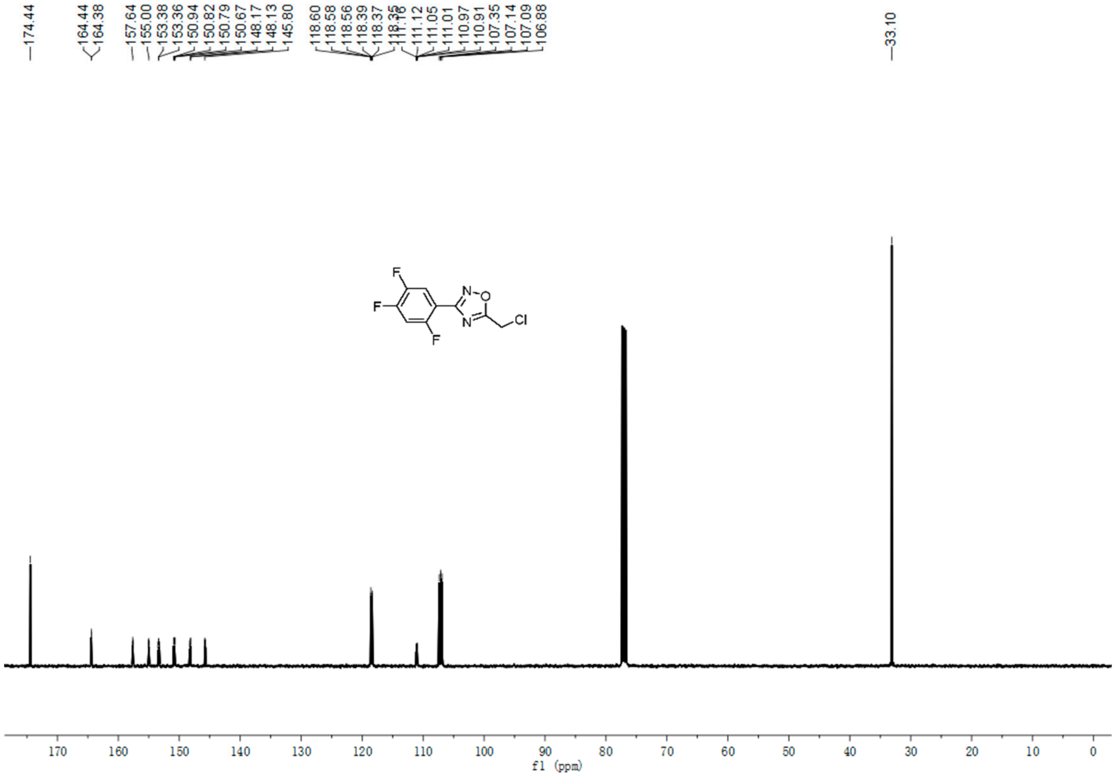

^{13}C NMR of compound **A26**

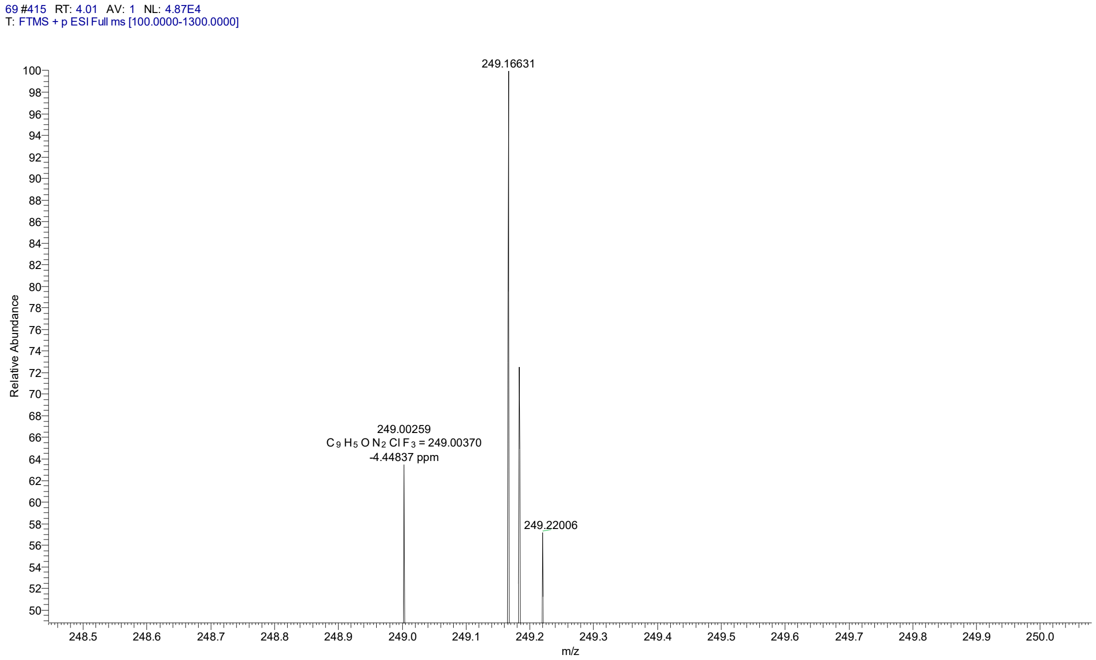

HRMS of compound **A26**

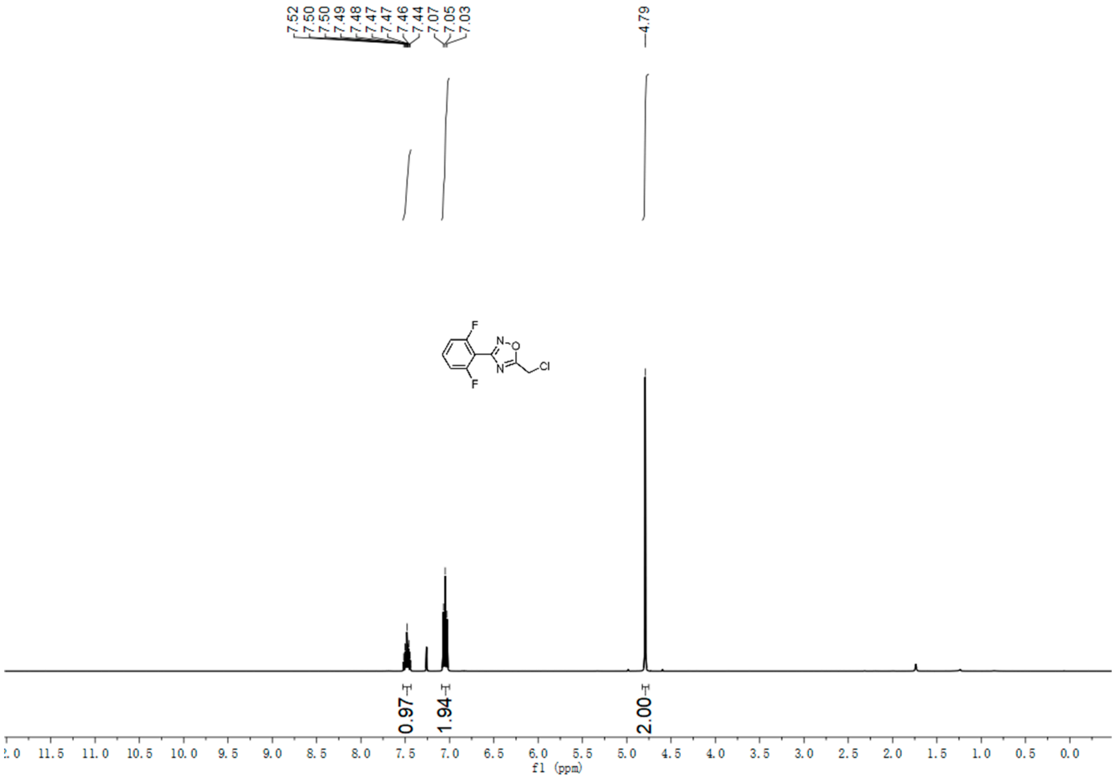

^{1}H NMR of compound **A27**

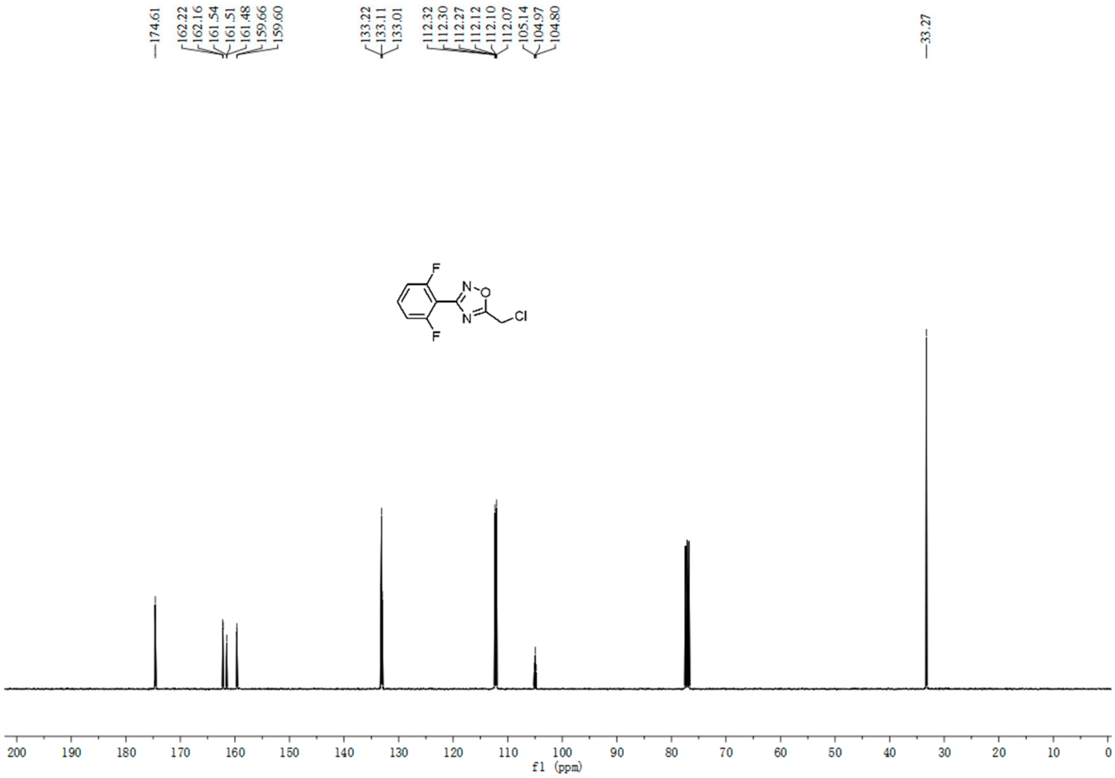

^{13}C NMR of compound **A27**

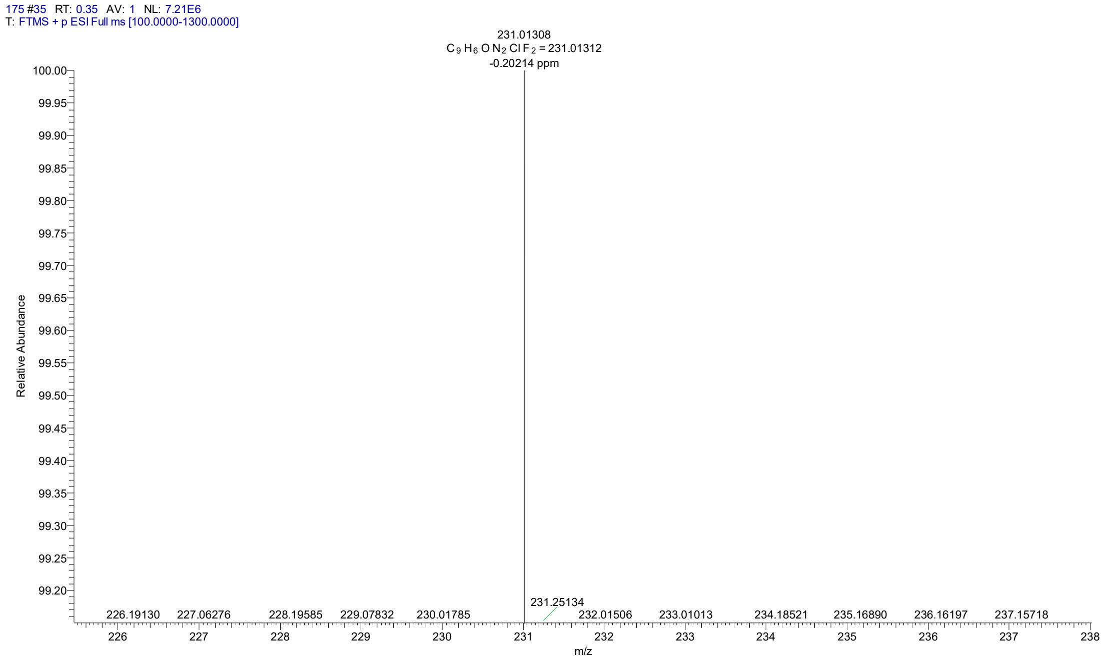

HRMS of compound **A27**

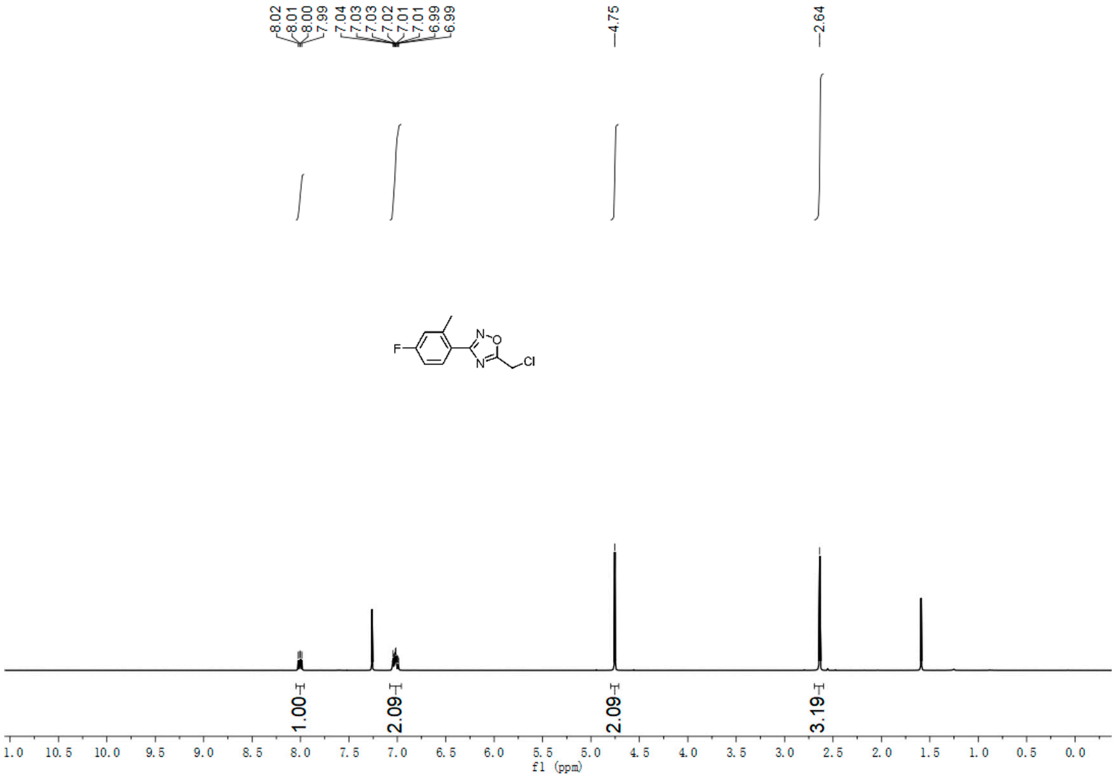

^{1}H NMR of compound **A28**

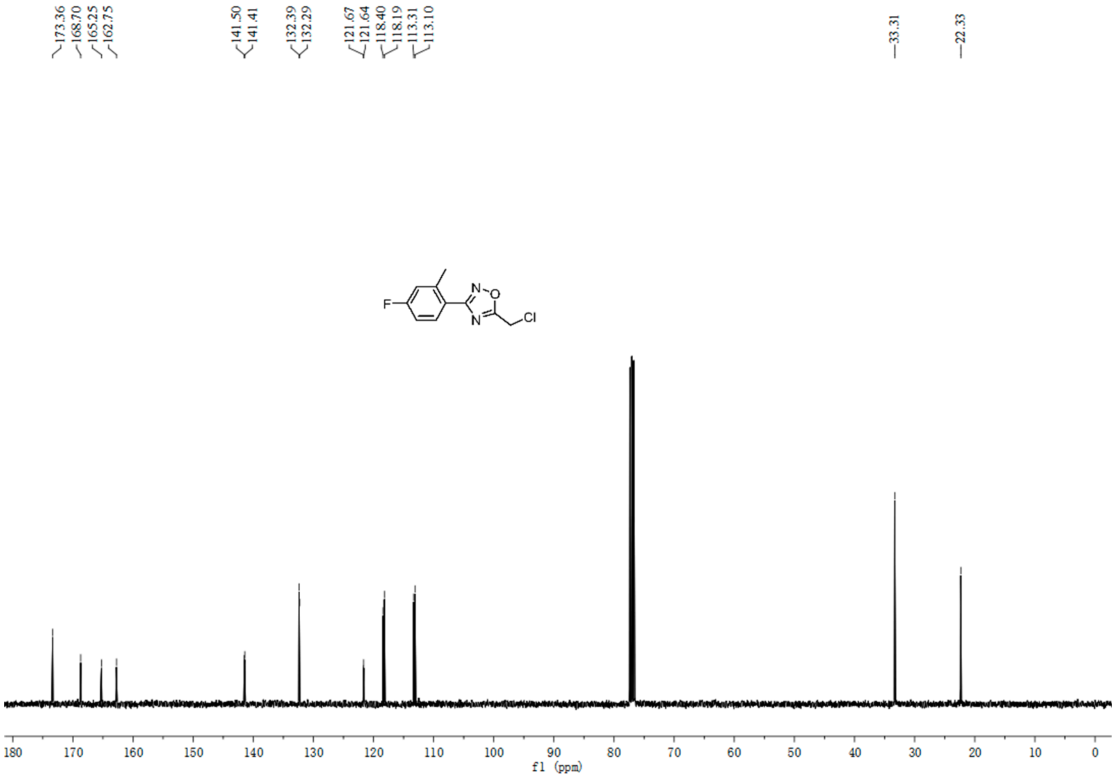

^{13}C NMR of compound **A28**

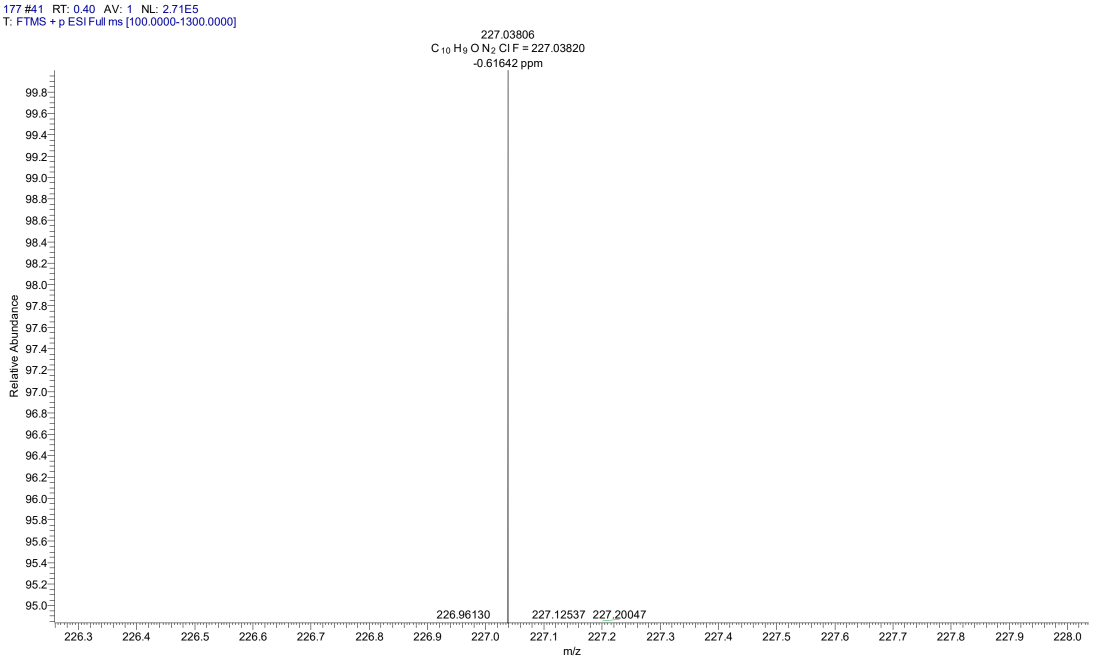

HRMS of compound **A28**

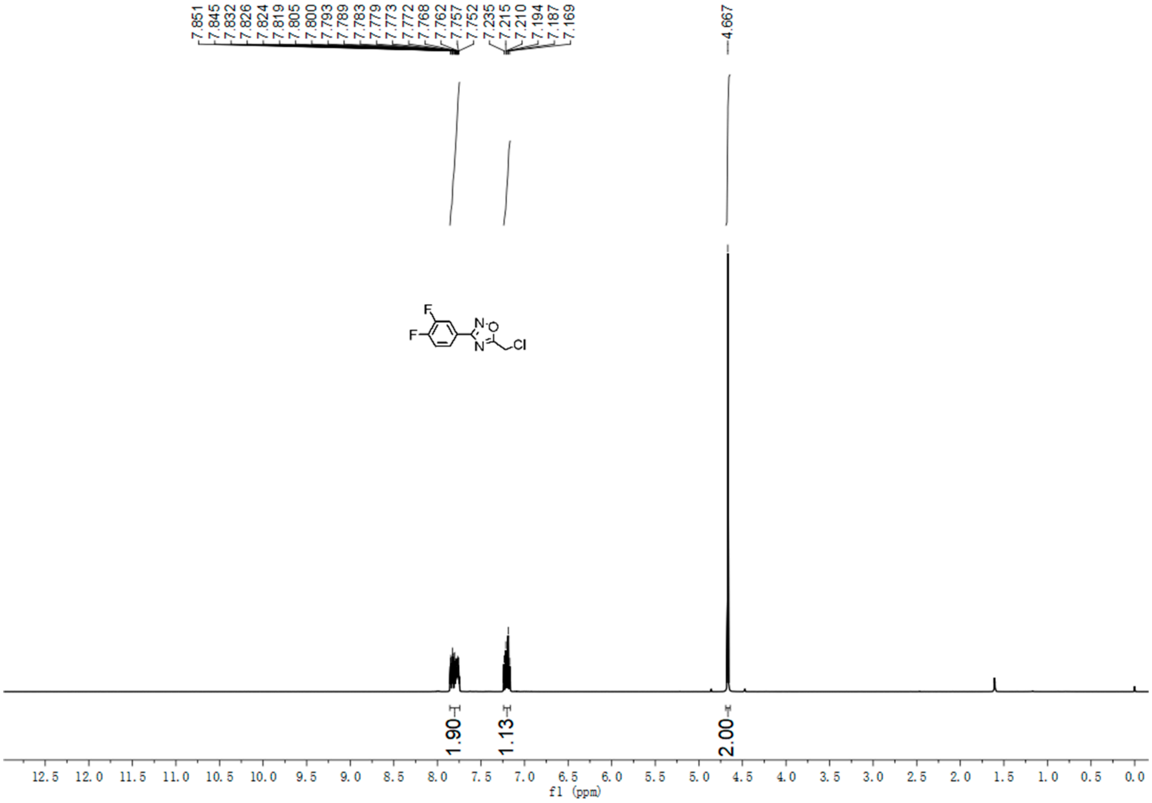

^{1}H NMR of compound **A29**

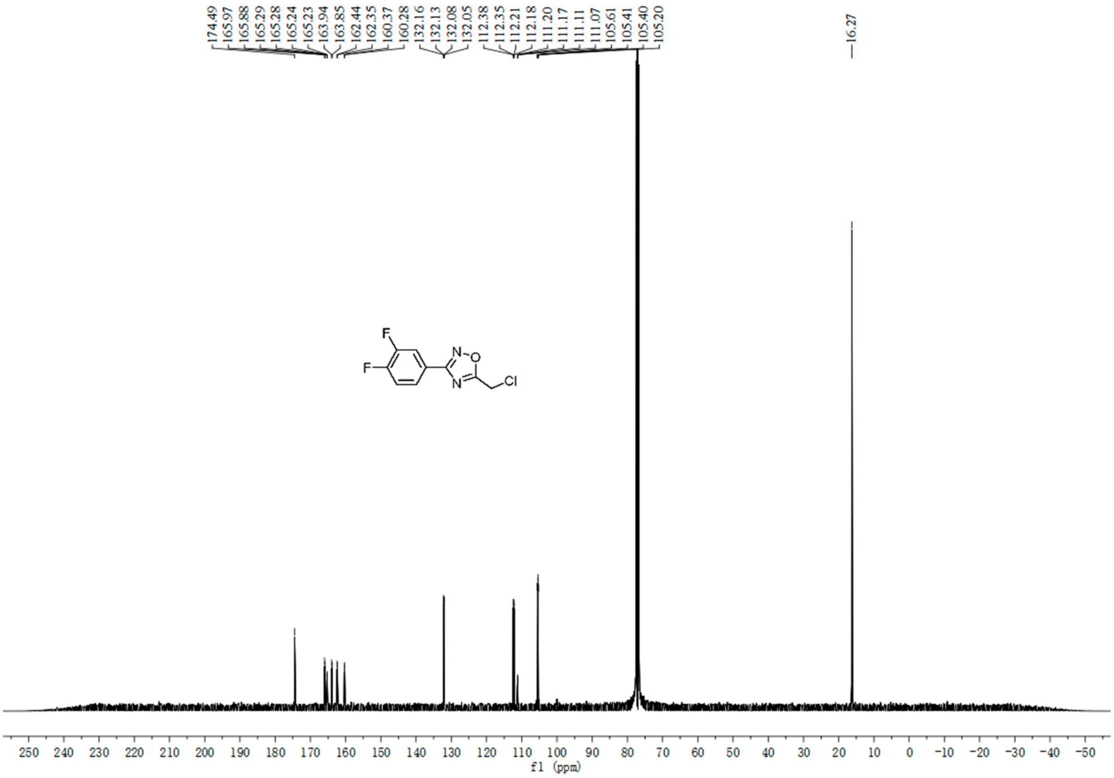

^{13}C NMR of compound **A29**

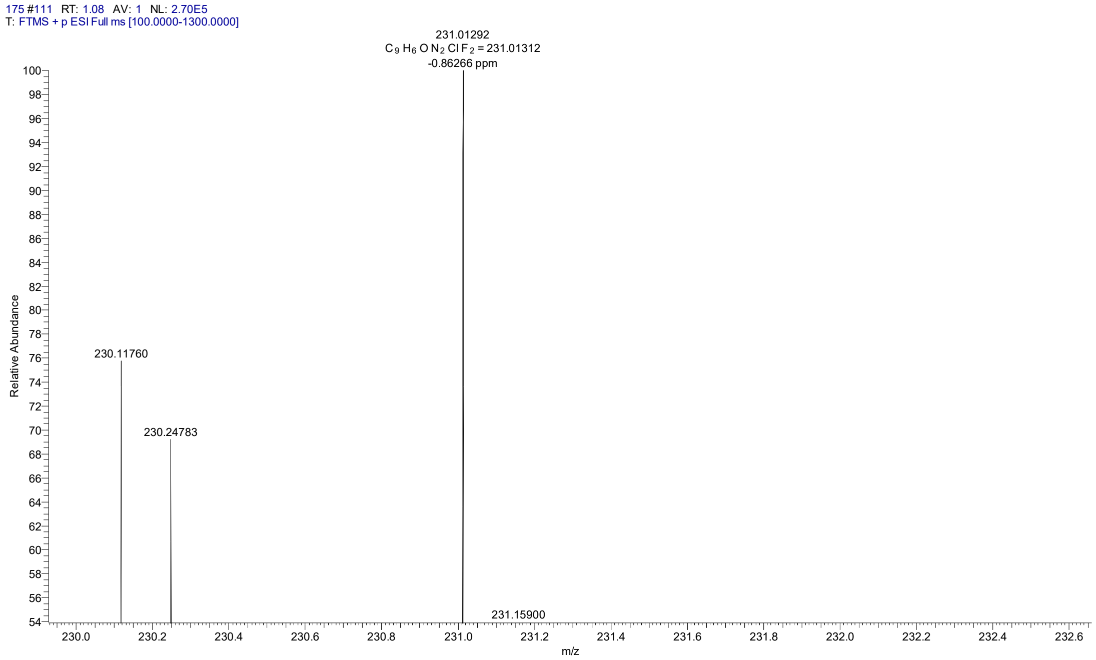

HRMS of compound **A29**

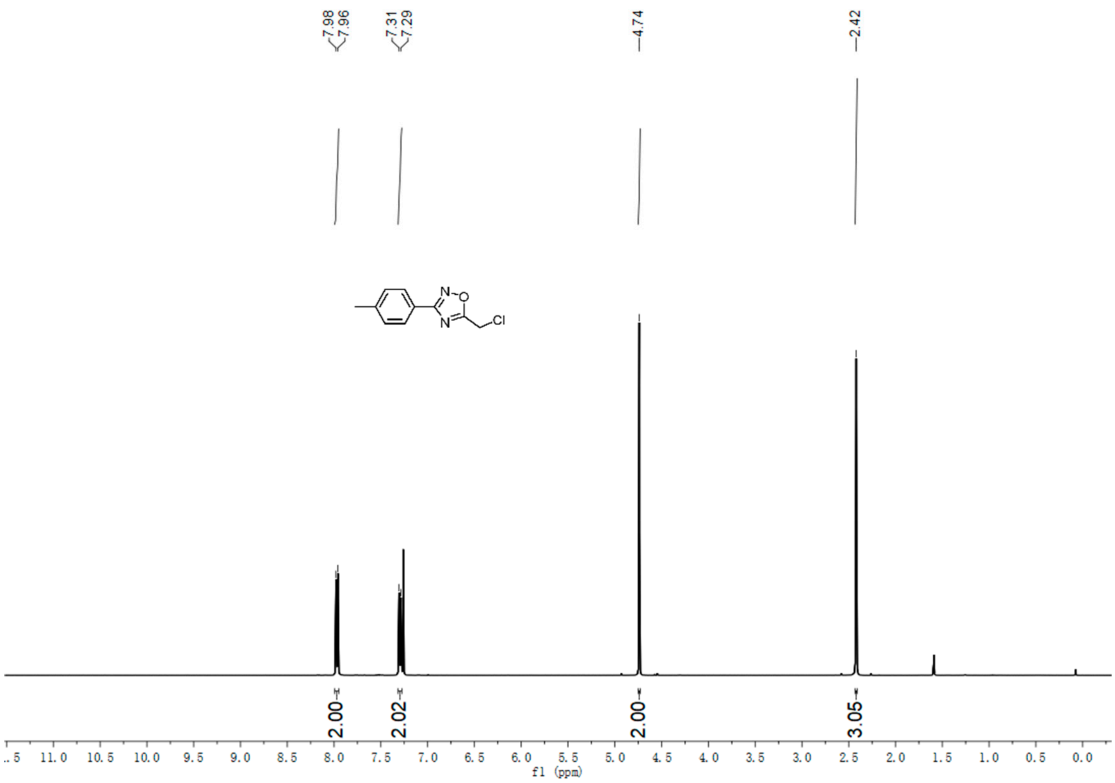

^{1}H NMR of compound **B1**

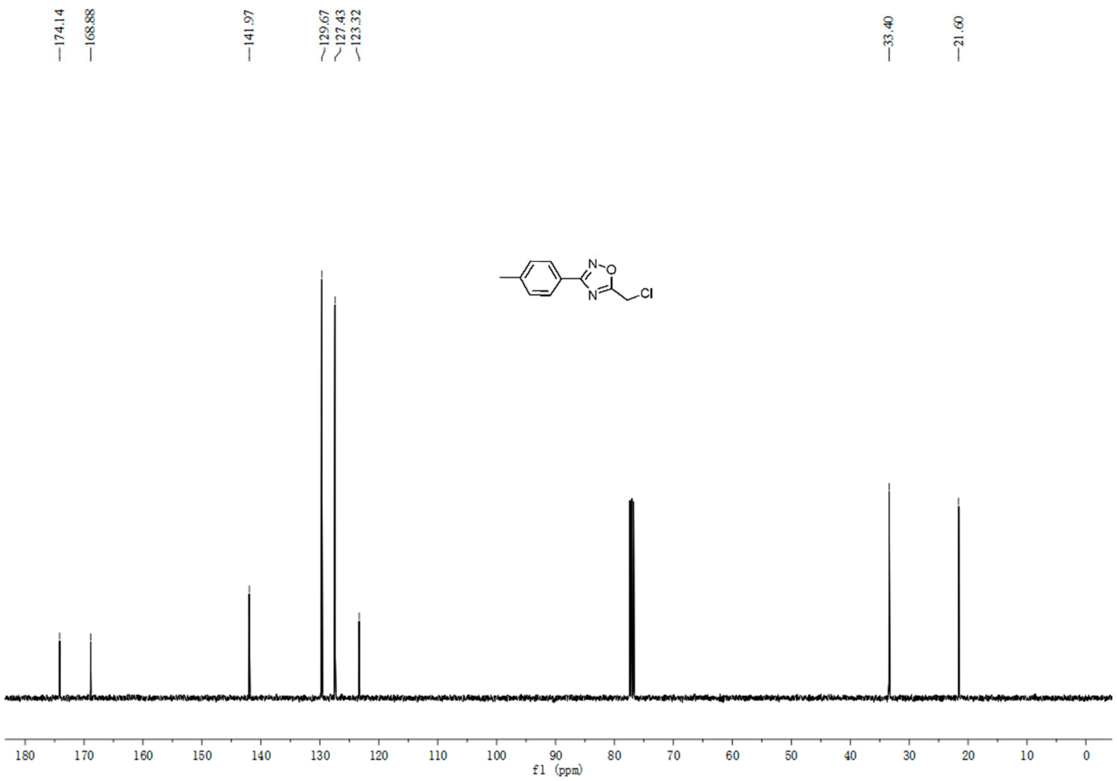

^{13}C NMR of compound **B1**

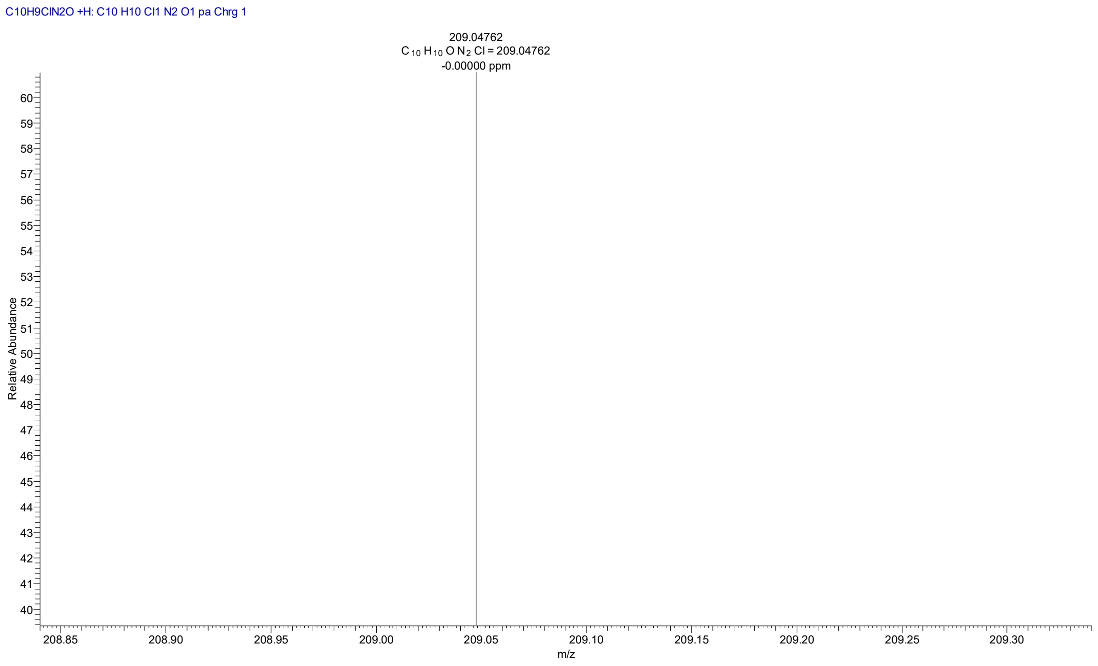

HRMS of compound **B1**

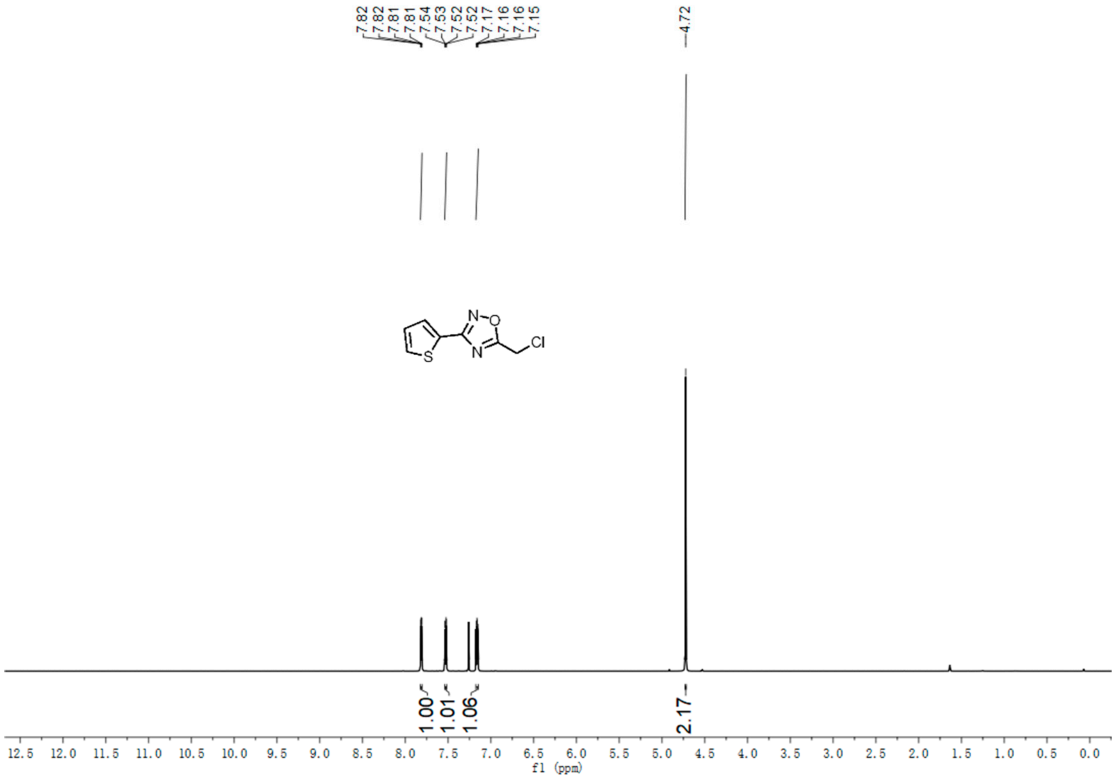

^{1}H NMR of compound **B2**

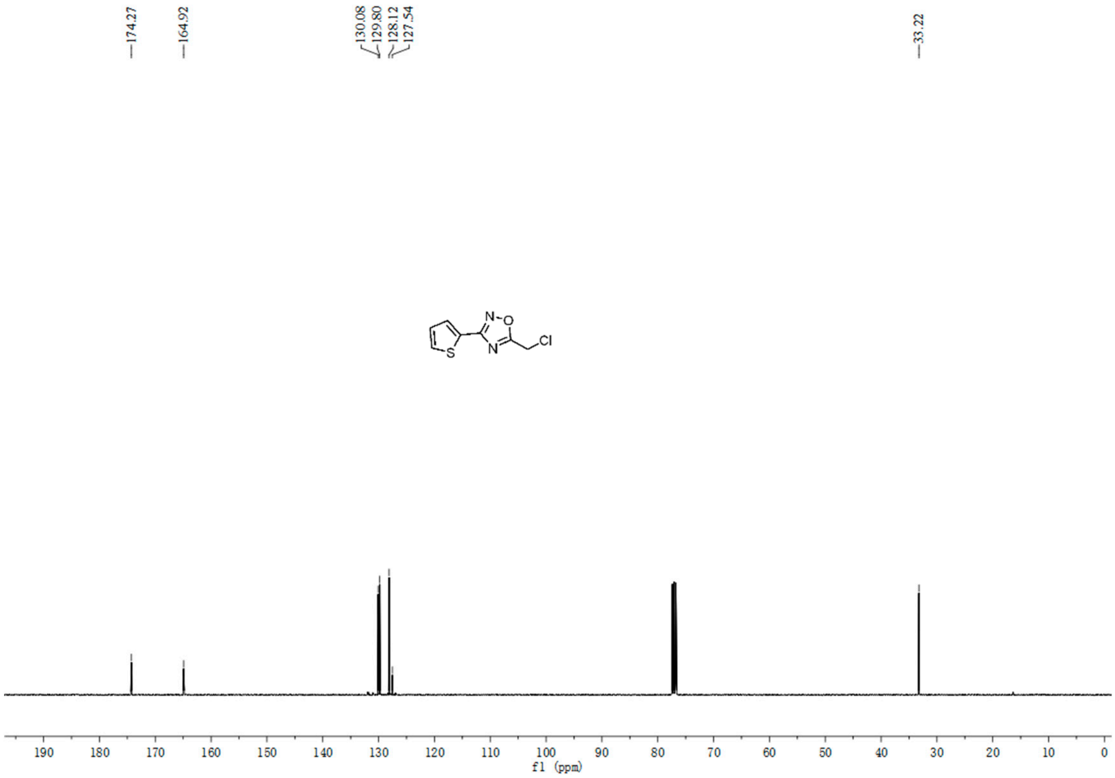

^{13}C NMR of compound **B2**

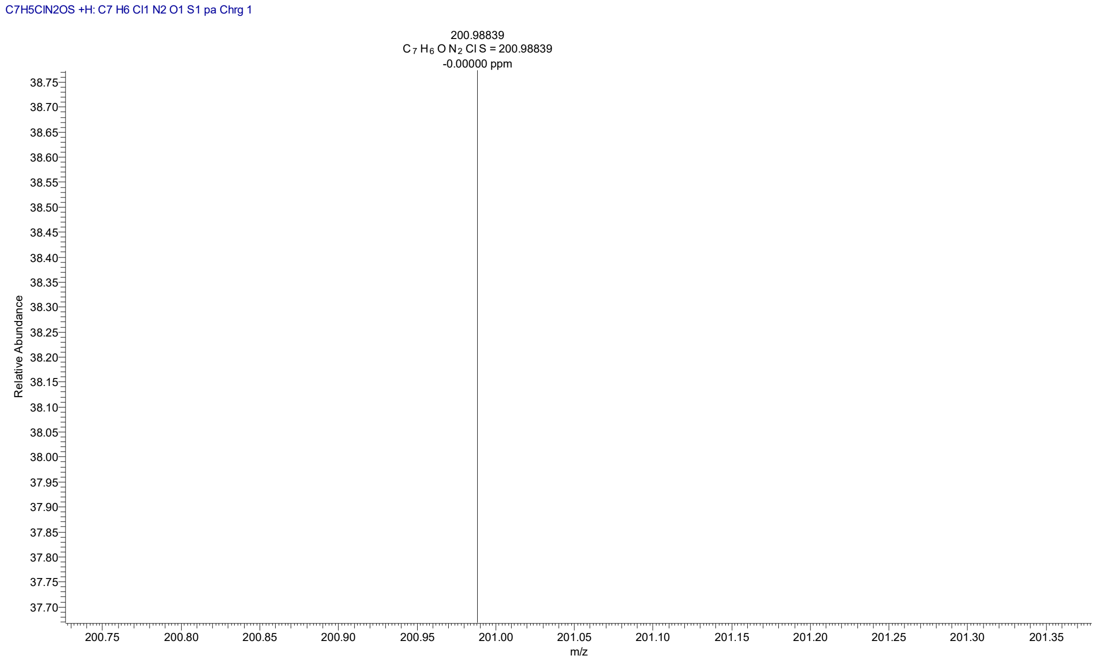

HRMS of compound **B2**

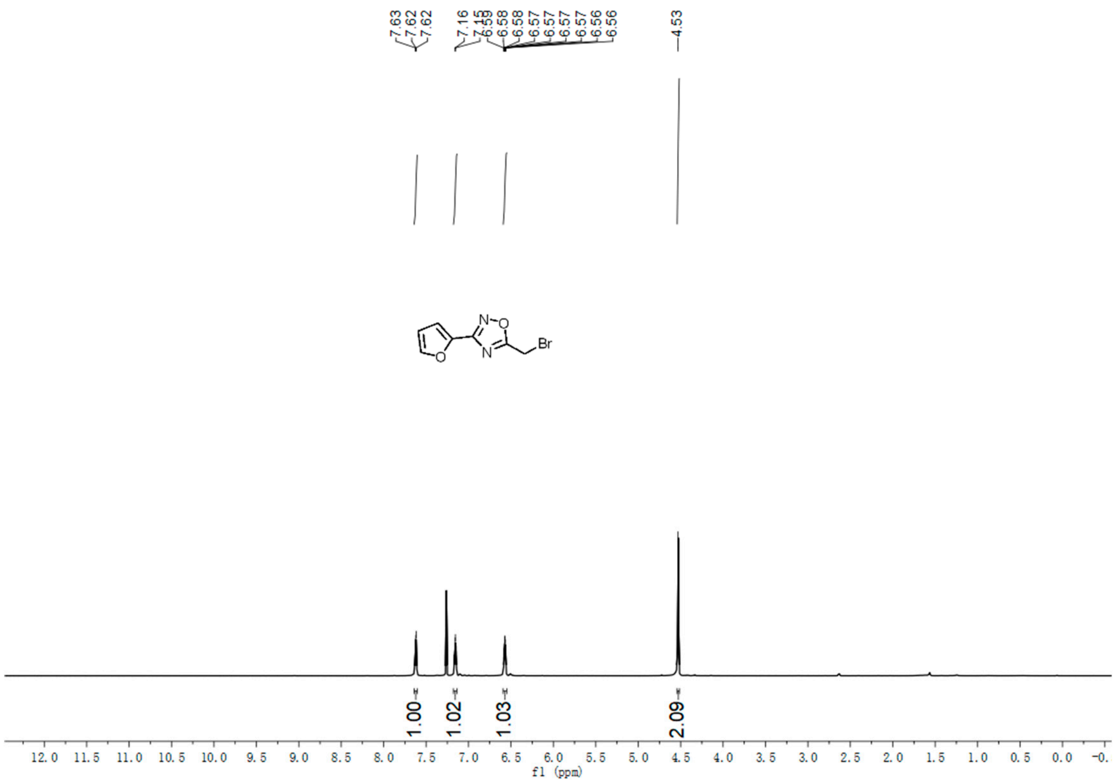

^{1}H NMR of compound **B3**

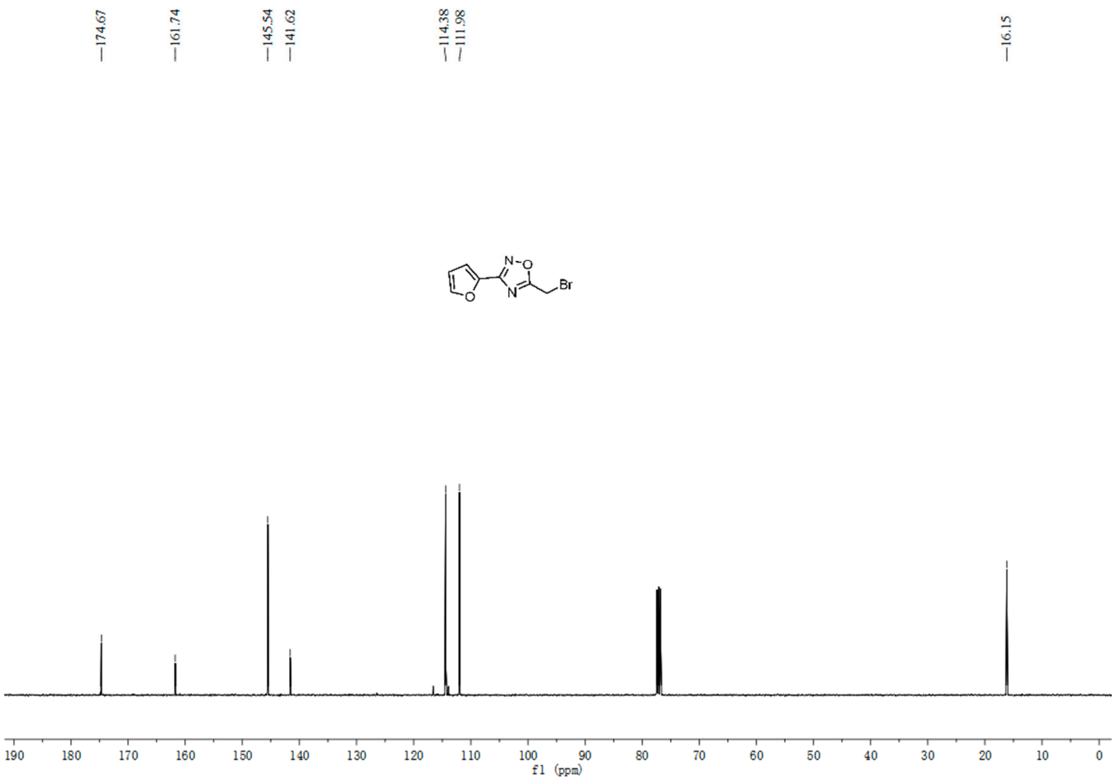

^{13}C NMR of compound **B3**

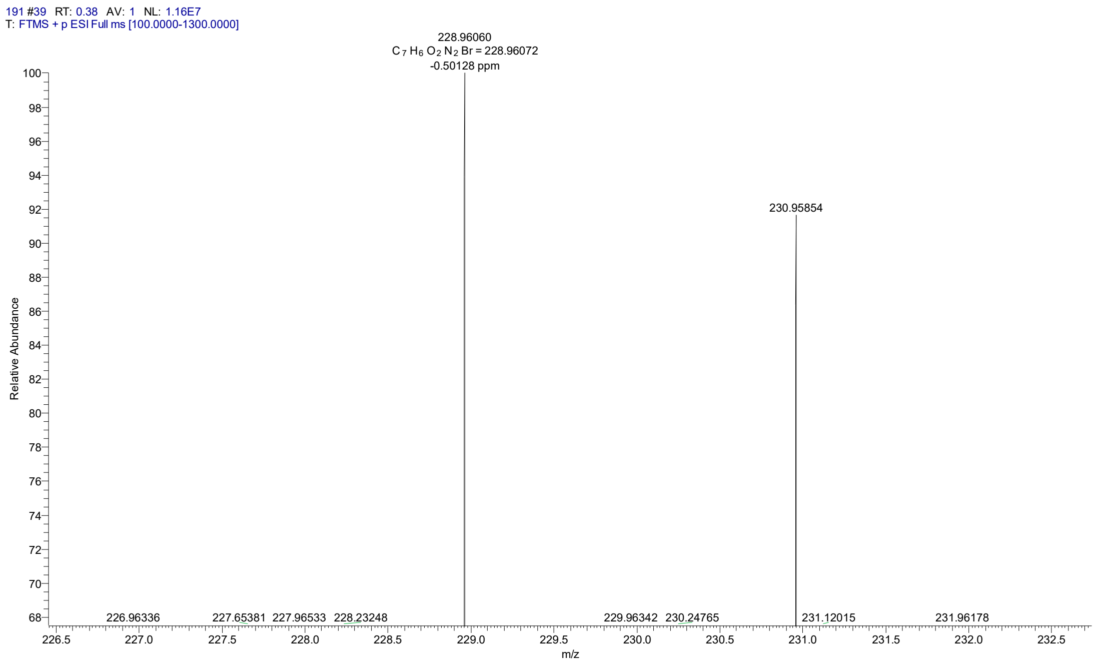

HRMS of compound **B3**

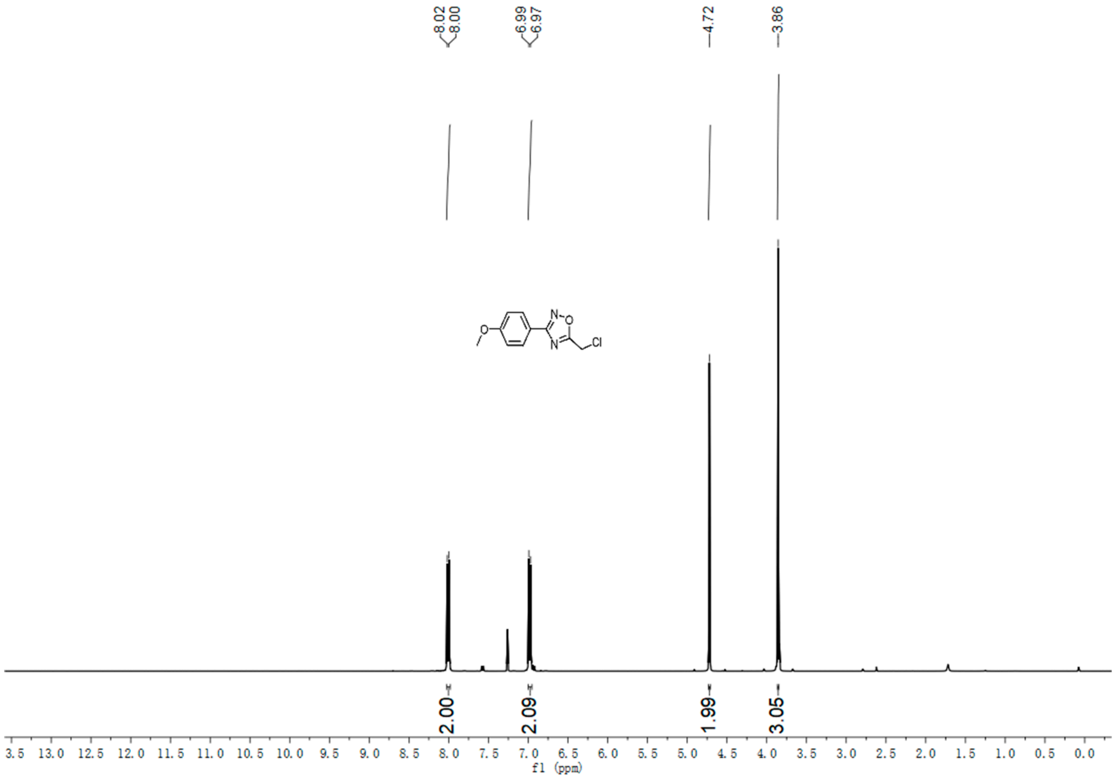

^{1}H NMR of compound **B4**

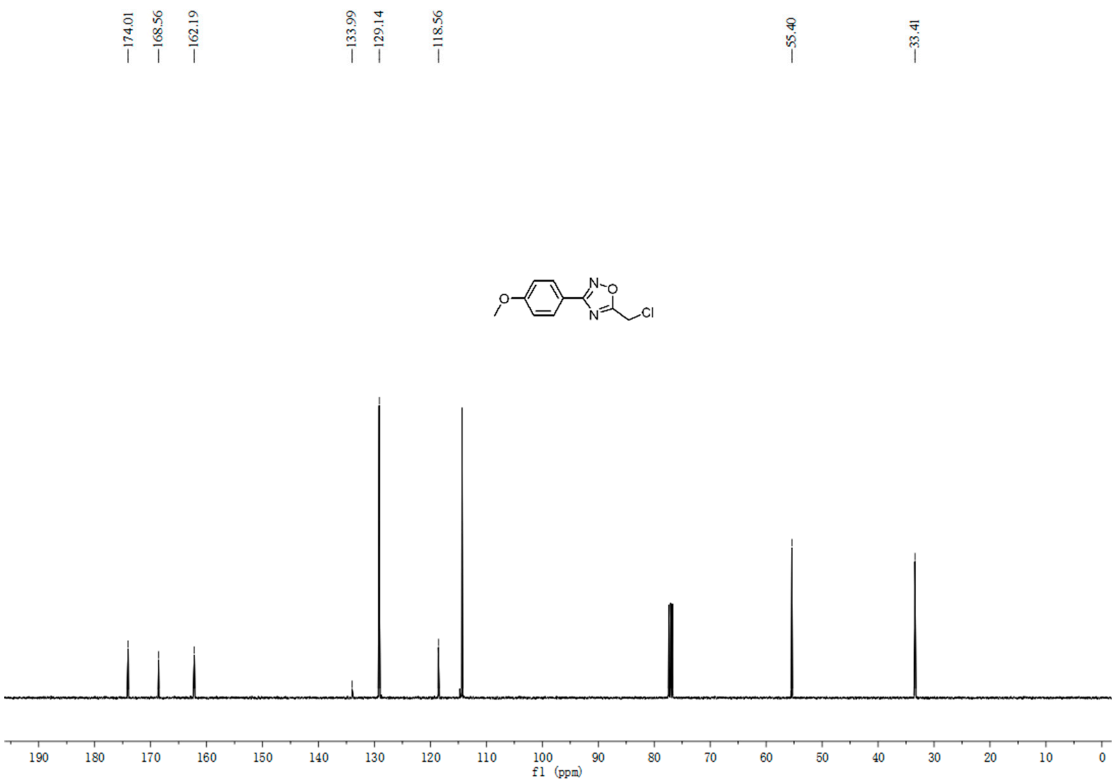

^{13}C NMR of compound **B4**

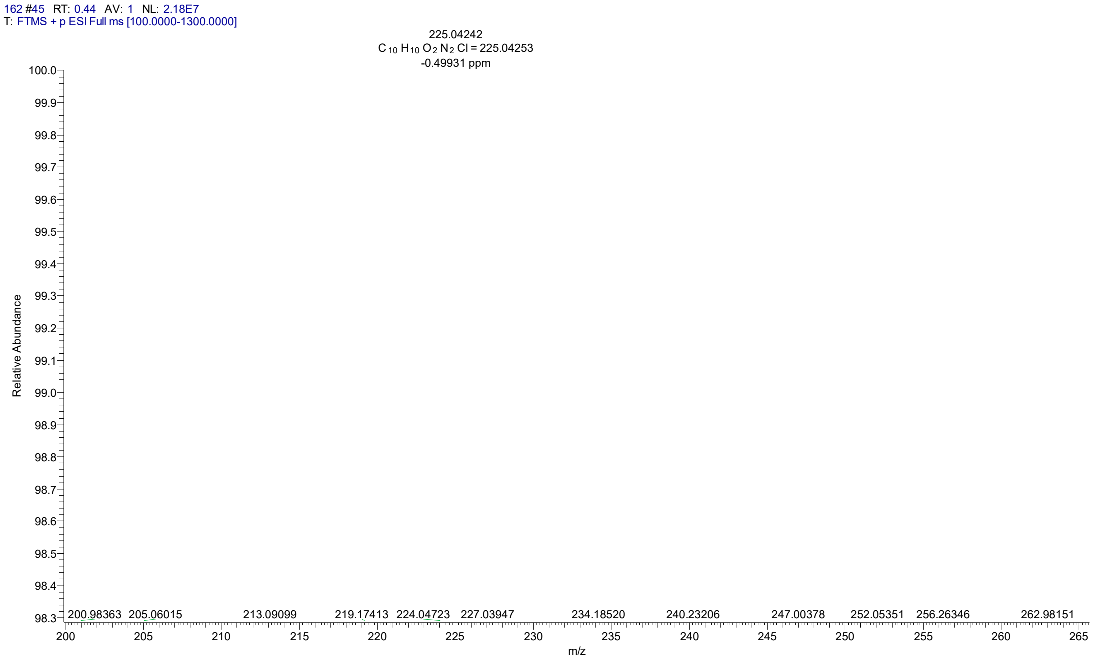

HRMS of compound **B4**

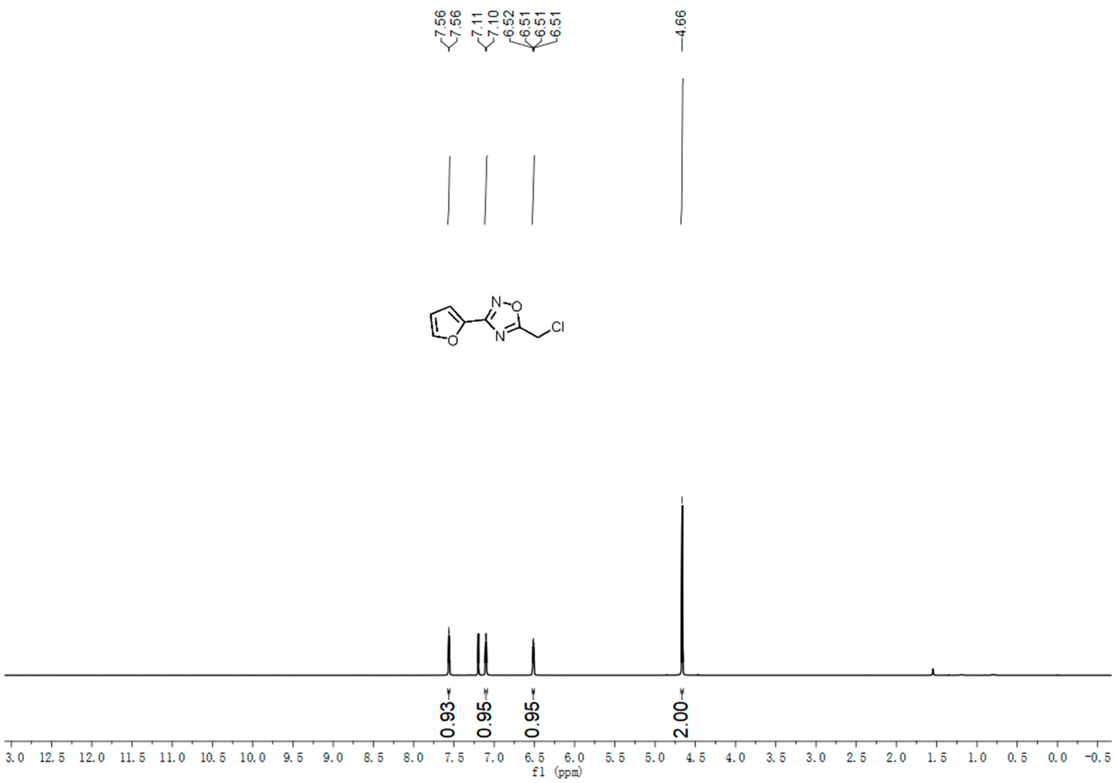

^{1}H NMR of compound **B5**

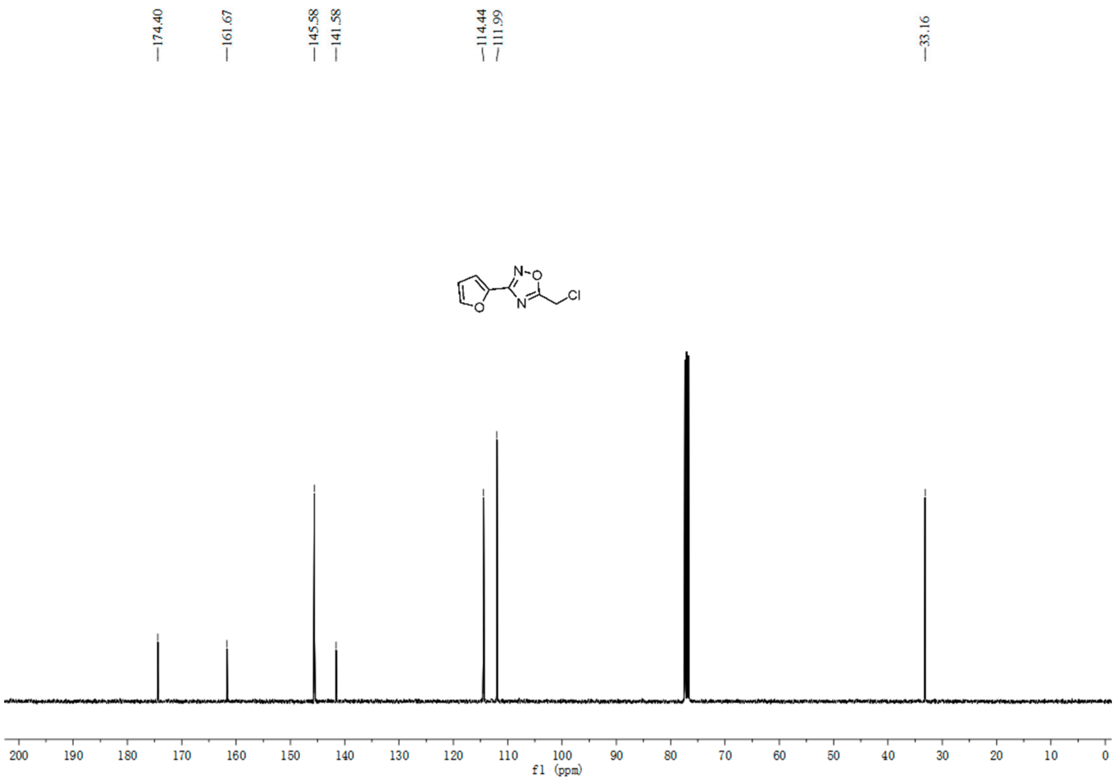

^{13}C NMR of compound **B5**

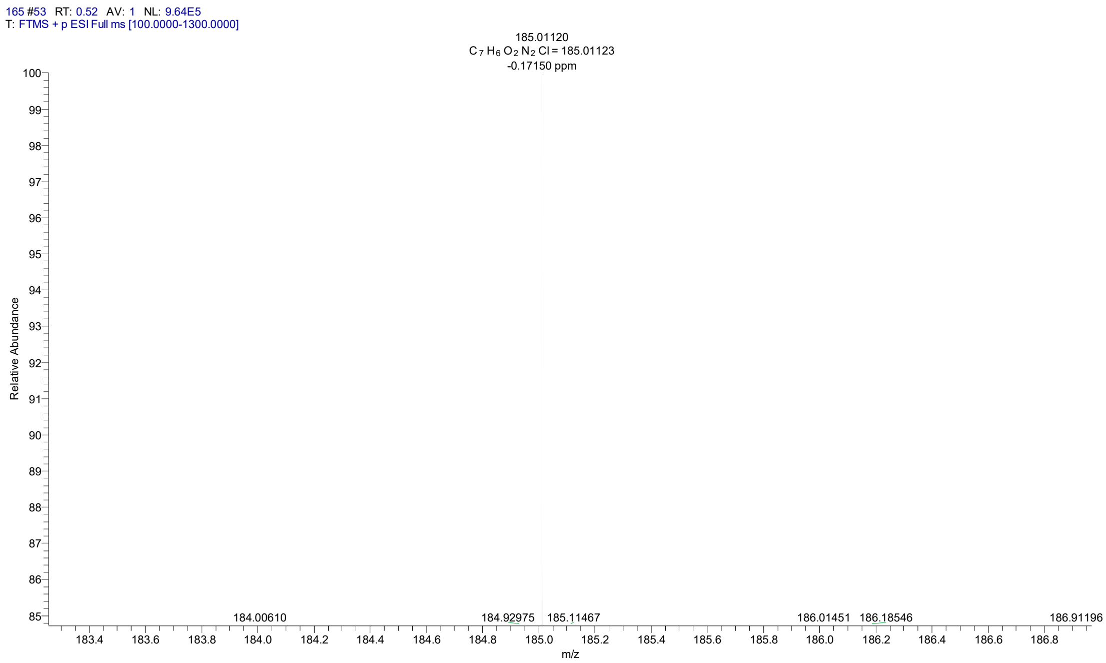

HRMS of compound **B5**

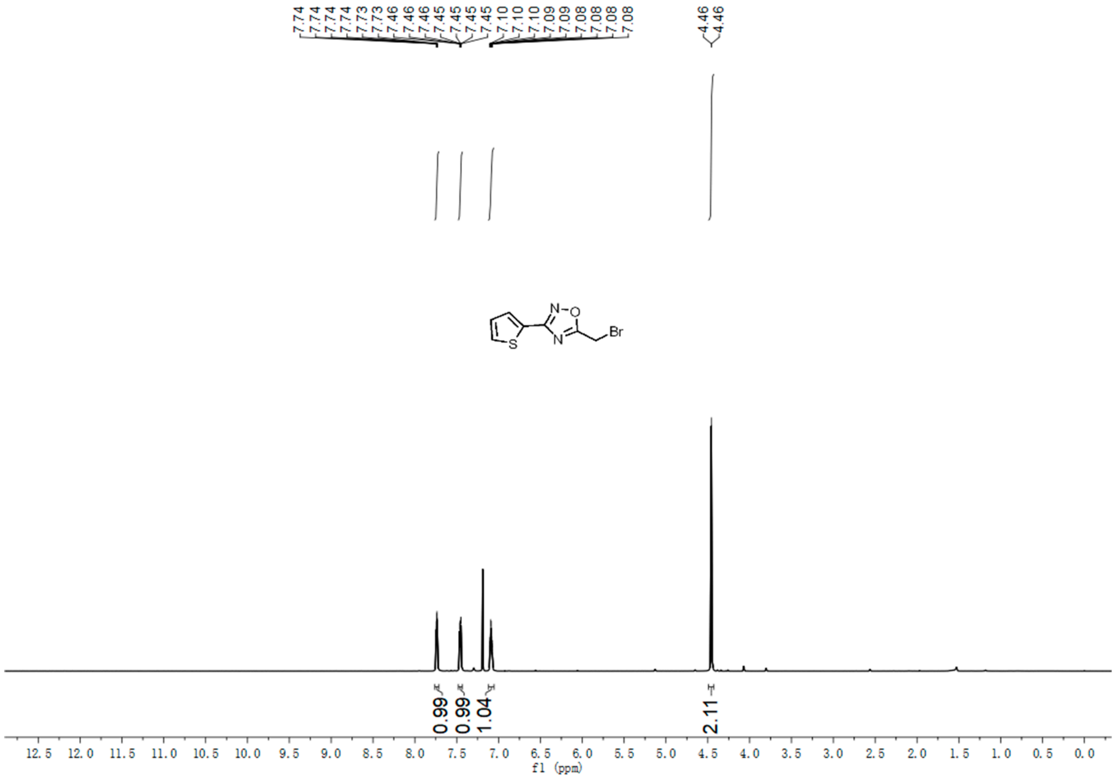

^{1}H NMR of compound **B6**

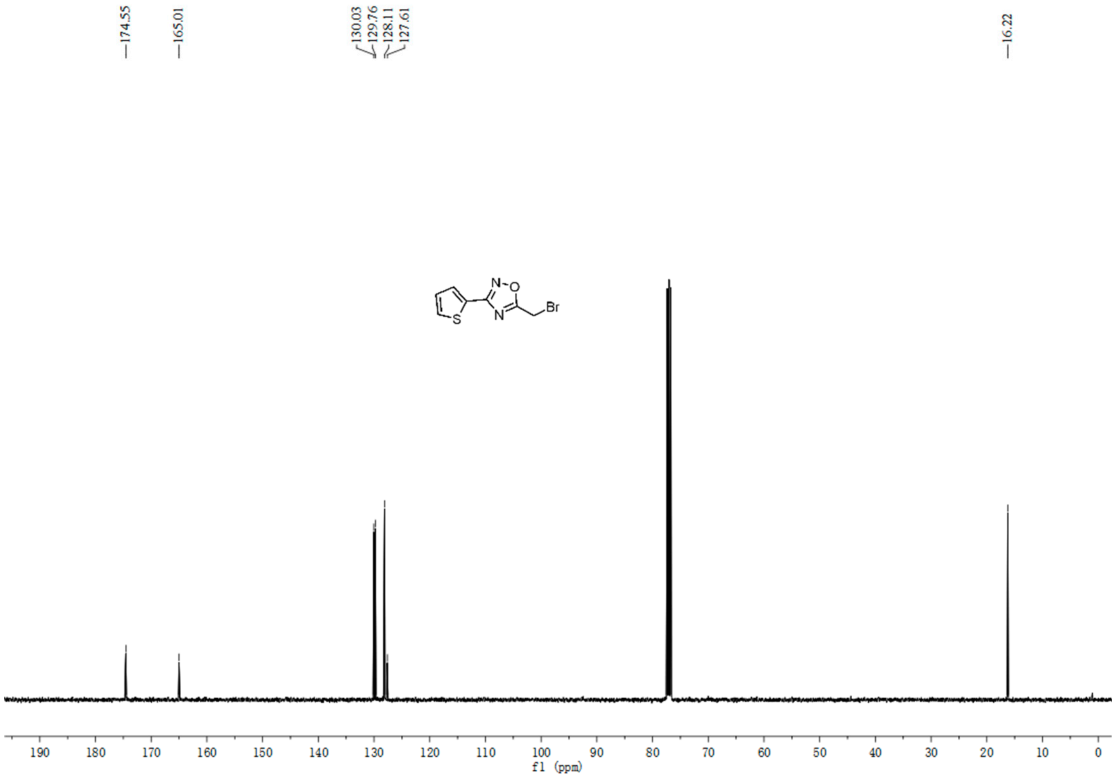

^{13}C NMR of compound **B6**

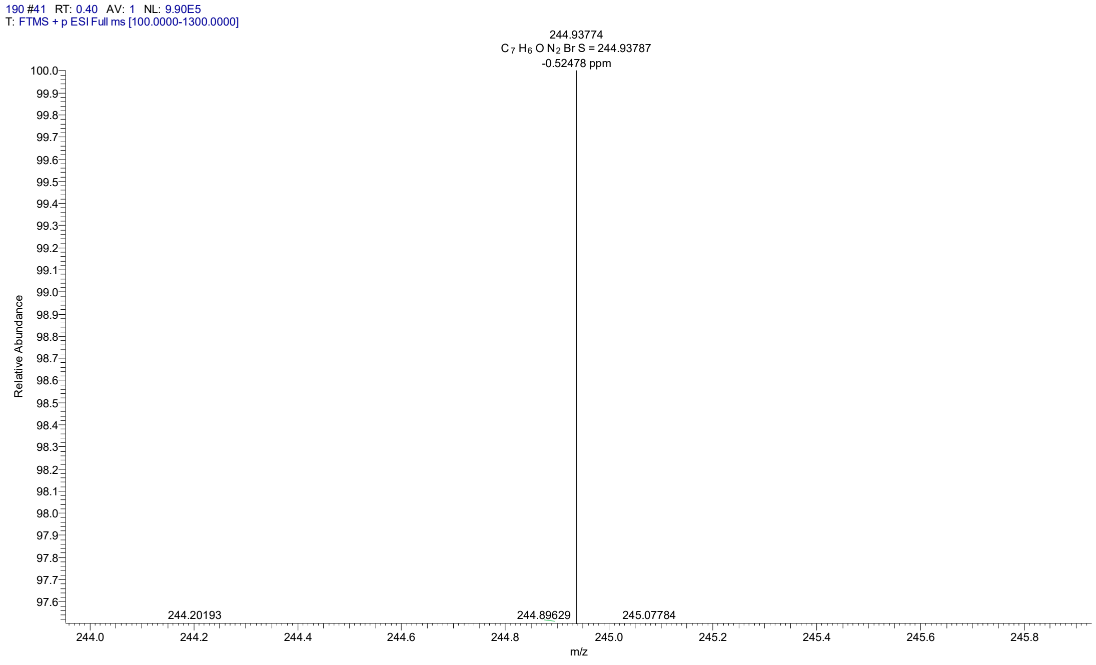

HRMS of compound **B6**

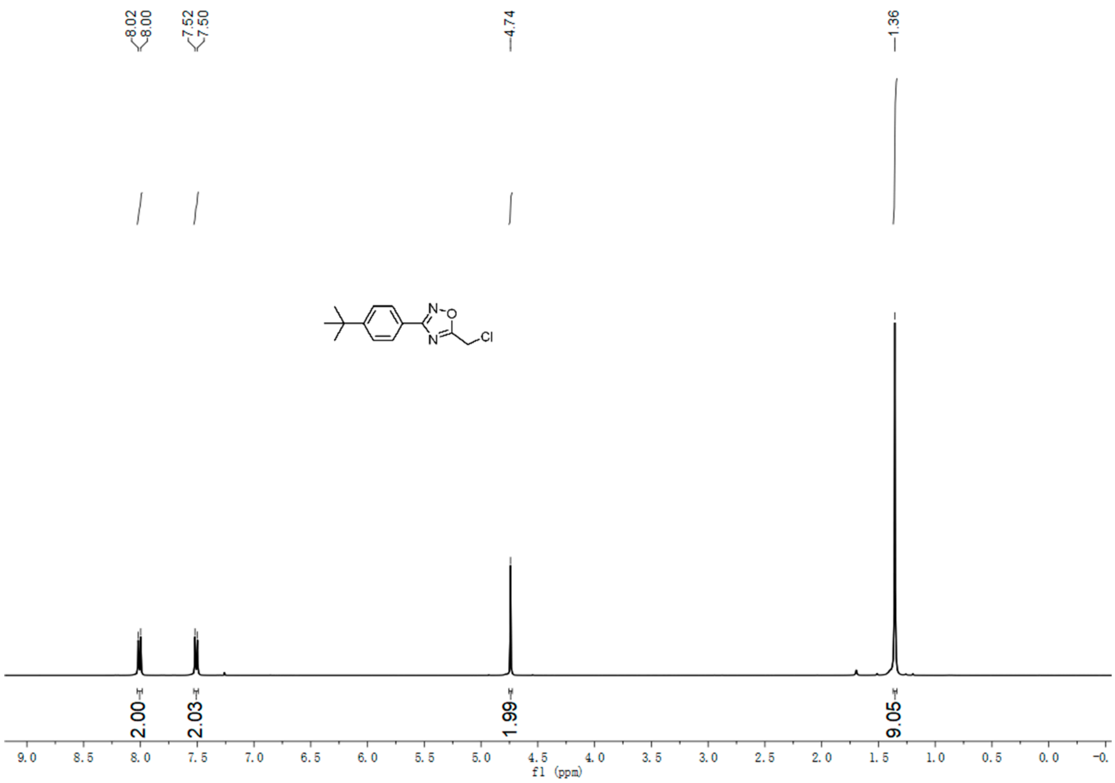

^{1}H NMR of compound **B7**

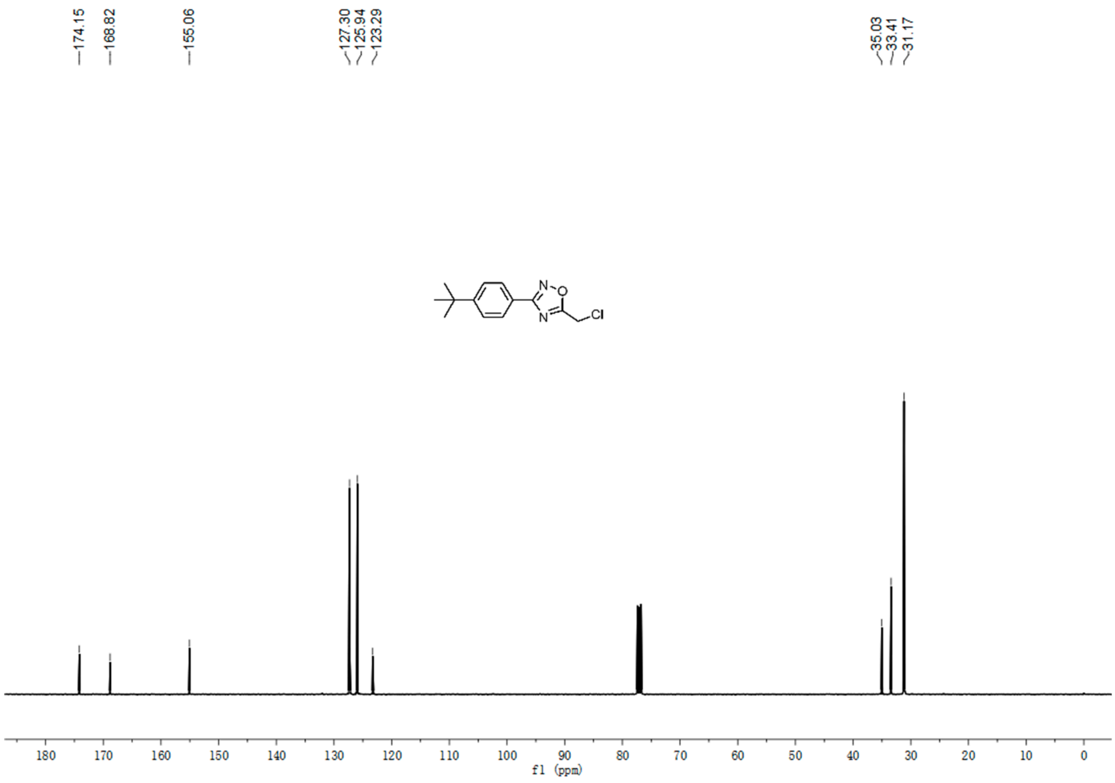

^{13}C NMR of compound **B7**

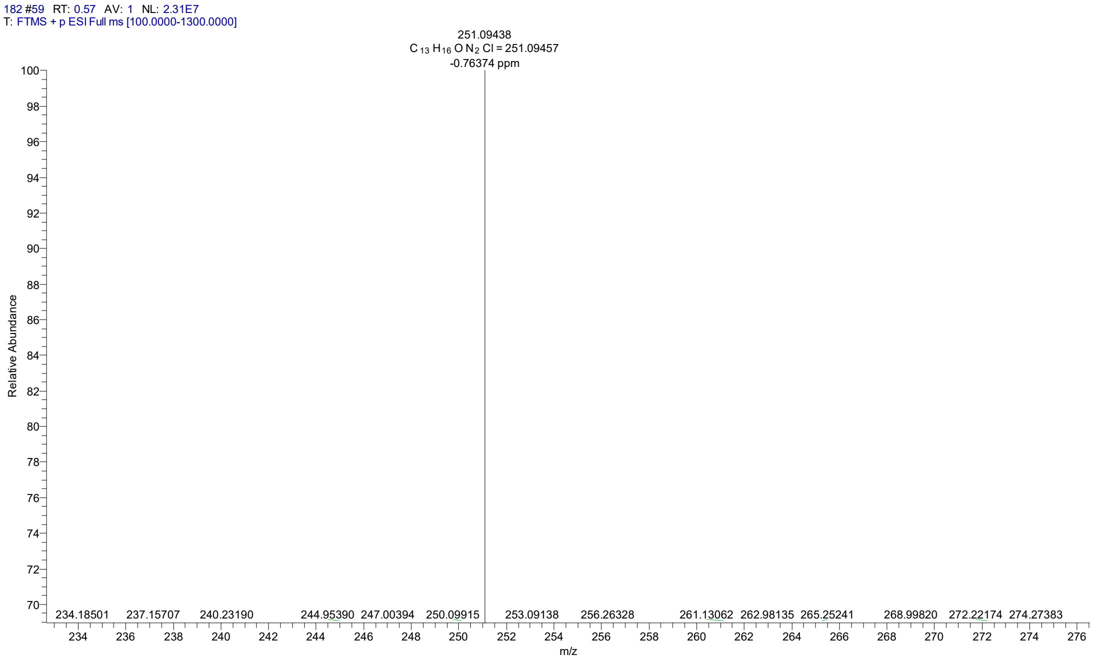

HRMS of compound **B7**

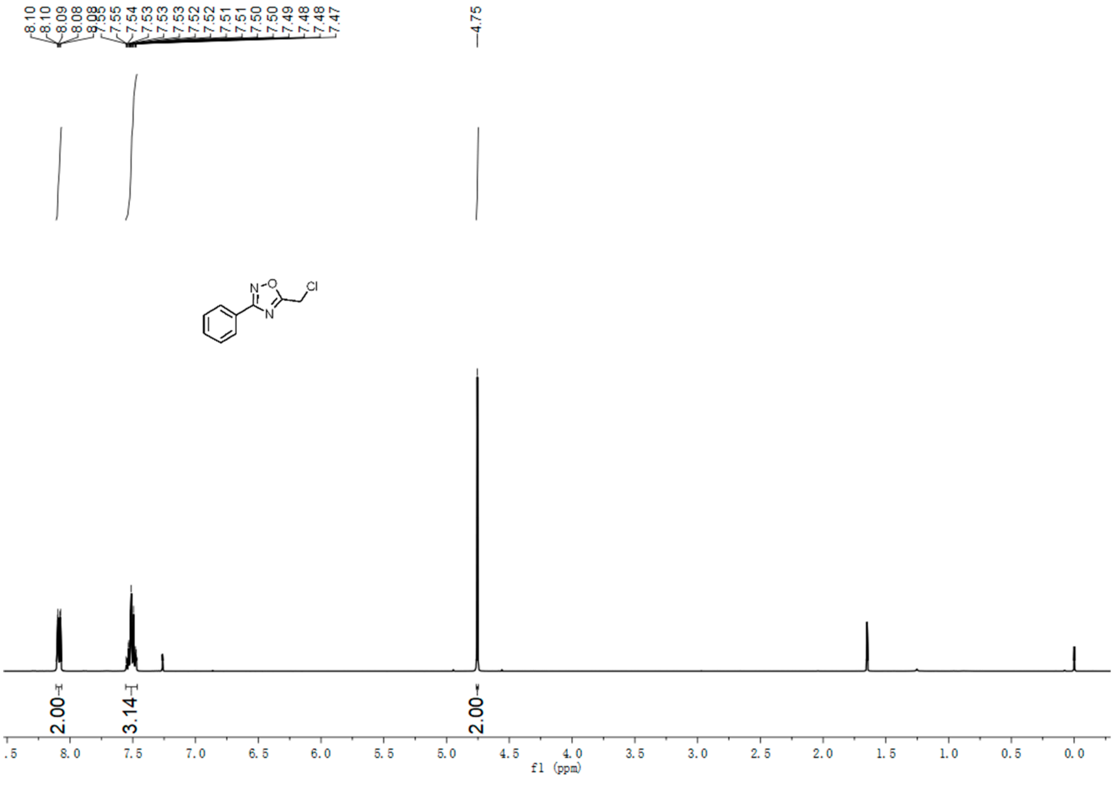

^{1}H NMR of compound **B8**

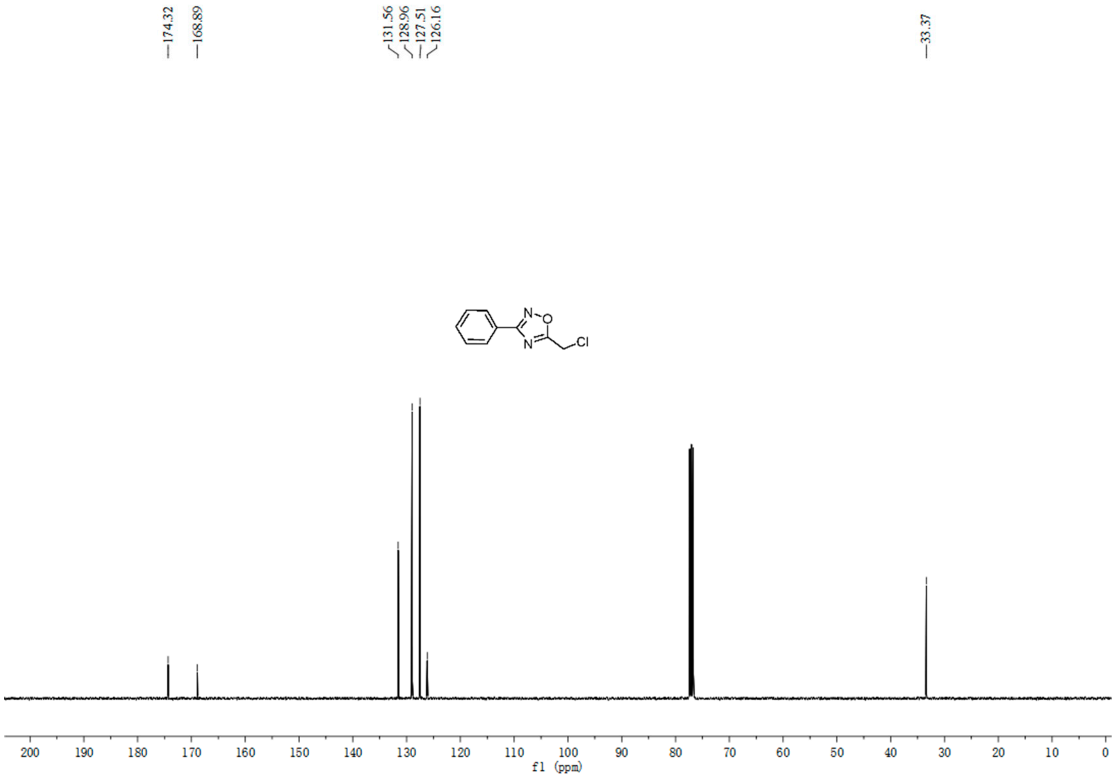

^{13}C NMR of compound **B8**

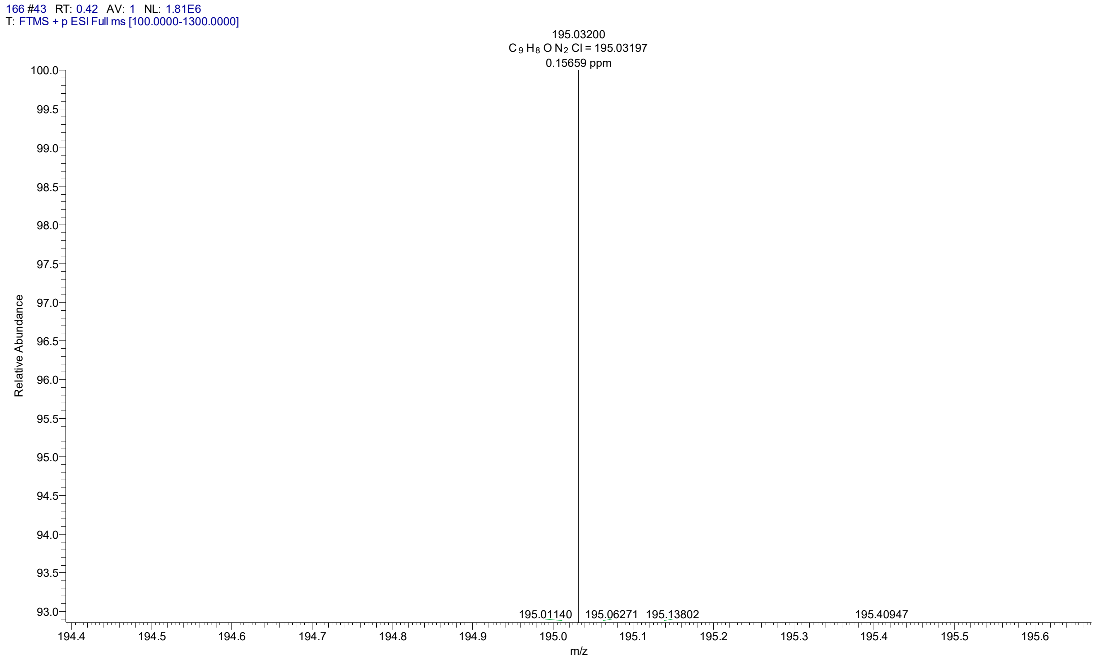

HRMS of compound **B8**

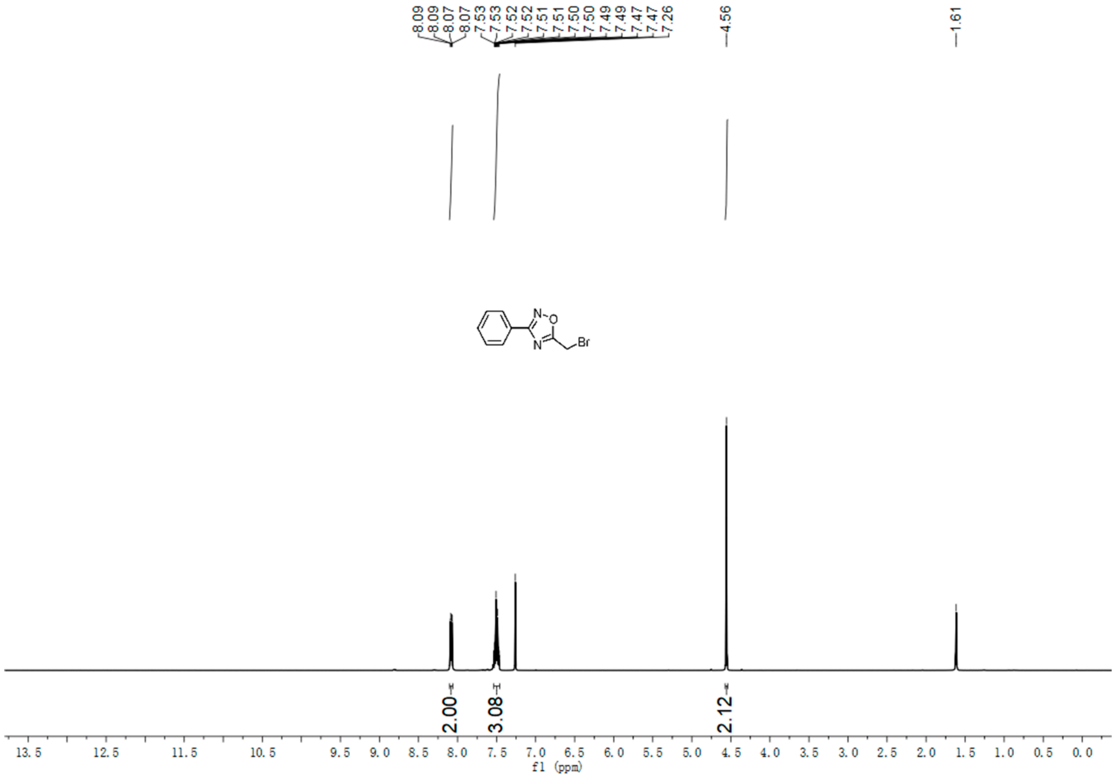

^{1}H NMR of compound **B9**

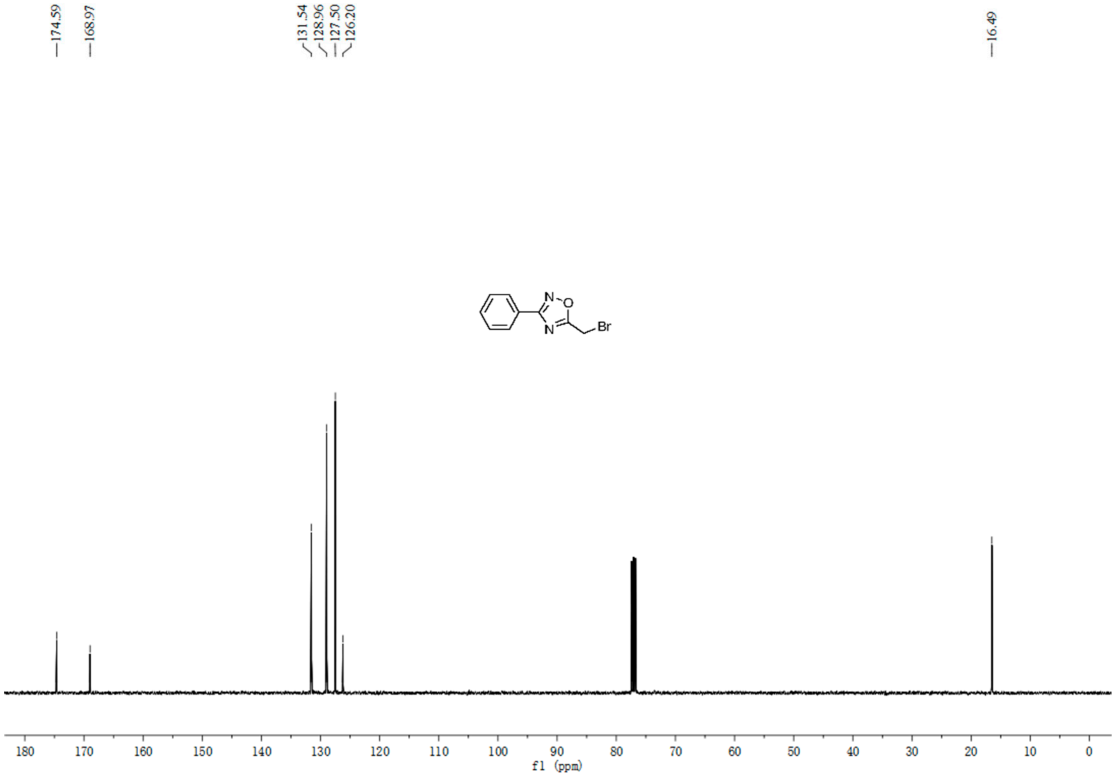

^{13}C NMR of compound **B9**

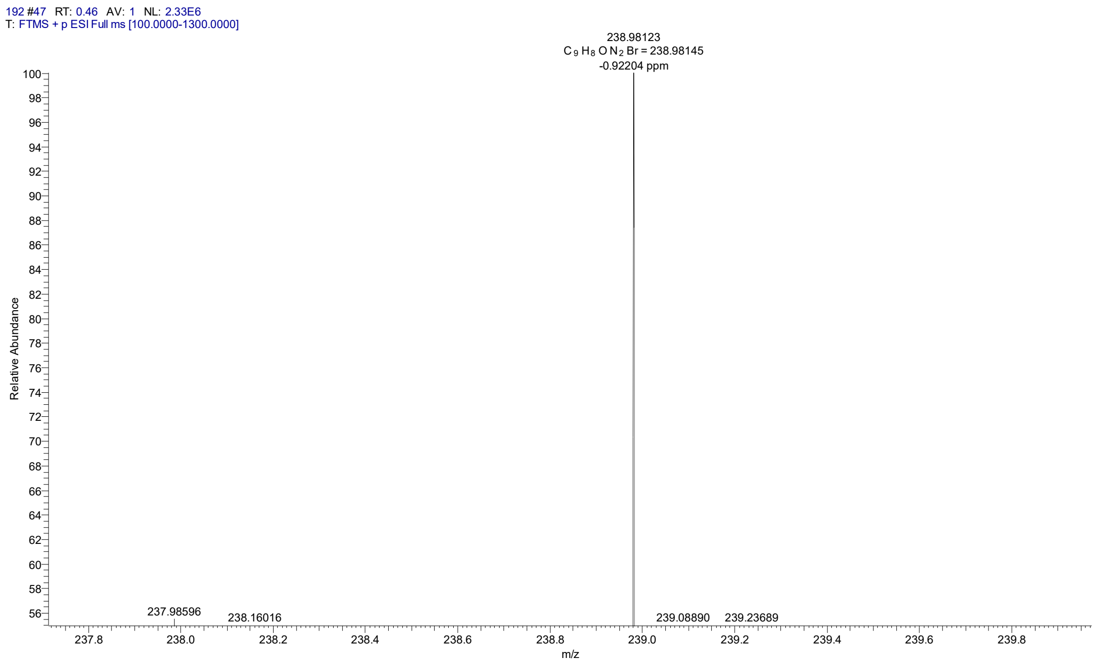

HRMS of compound **B9**

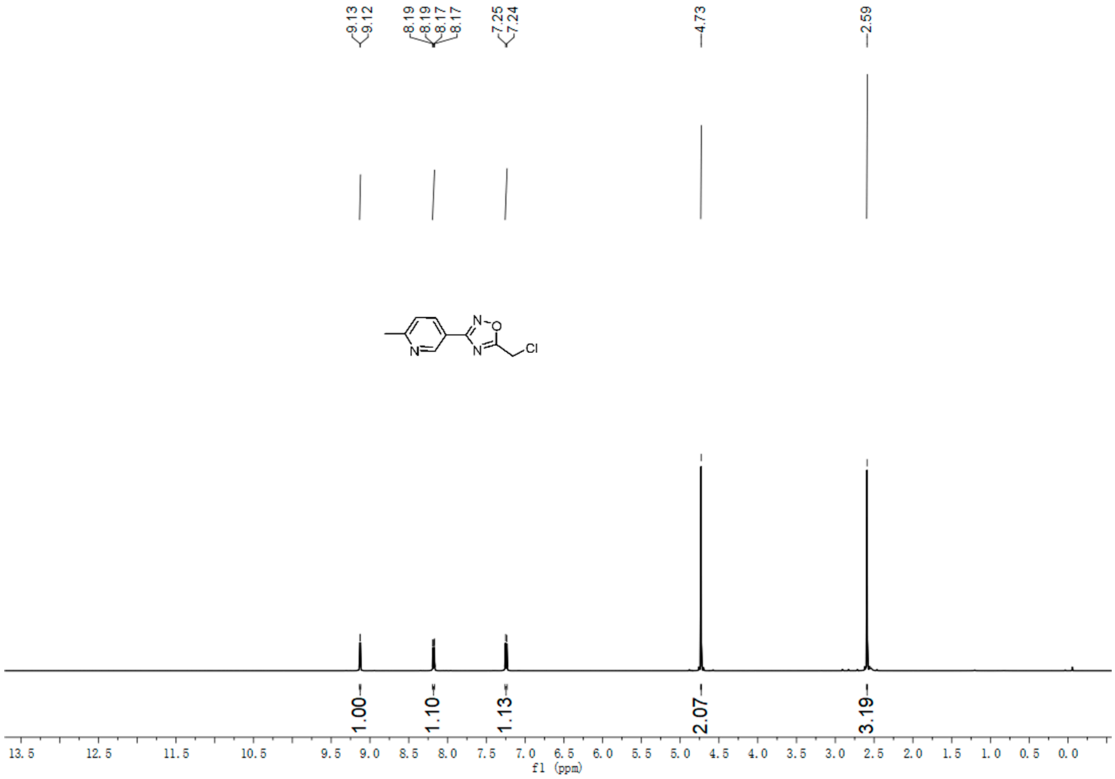

^{1}H NMR of compound **B10**

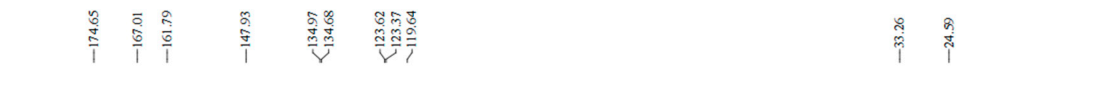

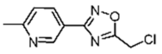

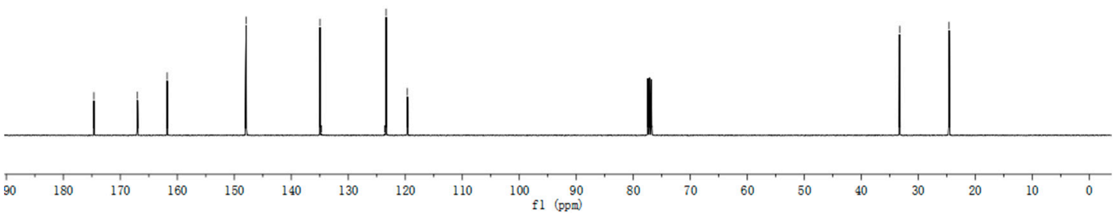

^{13}C NMR of compound **B10**

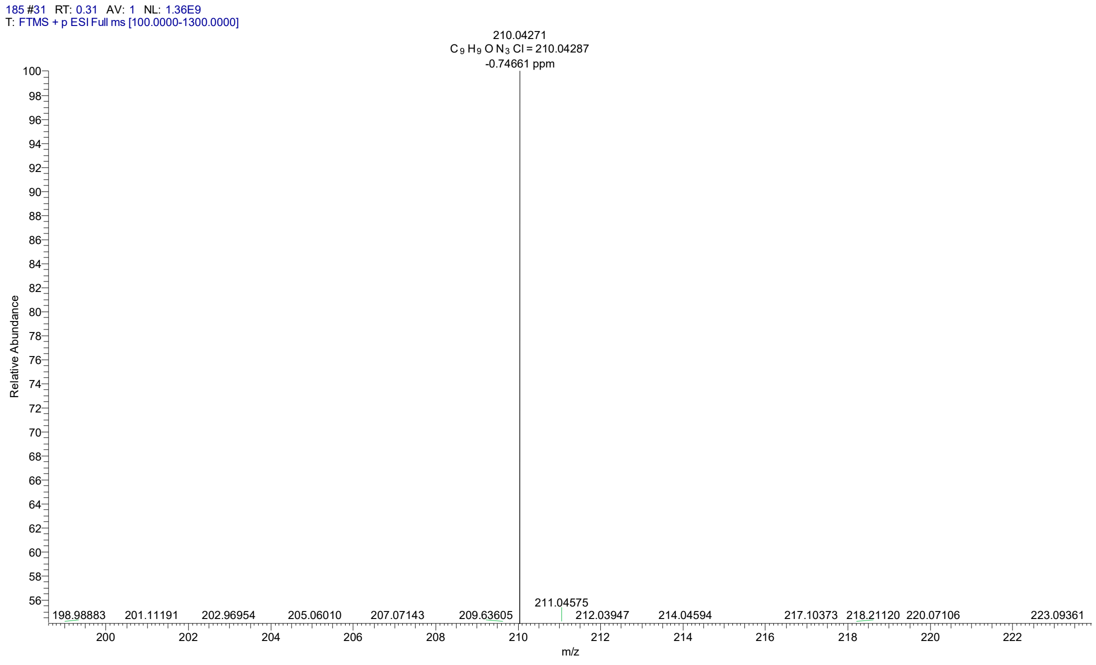

HRMS of compound **B10**

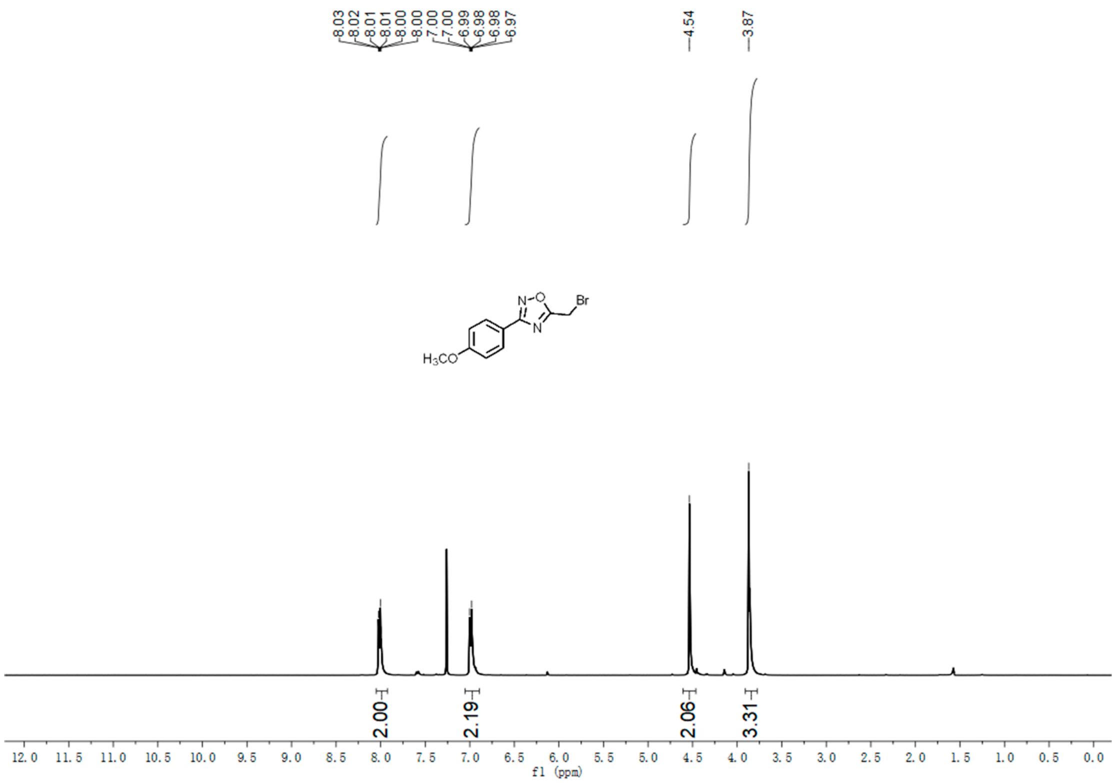

^{1}H NMR of compound **B11**

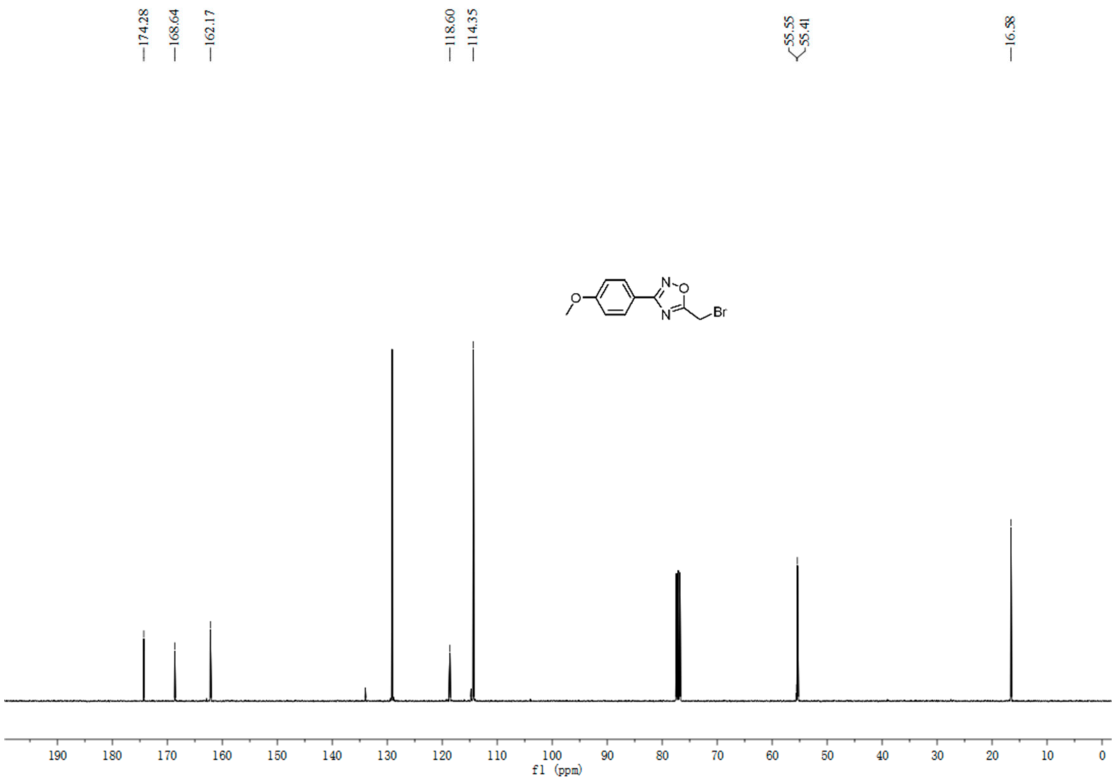

^{13}C NMR of compound **B11**

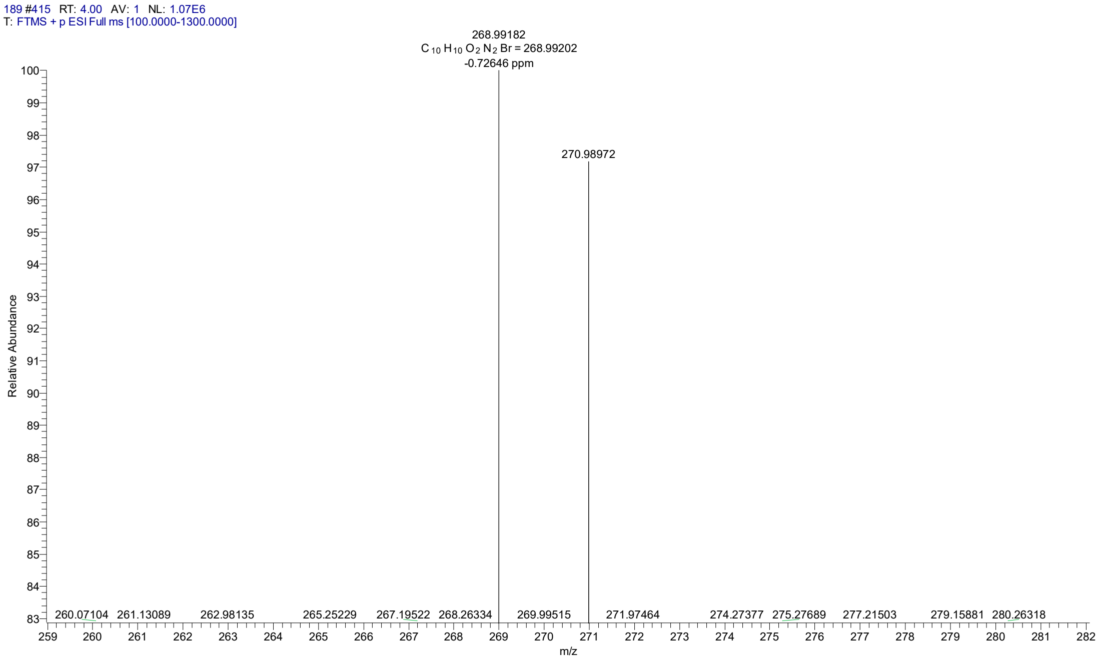

HRMS of compound **B11**

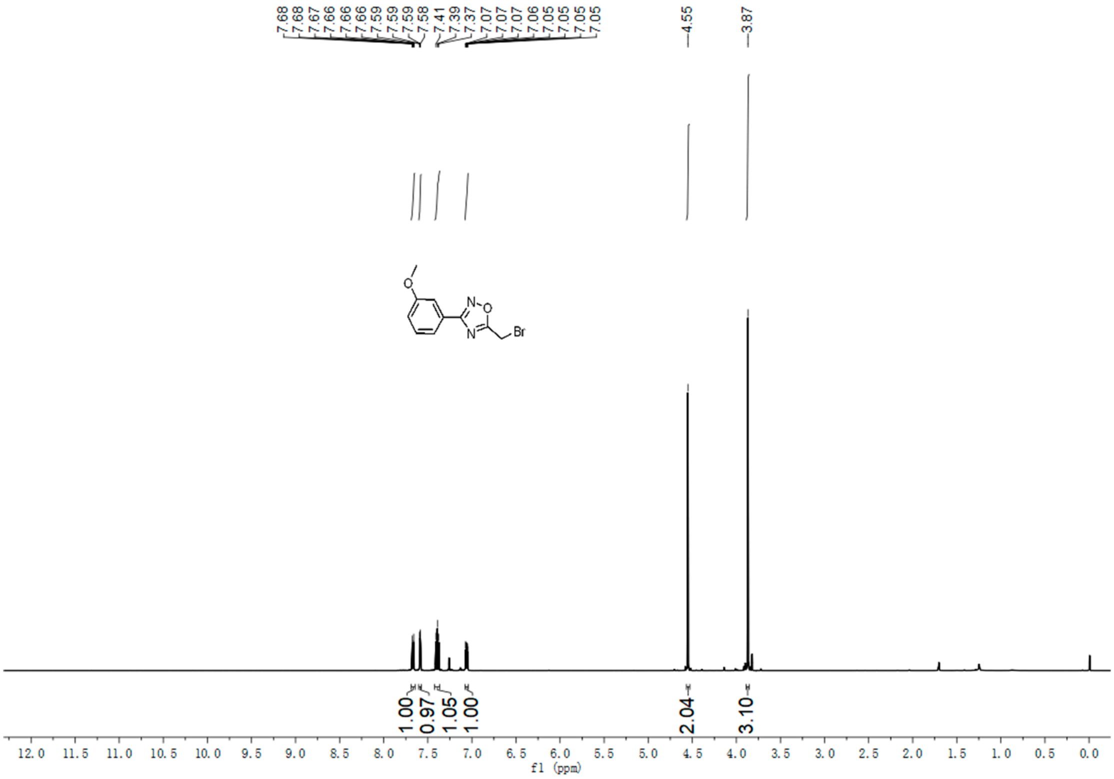

^{1}H NMR of compound **B12**

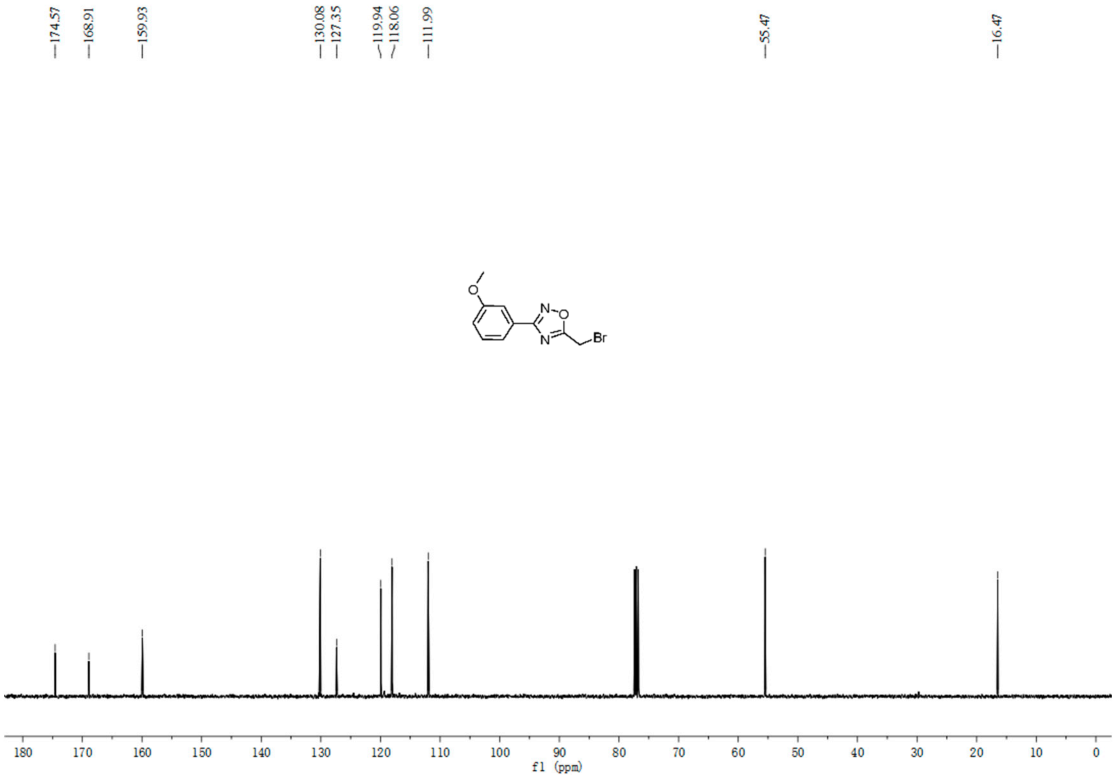

^{13}C NMR of compound **B12**

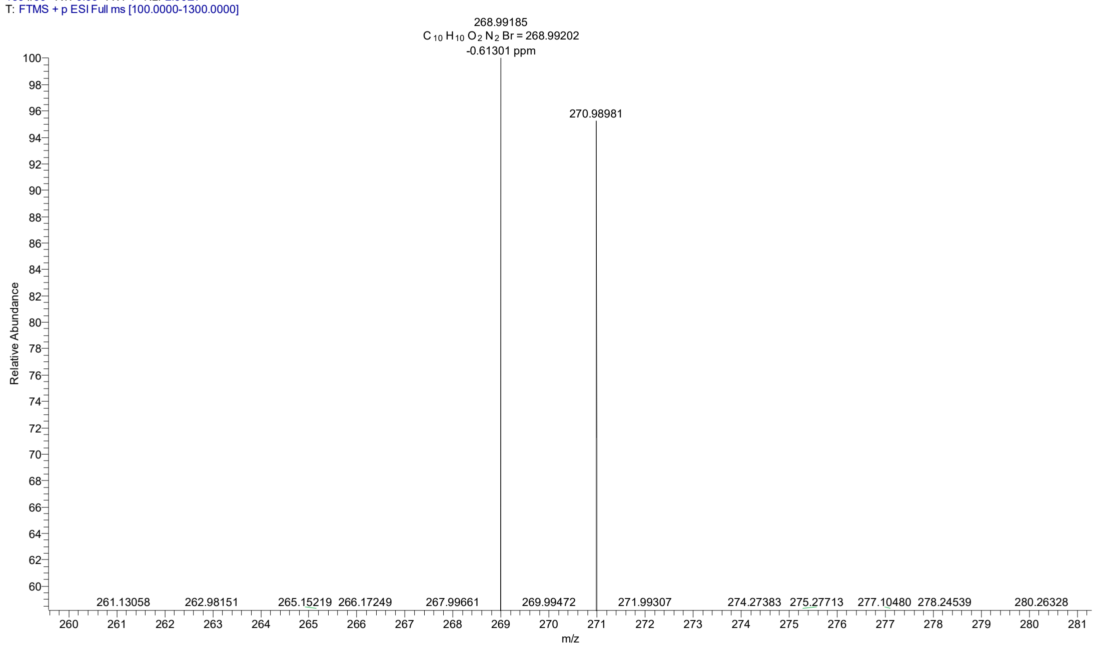

HRMS of compound **B12**

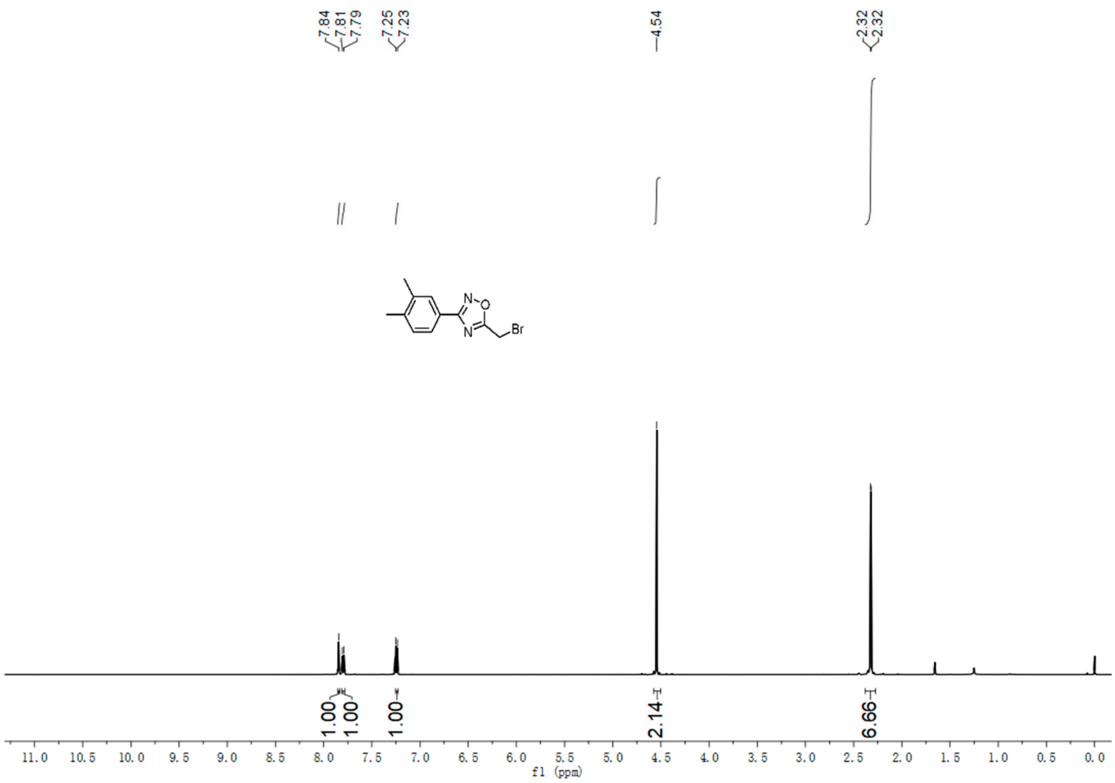

^{1}H NMR of compound **B13**

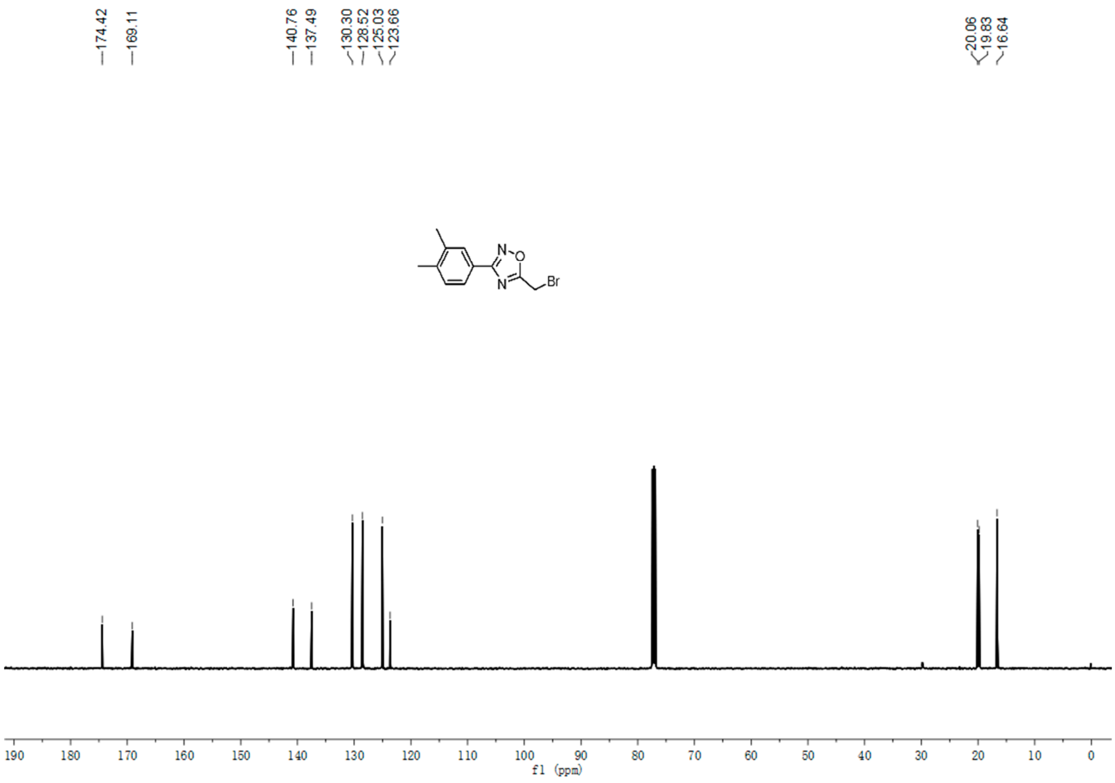

^{13}C NMR of compound **B13**

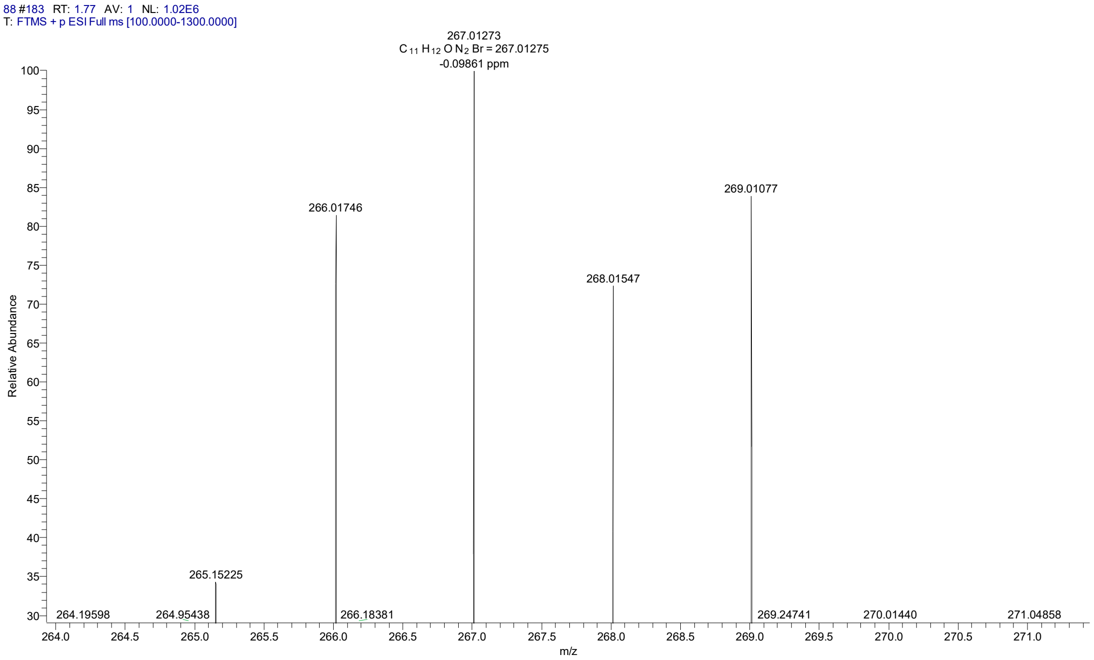

HRMS of compound **B13**

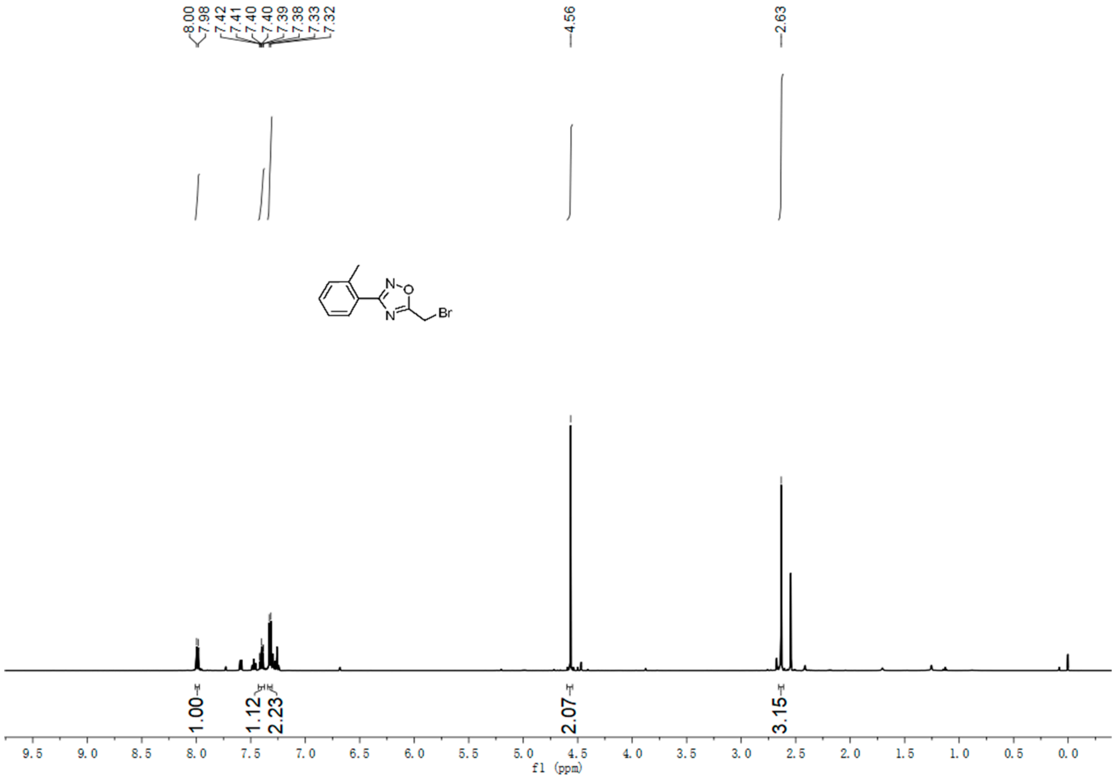

^{1}H NMR of compound **B14**

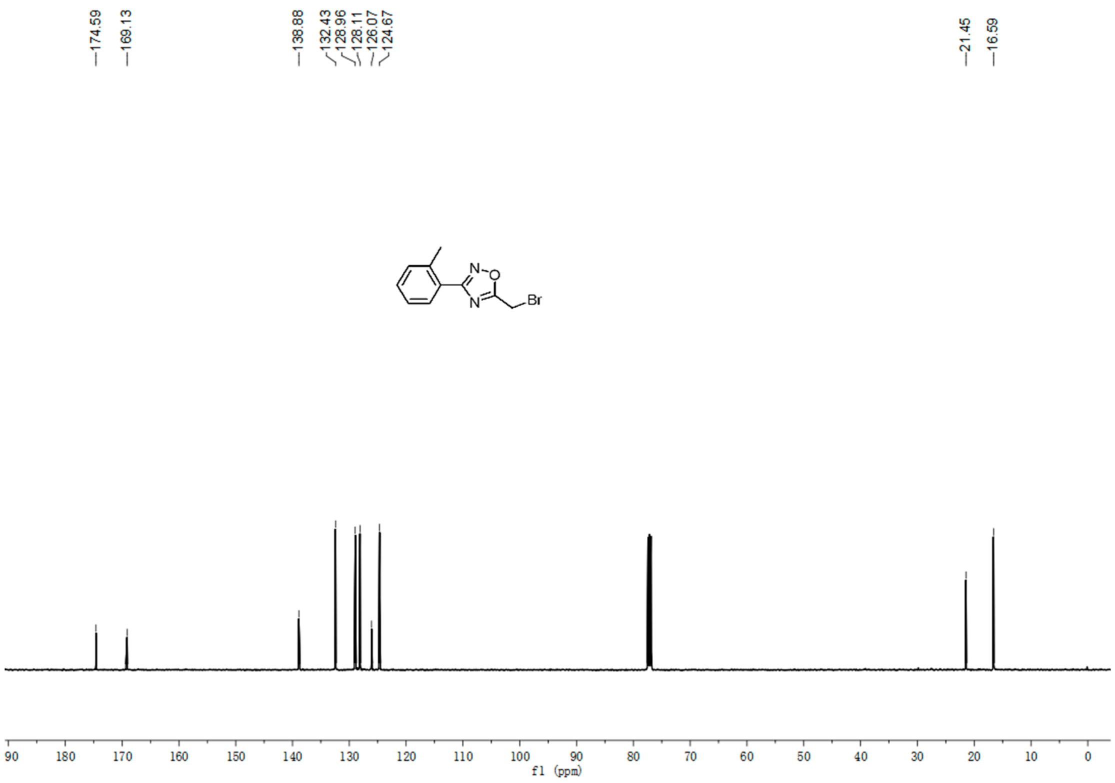

^{13}C NMR of compound **B14**

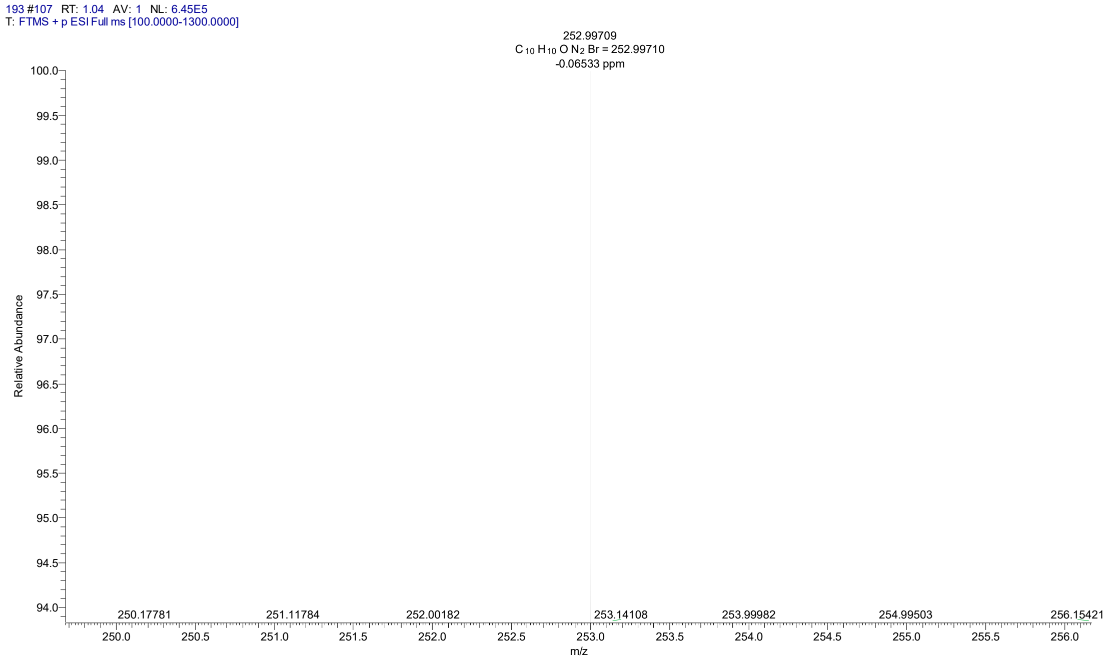

HRMS of compound **B14**

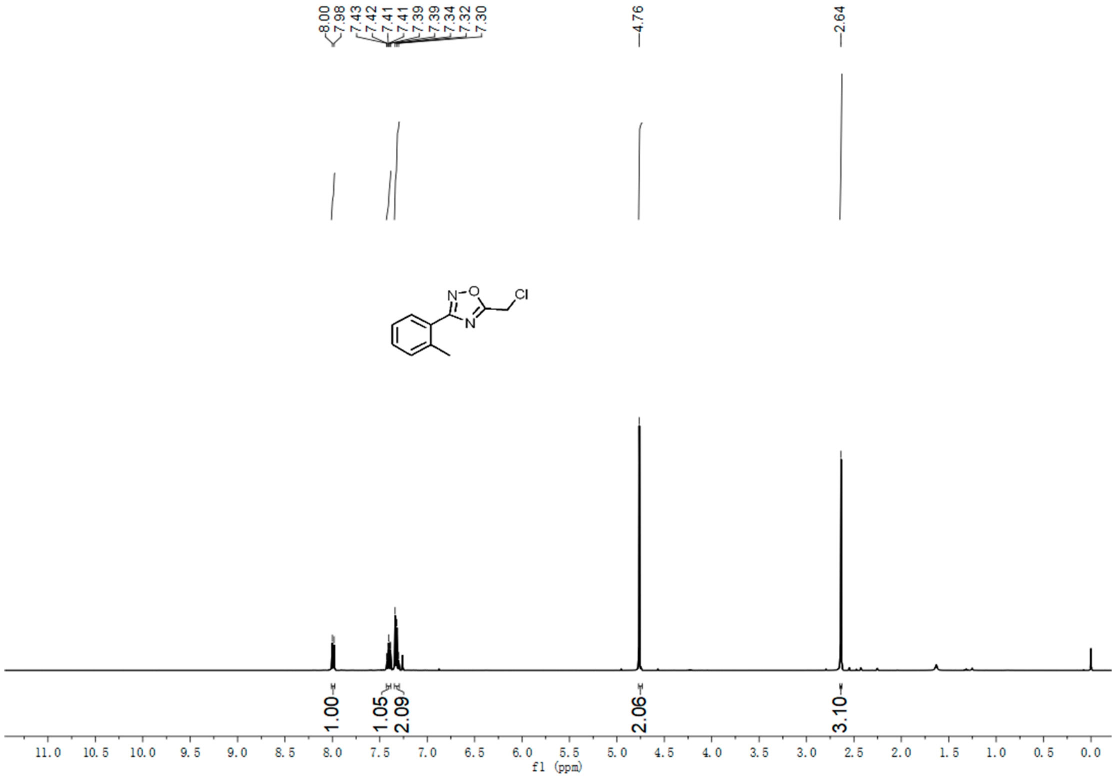

^{1}H NMR of compound **B15**

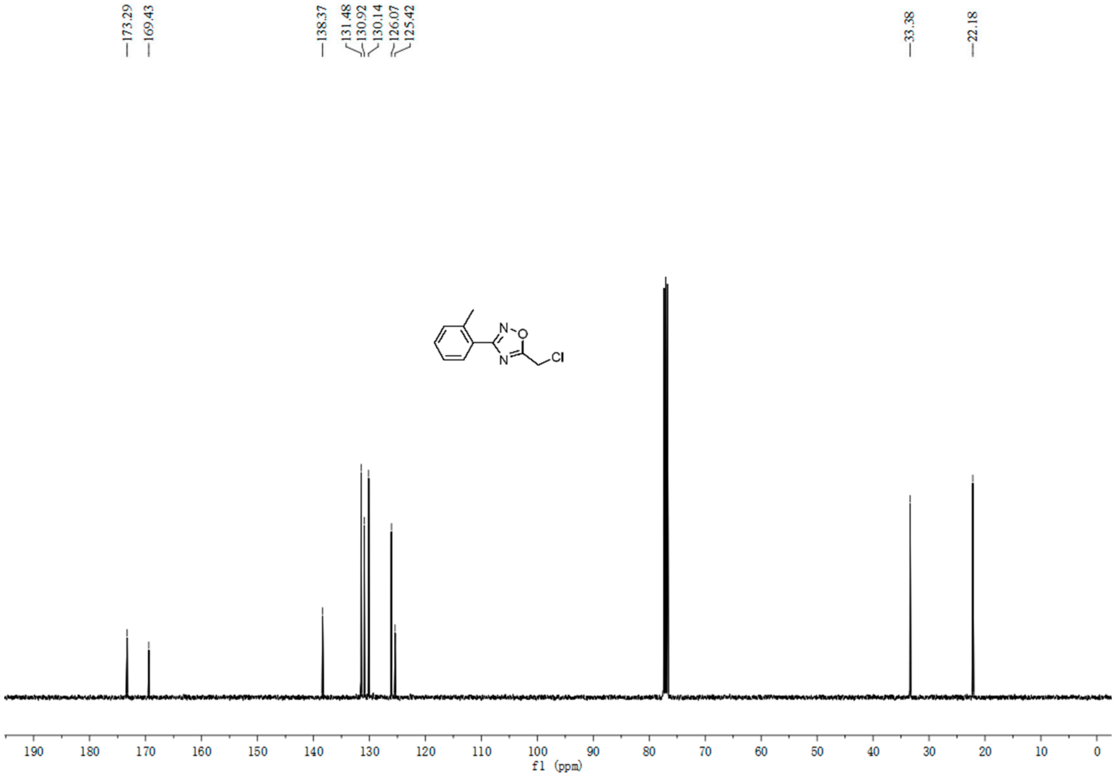

^{13}C NMR of compound **B15**

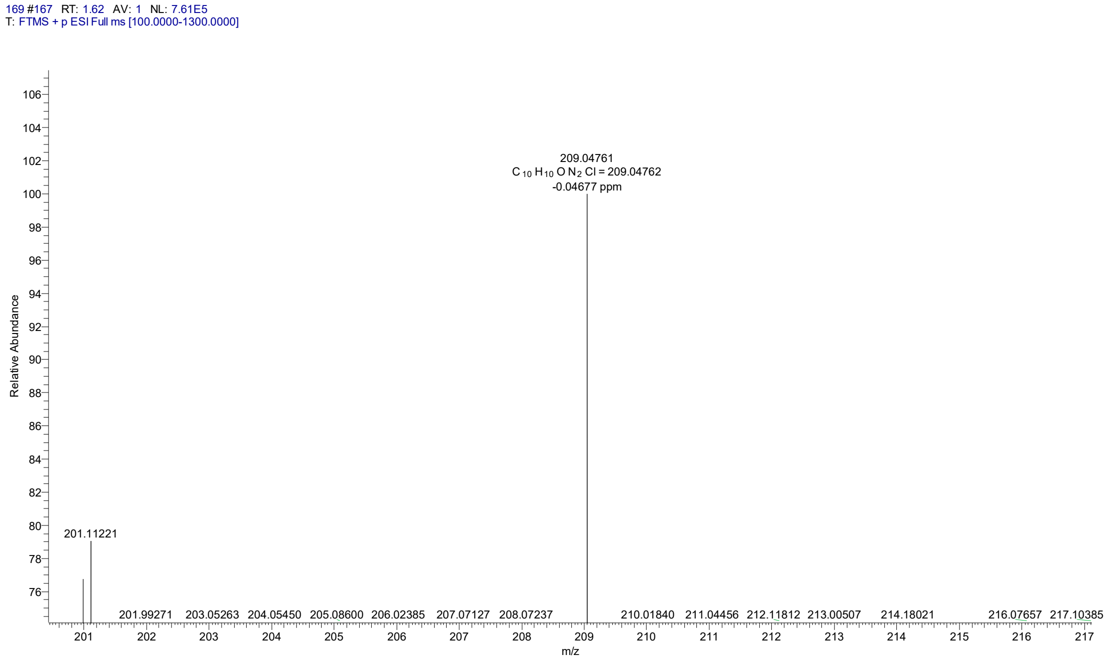

HRMS of compound **B15**

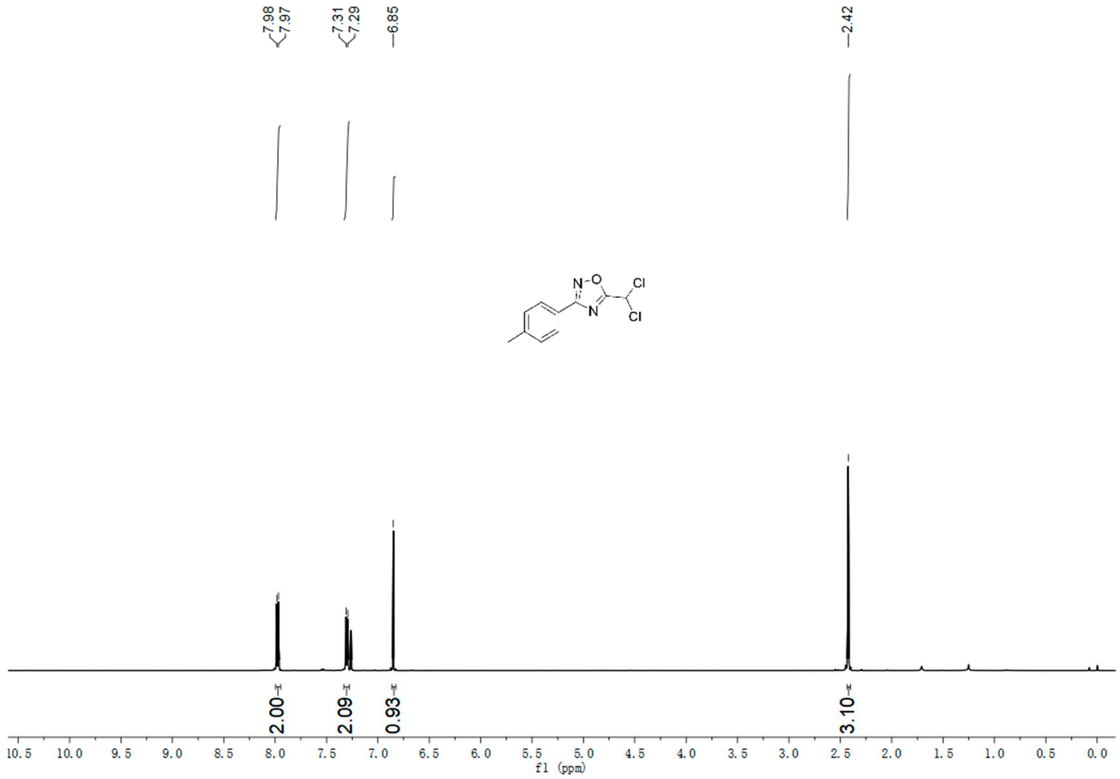

^{1}H NMR of compound **B16**

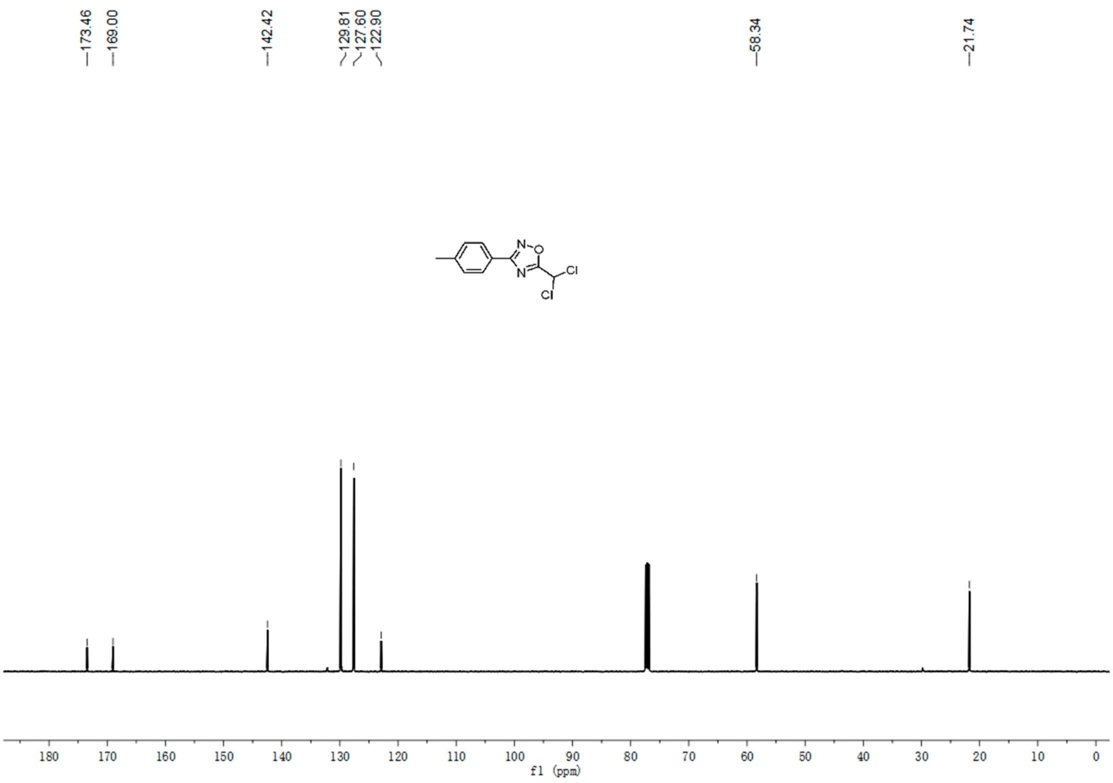

^{13}C NMR of compound **B16**

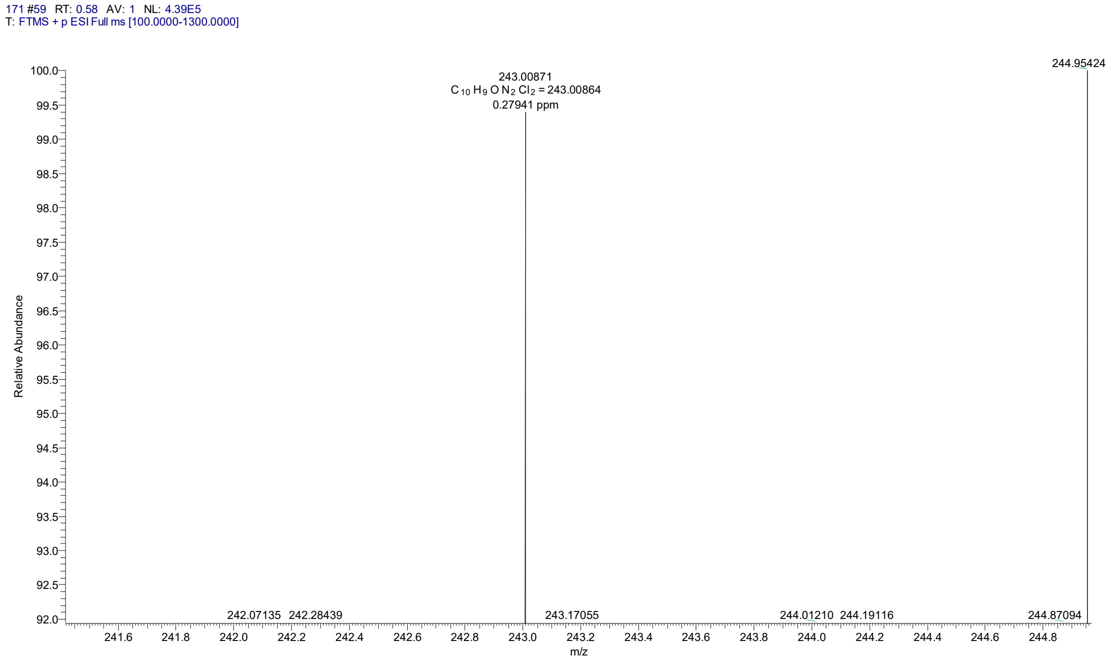

HRMS of compound **B16**

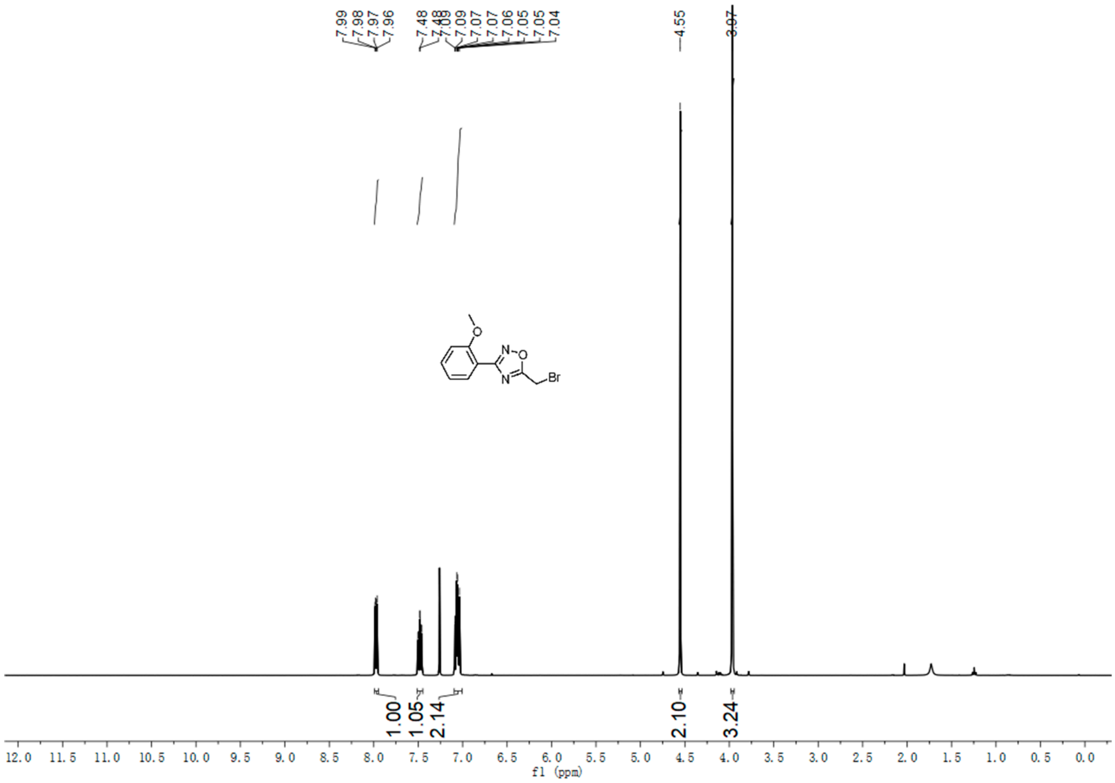

^{1}H NMR of compound **B17**

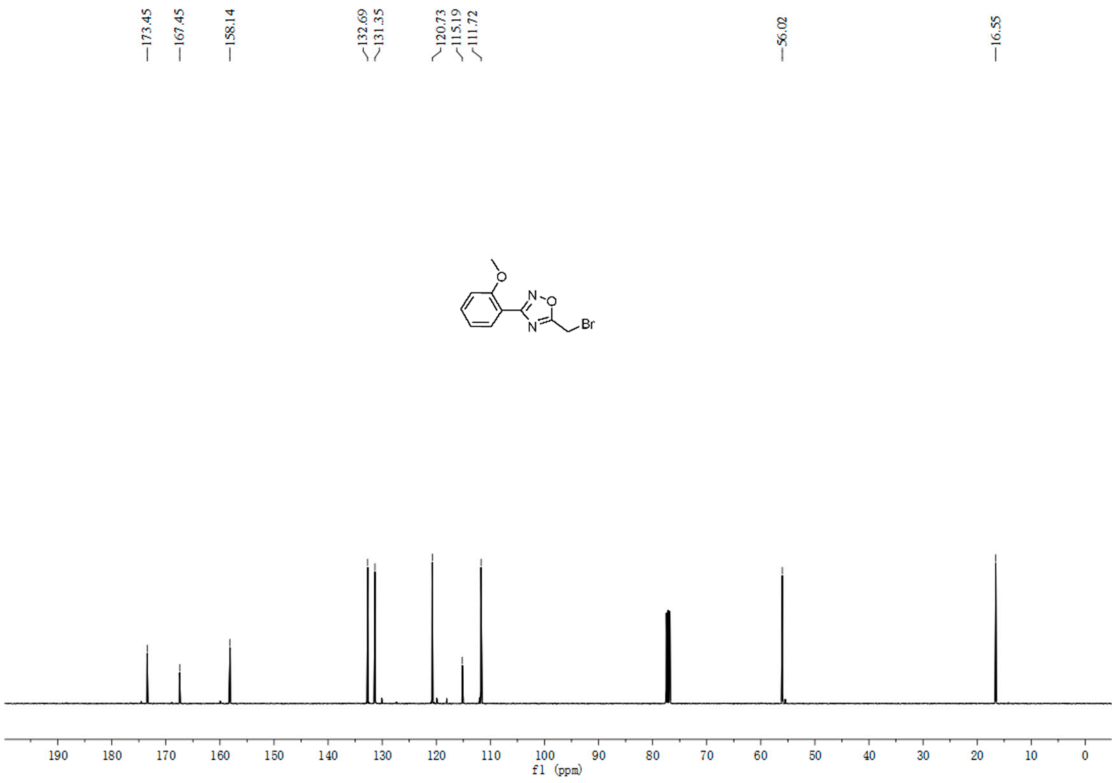

^{13}C NMR of compound **B17**

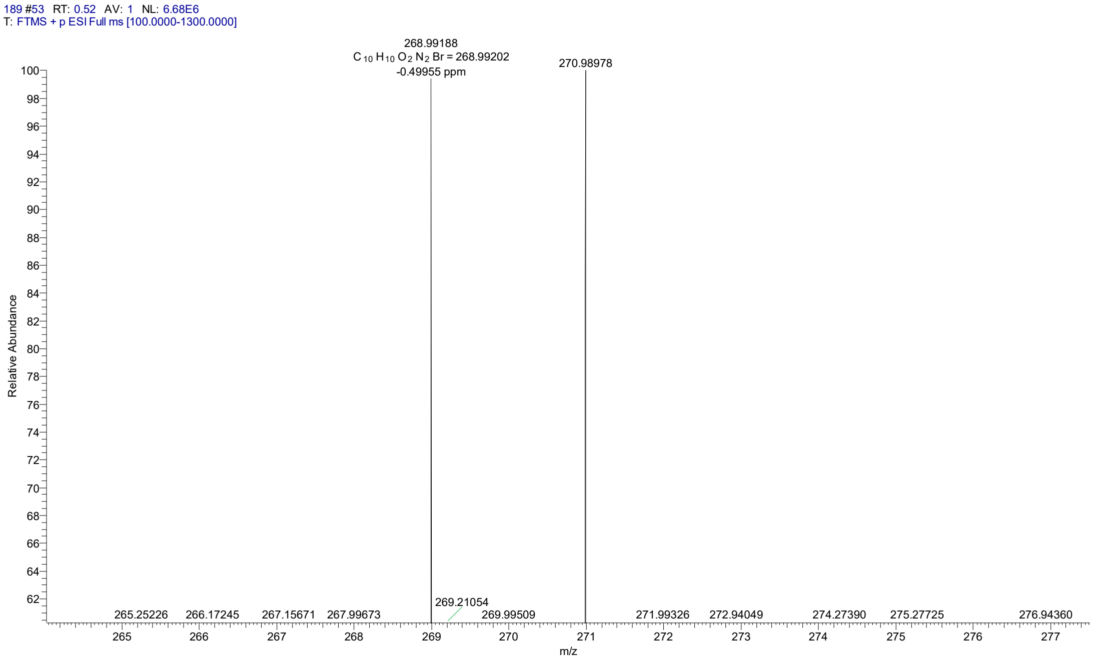

HRMS of compound **B17**

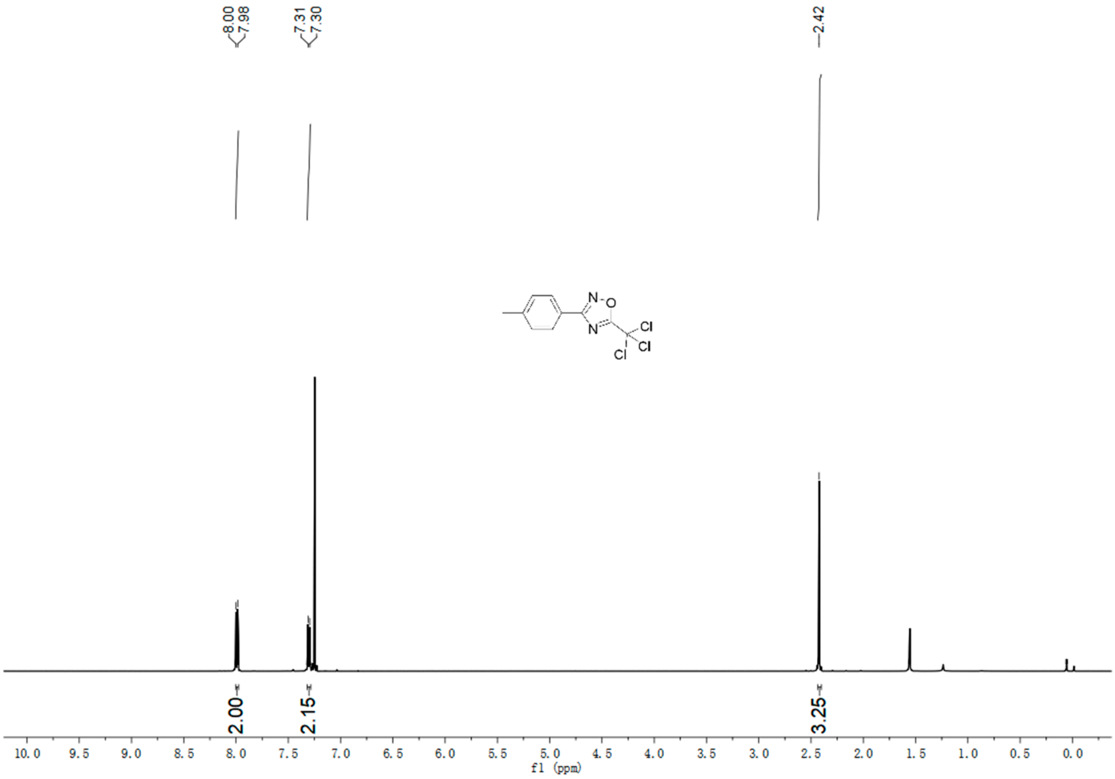

^{1}H NMR of compound **B18**

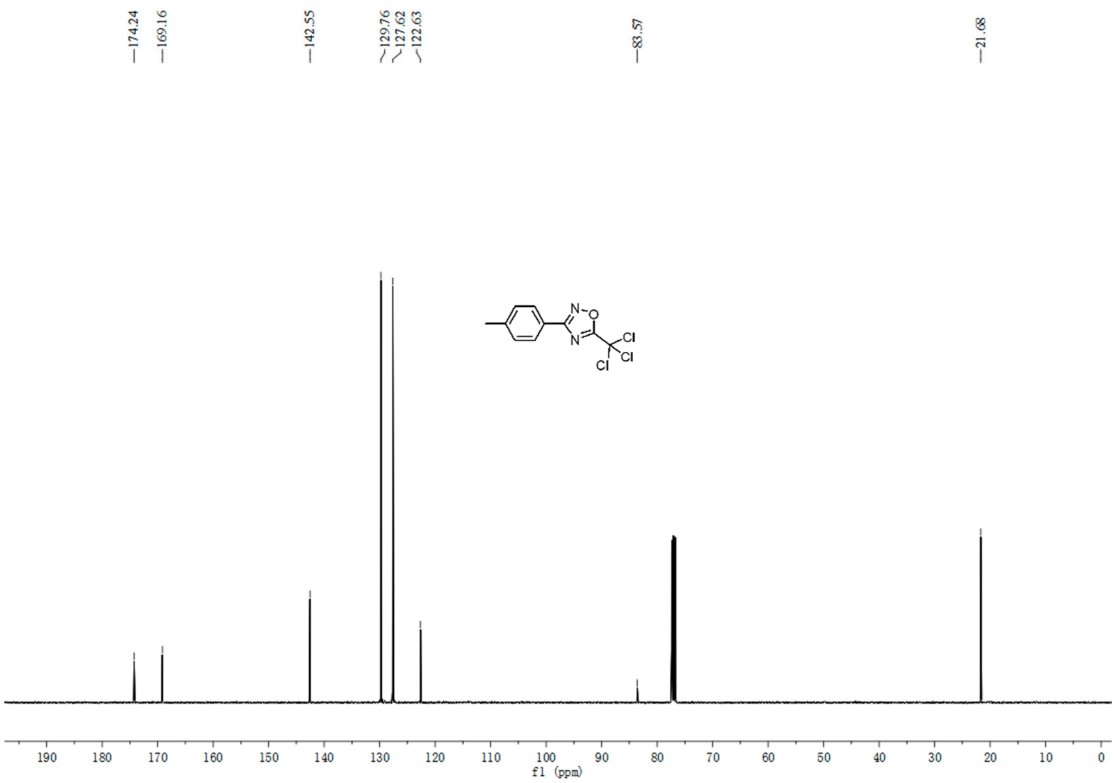

^{13}C NMR of compound **B18**

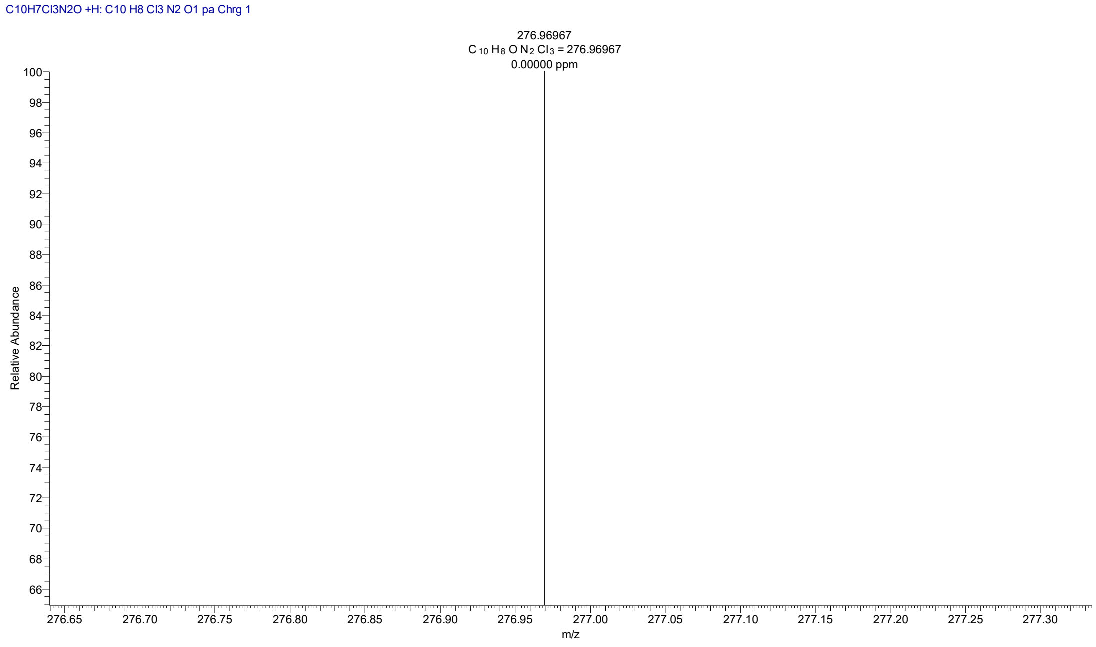

HRMS of compound **B18**

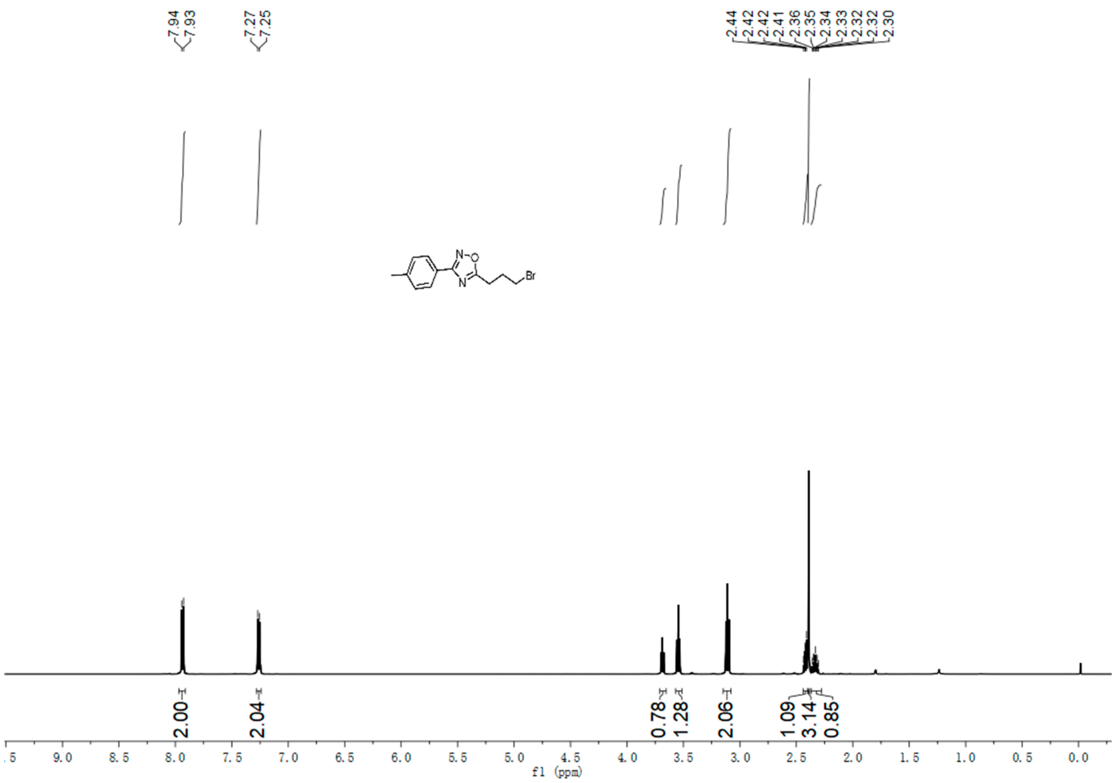

^{1}H NMR of compound **B19**

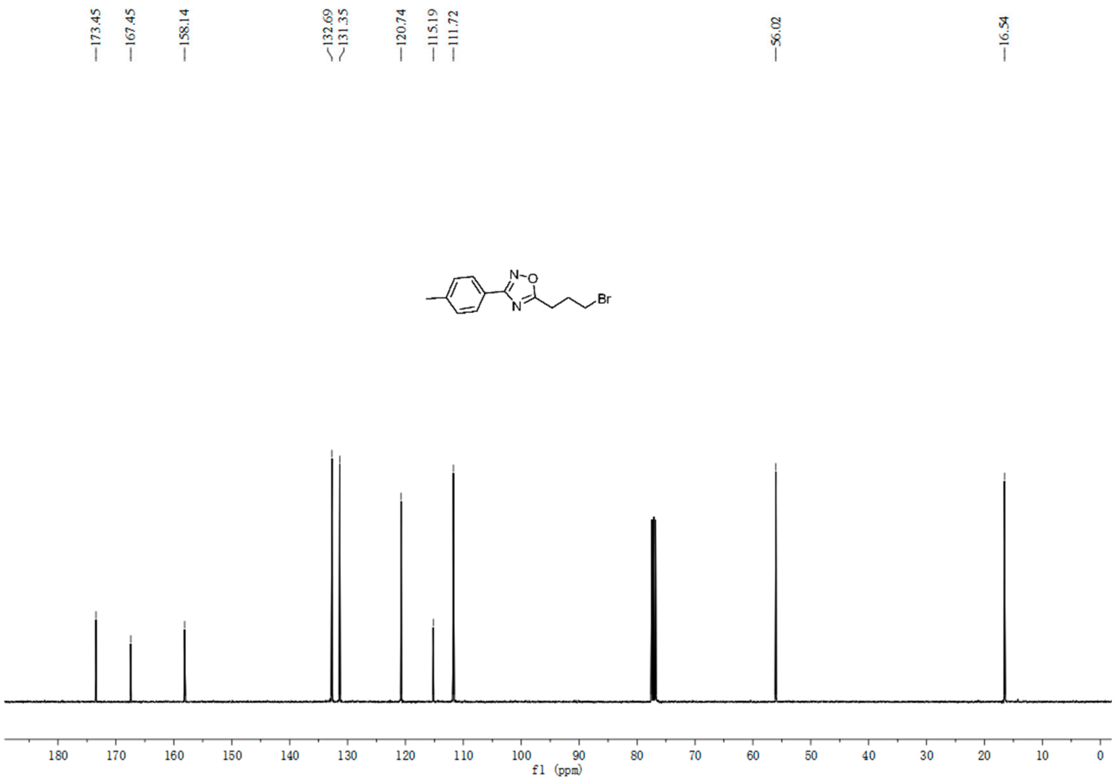

^{13}C NMR of compound **B19**

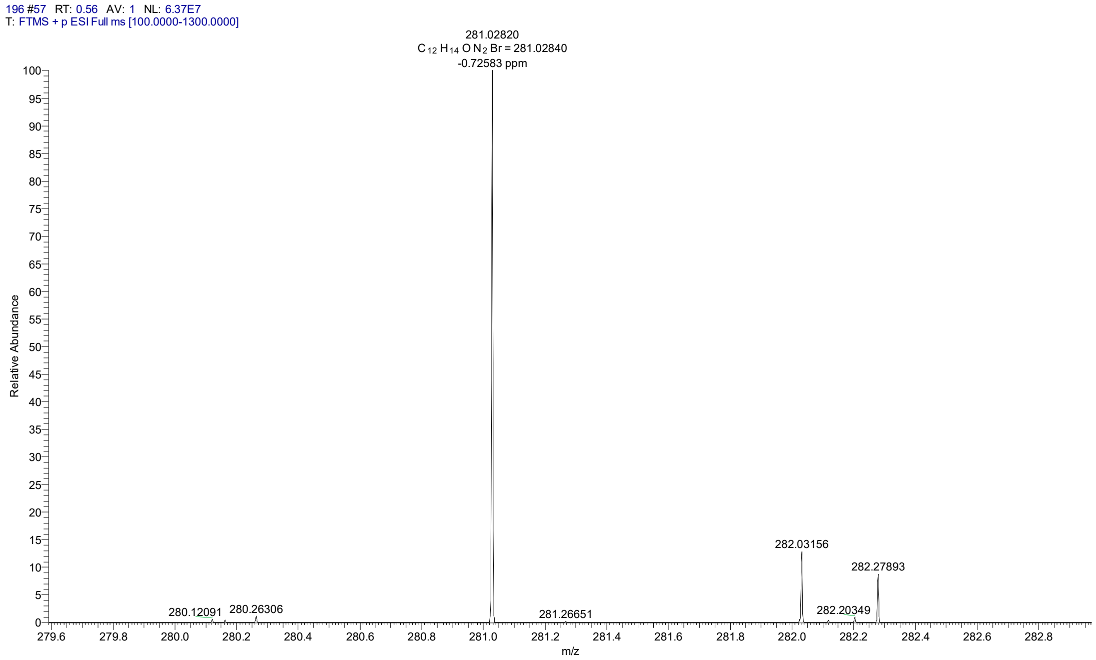

HRMS of compound **B19**
